# Exploring theoretical policy options for reducing socioeconomic inequalities in multimorbidity: A microsimulation study in England from 2019–2049

**DOI:** 10.1177/26335565261441403

**Published:** 2026-06-23

**Authors:** Anna Head, Max Birkett, Kate Fleming, Chris Kypridemos, Martin O’Flaherty

**Affiliations:** 1 Department of Public Health, Policy and Systems, Institute of Population Health, 4591 University of Liverpool, Liverpool, UK; 2 NHS England, 496847 National Disease Registration Service, London, UK

**Keywords:** multimorbidity, prevention, inequalities, public health, microsimulation

## Abstract

**Background:**

Projections suggest that the number of adults living with multimorbidity will continue growing in the coming decades. Little is known, however, about the potential impact of prevention policies on multimorbidity.

**Methods & findings:**

We applied a validated microsimulation model of multimorbidity accumulation to simulate theoretical scenarios of health improvement and inequality reduction in England over 30 years (2019-2049), compared to a baseline scenario of continuing patterns in accumulation. Four theoretical scenarios were based on Benach et al.’s typology of health policies: 1) targeted intervention on the worst-off; 2) universal policy + additional focus on the gap; 3) redistributive policy; 4) proportionate universalism; plus an idealistic fifth scenario completely removing socioeconomic inequality in transition times between states. We selected a target of 3% reduction in mortality for scenarios 1-4, based on reductions seen from tobacco control policies. Outputs compared were: difference in 2049 projected prevalence and numbers compared to baseline, total cases prevented/postponed compared to baseline, and expected years lived without multimorbidity at age 30. Our results suggest that gains in levelling socioeconomic inequalities in health would prevent/postpone multimorbidity cases and reduce relative health inequalities among those aged <65. However, this would also likely lead to increased absolute numbers living with multimorbidity overall.

**Conclusions:**

Our theoretical modelling suggests effective and equitable policies have potential to reduce the population-level burden of multimorbidity, postponing a substantial number of multimorbidity cases, particularly before age 65. This is, however, likely to lead to greater absolute numbers of multimorbidity cases as individuals live for longer.

## Why was this study done?

• The prevalence of multimorbidity in England has been rising in recent years, with onset at younger ages than in the past.
• Socioeconomic inequalities in multimorbidity prevalence are also well documented, and may be widening.
• Research and policy on multimorbidity is predominantly focused on improving management and care, with less focus on multimorbidity prevention.
• Understanding the potential impact and trade-offs of different strategies to reduce multimorbidity and narrow socioeconomic inequalities is beneficial for the planning of policy and care provision.

## What did the researchers do and find?

• We modelled the potential impact of a range of theoretical prevention strategy scenarios to reduce the total burden of multimorbidity and narrow its socioeconomic inequalities among the adult English population.
• Our model used primary care data from a random sample (n = 762,803) of adults in England from 2004-2019, and we simulated our scenarios for 30 years from 2019.
• We found that under all scenarios, the reductions in both overall cases of multimorbidity and inequalities were concentrated among those aged under 65.

## What do these findings mean?

• Our theoretical modelling study suggests that prevention policies with a focus on reducing health inequalities have the potential to prevent or postpone substantial numbers of multimorbidity cases, with the majority being among those aged under 65.
• Even with effective prevention strategies, multimorbidity prevalence and absolute numbers living with multimorbidity are still likely to rise as increased survival results in individuals living for longer.
• Our study has focused on comparing different theory-based prevention strategies to achieve a given reduction in deaths; we have not used empirical evidence on the impact of real-life policies and therefore our results are illustrative of the potential trade-offs, not expected outcomes.

## Introduction

Multimorbidity – having two or more long-term conditions – has been described as a “defining challenge” for health systems,^1^ which are traditionally focused on single conditions.^2^ Multimorbidity, also referred to as multiple long-term conditions, presents challenges for individuals in terms of health outcomes, quality of life, and wellbeing^3,4^ and the resulting poor health and functional impairment are associated with earlier exit from work.^5,6^ It is also complex to manage and resource-intensive for health and social care systems.^7^

Socioeconomic inequalities in health outcomes, including multimorbidity, are well-documented in the literature.^8,9^ Social inequalities in health are driven by environmental, structural, and societal factors which are amenable to change and, by definition, are “systematic, socially produced (and therefore modifiable) and unfair”.^10^ The health of the most advantaged groups can be seen as a readily attainable benchmark for the level of health that is possible for all groups – illustrating the possibilities for prevention. In addition to widening inequalities, there is also evidence of an increasing number of individuals living with multiple long-term conditions and at younger ages than in the past,^8^ with projections that this increase will continue.^11^ Attention is therefore needed to both prevent future accumulation of conditions and also to ensure that inequalities are narrowed: two key goals of health policy.^12^

The distribution of risk for ill health is spread throughout the population but is unevenly distributed, with some individuals and groups at higher risk. Theoretical frameworks describing how this distribution can be altered are useful for the development of effective and equitable health policy. A framework developed by Joan Benach and co-authors ties together different approaches proposed by Geoffery Rose, Hillary Graham, and Michael Marmot into a single typology of policies for improving health and reducing health inequalities.^13–16^ They summarise these approaches into four scenarios: 1) targeted interventions for the most deprived group and a focus on the health gap; 2) universal policies with an additional focus on the health gap of the most deprived group; 3) redistributive policies addressing the causes of ill health benefitting all but the most advantaged group; 4) proportionate universalism – universal policies where the benefits increase for those with increasing levels of deprivation.

In this study, we aim to simulate a set of theoretical prevention and health inequality reduction scenarios to explore their potential impact on inequalities in the future burden of multimorbidity. Our scenarios are compared to a baseline scenario that assumes past patterns in multimorbidity progression will continue. Whilst our scenarios do not estimate the effectiveness of specific real-life policies, in the absence of empirical evidence on prevention policies and their effectiveness at reducing multimorbidity onset, modelling a typology of scenarios can provide initial insights for the scoping of policy options, the timescales over which change is likely, and the potential scale of reduction in health inequalities.

## Methods

### Overview

We used a validated continuous-time microsimulation model using primary care records from 762,803 adults in England to describe inequalities in the accumulation of multimorbidity.^11^ We modelled a synthetic population of adults aged 30-90 based on official population estimates for England and projected individuals for 30 years from 2019 to 2049 under five theoretical scenarios, compared to a baseline scenario of continuing patterns in multimorbidity accumulation (previously published).^11^ The model data sources and methods are summarised below, with key scenario assumptions outlined in Table 1; a technical summary of the model is included in S1 Technical Appendix, and code for analysis and implementation is available via github: https://github.com/annalhead/mm_accum_microsim.

**Table 1.** Summary of how transition times for each IMD quintile group were altered for each scenario.

Scenario		Summary	​	IMD quintile group (1 least deprived; 5 most deprived)			
#	name	​	1	2	3	4	5
1	Targeted	Target solely the worst-off groups: Reduce the gap between IMD4&5 and IMD3	As baseline	As baseline	As baseline	Reduce the gap in transition times with IMD3 by 73.9%	Reduce the gap in transition times with IMD3 by 74.0%
2	Universal with a focus on the gap	Target the whole population, with an additional focus on the most deprived groups	Increase the length of time to transition by 2.8%	Increase the length of time to transition by 2.8%	Increase the length of time to transition by 2.8%	Increase the length of time to transition by 5.4%	Increase the length of time to transition by 9.4%
3	Redistributive	Targets the unequal distribution of causes of ill health, and therefore does not benefit the least deprived	As baseline	Increase the length of time to transition by 3.2%	Increase the length of time to transition by 3.5%	Increase the length of time to transition by 4.0%	Increase the length of time to transition by 4.9%
4	Proportionate universalism	Targets the whole population, with increasing benefits along the social gradient that are proportional to the level of disadvantage	Increase the length of time to transition by 2.2%	Increase the length of time to transition by 2.7%	Increase the length of time to transition by 3.0%	Increase the length of time to transition by 3.5%	Increase the length of time to transition by 4.3%
5	Removal of inequalities	Socioeconomic inequalities in transition times between states are completely removed	As baseline	As IMD1	As IMD1	As IMD1	As IMD1

NB: The improvement values for scenarios 1-4 were generated to achieve a 3% reduction in cumulative deaths prevented/postponed compared to the baseline scenario, taking into account the socioeconomic gradient in 2019 age-sex standardised prevalence of basic multimorbidity in the baseline scenario.

Sensitivity analysis A: No improvements in transitions to mortality (all scenarios); Sensitivity analysis B: No improvements among the healthy population (all scenarios).

### Model inputs

To derive the microsimulation inputs, we used anonymised primary care records from a random sample of adults from the Clinical Practice Research Datalink (CPRD) Aurum database,^17^ linked to quintiles of the 2015 English Index of Multiple Deprivation (IMD) based on residential postcode for a measure of relative socioeconomic deprivation.^18^ Included individuals (n = 762,803) were aged 18 or over, were born between 1919 and 1986, had at least one year of registration at a GP practice contributing to CPRD between 2004 – 2019, and had no missing sociodemographic information.

We used primary care records to identify cases of 211 chronic conditions (as described in previous work^8,11^), from which we classified individuals into four states of multimorbidity accumulation: 1) healthy, 2) one chronic condition, 3) basic multimorbidity (2 or more chronic conditions), and 4) complex multimorbidity (3 or more conditions across 3 or more body systems). We assumed that all conditions were life-long (no recovery), that interim states could not be skipped, and that individuals could die from any state, resulting in a total of 7 uni-directional transitions (see S1 Technical Appendix Figure A). For each transition, we fitted parametric survival analysis models with age as the time frame to estimate transition times by sex, quintile of IMD, geographic region, and 5-year birth cohort. Akaike’s Information Criteria and visual inspection of non-parametric estimates against parametric distributions were used for model selection and parameterisation. We predicted values from the fitted survival analysis models at deciles of the normal distribution for each transition. These were used as inputs for simulating the transition times within the microsimulation. We have assumed that the transition times derived by age and birth cohort from CPRD data between 2004-2019 remain constant over time.

### Synthetic population

The synthetic population to be simulated was created to represent the English adult population structure (ages 30-90) and sociodemographic characteristics in 2019, based on the mid-year population estimates for 2019 stratified by sex, IMD quintile, single year of age, and region obtained from the Office for National Statistics (ONS).^19^ Sex, IMD quintile, and region characteristics were assigned at the start of the model, and not updated. For each year from 2020-2049, a new cohort of 30-year-olds was introduced into the model based on ONS population projection estimates for 30-year-olds in that year.^20^ To reduce computational requirements, a 1% stratified sample of the population was used (N = 357,159), and results were scaled back up after simulation. As we explore results by age-group, and not intersectional sub-groups, a 1% sample is sufficient for stable estimates.

The starting health state of simulants was randomly assigned based on the proportion of individuals by sex, 5-year age group and IMD quintile in 2019 in the CPRD Aurum sample used for this study. Synthetic population characteristics and starting health states are presented in S1 Table O.

### Scenarios

We developed our prevention scenarios based on Benach et al.’s typology of health inequality reduction policies,^13^ as it provides a useful theoretical framework for examining how different policy types may impact health inequalities.^21^

Scenario 0. **Baseline**: applying the transition times derived from the CPRD Aurum sample data to the ONS 2019 mid-year population estimates.

Scenario 1. **Targeted intervention on the worst-off**: the intervention, and therefore the benefits, is targeted solely at the worst-off groups, reducing the health outcomes gap (absolute and relative) between the most and the least deprived groups.

Scenario 2. **Universal policy with an additional focus on the gap**: the intervention targets the whole population, regardless of deprivation, but there is an additional focus on greater improvements in the health of those from the most deprived groups.

Scenario 3. **Redistributive policy**: the intervention targets the causes of ill health, which is unequally distributed among socioeconomic groups, and as such, the least deprived groups do not benefit.

Scenario 4. **Proportionate universalism**: an intervention that is universal but which has increasing benefits along the social gradient that is proportional to the level of disadvantage.

Scenario 5. **Removal of inequalities**: We have also added a fifth ideal scenario, which considers the situation in which socioeconomic inequalities in transition times between states are completely removed.

Table 1 summarises how the distribution of transition times between states was altered for each IMD quintile under each scenario.

To parametrise scenarios 1-4, we set a target of preventing or postponing 3% of projected cumulative deaths in the baseline scenario between 2019-2049. This target was chosen so that the distribution of effects could be compared between scenarios, and agreed as a realistic target based on 10-year reductions in mortality from 2009-2019 in England.^22^ We then iteratively searched for a level of improvement that would result in the target reduction in deaths. We used the 2019 baseline differences in age-sex standardised prevalence of basic multimorbidity between the IMD quintiles to generate the ratio of improvements between quintiles.

These scenario implementations are illustrative of the theoretical policy typologies – the actual values of the improvements modelled are essentially arbitrary: scenario magnitudes do not correspond to efficacy of a real-world policy. We chose these specific scenario implementations with the aim of modelling examples that can demonstrate differences in how the approaches may impact the future burden of multimorbidity.

### Sensitivity analyses

We ran the following two sensitivity analyses to demonstrate alternative implementations of the scenario types:

A. No improvements in mortality: For all scenarios, we applied the improvement only to transitions between health states and not to death.
B. No improvements among the healthy population: For all scenarios, we applied the improvement only to those in either the initial condition, basic multimorbidity or complex multimorbidity health states.

### Outcomes for comparison

We calculated and compared the following outcome measures:

1. Difference compared to baseline in prevalence and absolute numbers in each health state in 2049 by IMD quintile group and by broad age group.
2. Cases prevented/postponed by state between 2019 and 2049, overall, by IMD quintile group, and by broad age group.
3. Median years to be lived without multimorbidity and years to be lived without complex multimorbidity at age 30, overall, and by IMD quintile group.
4. Absolute inequalities in multimorbidity prevalence in 2049, calculated using the slope index of inequalities (SII), a regression method that calculates the inequalities between the most and least deprived groups, accounting for the size of the groups and the intermediate IMD groups.^23^ Because more deprived IMD quintile groups are, on average, younger, we present this by 10-year age groups.

Scenario results are reported with first-order Monte Carlo uncertainty: the median and 95% uncertainty intervals from 100 model iterations (10 for sensitivity analyses) with the parameter inputs fixed at the mean for each variable.

### Validation and calibration

We validated our model against our original CPRD data of observed transition times (see S1 section 1.12. Microsimulation model validation and calibration for full details). We ran the microsimulation on a subset of individuals (42·2% of the total individuals included in this study) in our CPRD Aurum sample for whom we had at least 10 years of follow-up or who died within 10 years of entering the study. The simulated proportion of individuals in each health state at 10 years by sex represented the observed outputs after 10 years well, with less than a 1 percentage point difference between observed and simulated prevalence for all states. We calibrated the model to reduce these differences to less than a 0·01 percentage point difference for all states apart from death, which remained slightly underestimated.

### Ethical approval

The study protocol was approved by the CPRD Independent Scientific Advisory Panel [protocol 19_173].

## Results

### Numbers and proportions by state in 2049

**Figure 1.**
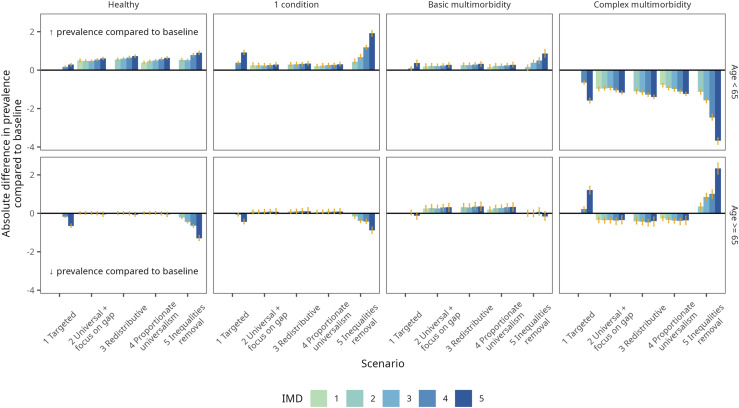
Difference in 2049 projected multimorbidity prevalence by scenario, IMD quintile, and broad age-group, compared to the baseline scenario. IMD = Index of Multiple Deprivation (1 = least deprived). Error bars represent 95% uncertainty intervals.

Figure 1 presents differences in 2049 projected prevalence by scenario, IMD quintile group and broad age groups compared to the baseline scenario. Among those aged under 65, the simulated prevalence of complex multimorbidity decreases compared to the baseline projections, driven by increases in the three preceding health states. Among those aged 65 and over, the scenarios that improve health across the population and reduce inequality (2 universal + gap, 3 redistributive, 4 proportionate universalism) do slightly reduce the proportion of those living with complex multimorbidity. In contrast, the targeted and inequalities removal scenarios (scenarios 1 and 5 respectively) show decreases in basic multimorbidity prevalence and increases in complex multimorbidity prevalence compared to the baseline scenario. All scenarios increased the proportion of under 65s living without multimorbidity in 2049 across all IMD quintiles impacted by the scenario; for the 65 and over age group, only the targeted and inequalities removal scenarios (scenarios 1 and 5) decreased the proportion living without multimorbidity (S2 supporting information Table A).

**Figure 2.**
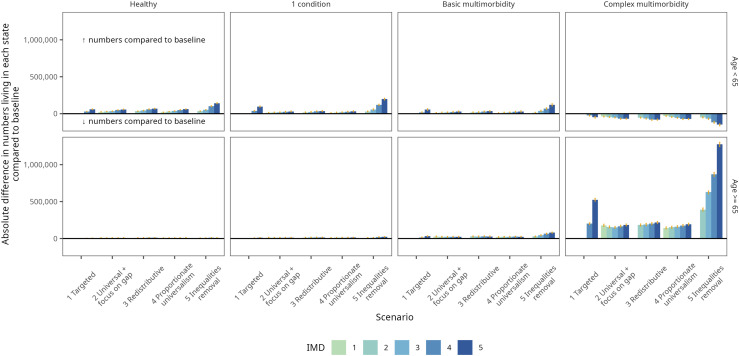
Difference in 2049 numbers in each health state by scenario, IMD quintile, and broad age-group, compared to the baseline scenario. IMD = Index of Multiple Deprivation (1 = least deprived). Error bars represent 95% uncertainty intervals.

Figure 2 shows how these differences in prevalence are partly driven by overall health improvements leading to greater numbers of individuals in most health states and, therefore, a greater overall projected population when compared to the baseline scenario. For all IMD quintile groups, however, all types of improvement scenarios could decrease the absolute number of individuals under 65 living with complex multimorbidity. In addition, all scenarios would increase the absolute number of adults living without multimorbidity in 2049 for all IMD quintiles impacted by each scenario (S2 supporting information Table B).

### Cumulative prevented or postponed cases by 2049

**Figure 3.**
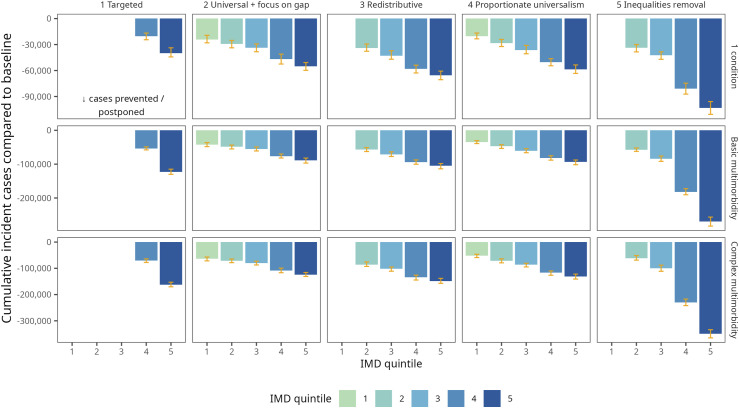
Total incident cases prevented/postponed 2019-2049 compared to the baseline scenario. IMD = Index of Multiple Deprivation (1 = least deprived quintile); negative numbers are cases prevented/postponed compared to the baseline scenario. Error bars represent 95% uncertainty intervals.

To examine the cumulative impact of the scenarios over the 30-year projection period, Figure 3 presents the cumulative incident cases between 2019 and 2049 prevented/postponed under each scenario compared to the baseline scenario, by IMD quintile (Figure 3). In all five scenarios, more overall cumulative cases are prevented or postponed in the more deprived IMD quintiles.

Looking at cumulative cases prevented or postponed split by broad age group (S2 supporting information Figures A & B), the majority of health improvements modelled in these scenarios would occur among those aged under 65, and this is the case for all five IMD quintiles. The postponement of these cases at younger ages leads to a greater number of incident cases among the 65 and over age group (S2 Figure A), even where the proportion living with multimorbidity is lower in the theoretical scenarios than the baseline (Figure 1).

### Years lived without multimorbidity

**Figure 4.**
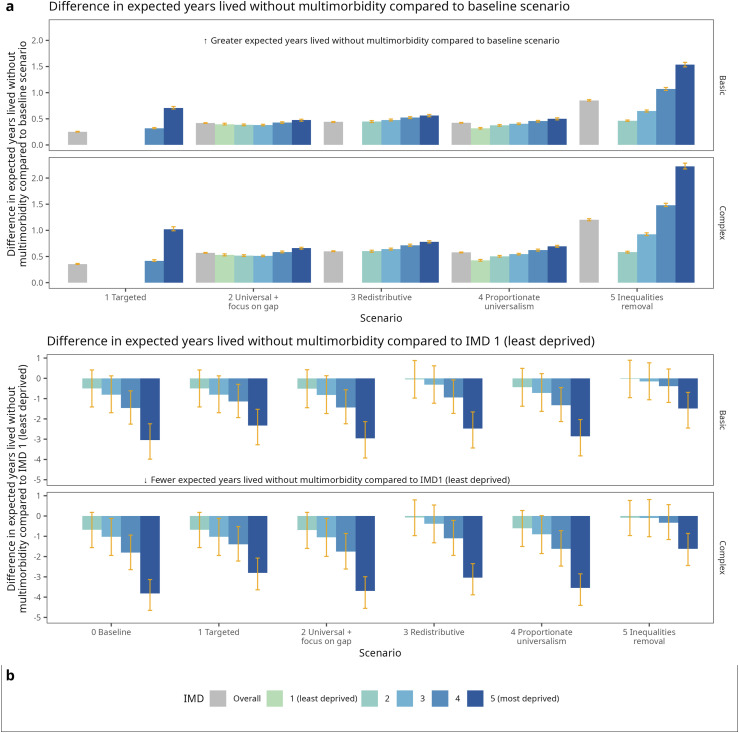
Difference in median expected years lived without basic and complex multimorbidity at age 30: (a) by IMD quintile and scenario, compared to baseline scenario; (b) by IMD quintile and scenario, compared to the least deprived IMD quintile (IMD1). IMD = Index of Multiple Deprivation (1 = least deprived quintile). Error bars represent 95% uncertainty intervals.

All five theoretical policy scenarios increase the projected expected median number of years lived at age 30 without multimorbidity compared to the baseline scenario (Figure 4(a)). Overall, the scenarios that impact a larger proportion of the population would increase the expected median number of years without multimorbidity the most. As the impact of these redistributive and universal scenarios is distributed across the population, the impact on increasing expected years without multimorbidity amongst the most deprived is smaller. Figure 4(b) demonstrates the difference between the expected median number of years lived at age 30 without multimorbidity in IMD quintile groups 2-5 compared with IMD quintile 1. Whilst all scenarios reduce the inequalities compared to the least deprived quintile to some extent, a targeted scenario has the biggest impact amongst the more deprived quintile groups, therefore compressing the slope of the socioeconomic gradient. A redistributive scenario, on the other hand, which improves health outcomes for all but the least deprived, could substantially reduce inequalities between the middle quintiles and the least deprived. Similar inequality patterns are seen for both multimorbidity types, with a slightly greater magnitude in years spent without complex multimorbidity than those without basic multimorbidity.

### Absolute inequalities in 2049 multimorbidity prevalence

**Figure 5.**
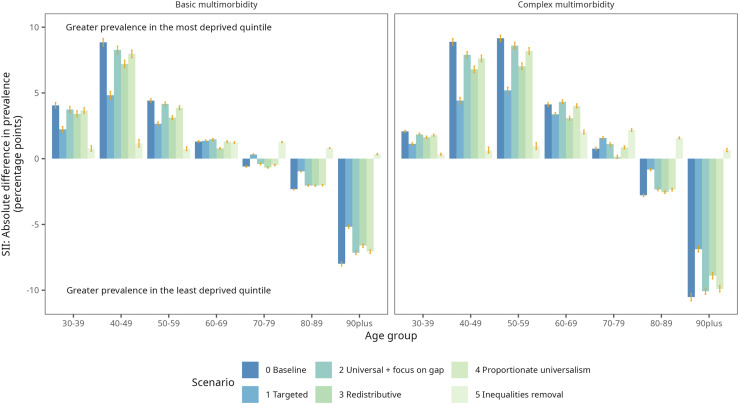
Absolute inequalities in the crude prevalence of basic and complex multimorbidity by scenario and 10-year age group in 2049. Absolute inequalities were derived using the slope index of inequalities (SII). The y-axis shows the absolute difference in projected prevalence between the most and least deprived quintiles for each scenario. 0 signifies no absolute inequalities. Please note that the numbers of people in each health state and age group are not evenly distributed. Health states are not mutually exclusive: basic multimorbidity encompasses all simulants with at least two chronic conditions. Error bars represent 95% uncertainty intervals.

In almost all age groups, absolute inequalities in projected 2049 multimorbidity prevalence were reduced compared to the baseline for all scenarios (Figure 5). The magnitude of inequality reduction varies greatly by scenario and age group. Among the under-60s age groups and under-70s for complex multimorbidity, the two scenarios with the largest reductions in absolute inequalities are the targeted and inequalities removal scenarios (scenarios 1 and 5), driven by slower transition times between health states for the more deprived IMD quintiles. The patterns are more mixed after age 60, with these two scenarios increasing absolute inequalities for the 70-79 age group and for those aged 80+ in the inequalities removal scenario (scenario 5).

### Sensitivity analyses

For all sensitivity analyses, the patterns between scenarios remained as in the main analysis. Table 2 summarises the main findings of the two sensitivity analyses compared to the main analysis (Table 2).

**Table 2.** Summary of sensitivity analysis findings compared to the main analysis.

Analysis	Total cases prevented/postponed(2019–2049)	Median years without MM at age 30	Scenario ranking
Main analysis	Reference	Reference	Reference
Sensitivity A *No improvement in mortality transitions*	↑ **Greater** than main analysis (S2 Supporting Information Figure C)	↓ **Less** than main analysis (S2 Supporting Information Figure D)	Preserved
Sensitivity B *No improvement among the healthy population*	↓ **Less** than main analysis (S2 Supporting Information Figure E)	↓ **Less** than main analysis (S2 Supporting Information Figure F)	Preserved

MM = multimorbidity. Sensitivity analysis A applied improvements only to transitions between health states and not to mortality. Sensitivity analysis B applied improvements only to those already in a disease state (initial condition, basic multimorbidity, or complex multimorbidity). For detailed results see S2 Supporting Information Figures C–F. For all sensitivity analyses, the relative differences between the five scenarios remained consistent with the main analysis.

## Discussion

This study has used a validated microsimulation model to simulate five theoretical policy scenarios to improve health and reduce inequalities in the context of multimorbidity among adults aged 30 and over in the English population between 2019 and 2049. Our results suggest that gains in levelling socioeconomic inequalities in health from any of these scenarios could prevent or postpone multimorbidity cases and reduce relative health inequalities in the population aged 30-65. However, as prevention policies likely reduce mortality rates as well as incidence rates, then these scenarios would also likely lead to people living longer with multimorbidity, and an increase in the absolute number of people living with basic and complex multimorbidity overall.

Whilst it may seem counterintuitive that prevention policies could result in larger numbers overall living with multimorbidity, this is a product of the relationship between incidence, prevalence and survival. Under our assumption that chronic conditions are lifelong (i.e. no recovery), prevention policies can have two impacts: 1) delaying the onset of conditions (incidence), and therefore slowing the progression of multimorbidity – this decreases the number of new multimorbidity cases; 2) increasing survival (reducing mortality), so that people live for longer in each health state, including with multimorbidity – this increases the number of people living with multimorbidity (and overall population size).^24^ As annual incidence is a small proportion of prevalent cases, the impact of improving survival outweighs the impact of incidence reductions, resulting in more people overall with multimorbidity in our scenarios. The relationship between incidence and prevalence also explains the seemingly opposing impacts between the under and over 65 age groups: as prevention postpones onset of disease as well as increase survival, new multimorbidity cases are more likely to occur at older ages, particularly for complex multimorbidity.

There are few comparative studies within the literature of similar scenario modelling. A recent study combined two projection models to simulate hypothetical scenarios of differing trends in disability progression and recovery and the impact on levels of dependency, care costs, and life expectancy in adults over 65 in England.^25^ PACSim-CPEC projected that under ‘optimistic’ scenarios comprising slower disability progression and faster recovery, the numbers of older people at all stages of dependency would likely decrease. This is the opposite side of our findings that health improvements could potentially lead to greater numbers of older adults living both with and without multimorbidity. One key difference between our approaches, which may explain this discrepancy, is that we have applied improvements to transitions to death, as well as transitions between living states. Our sensitivity analysis restricting improvements to transitions between living states projects fewer people with complex multimorbidity and shows some similarities with the PACSim-CPEC patterns.

### Strengths and limitations

A key strength of our study is that it models all adults in England aged 30 and above and how they accumulate chronic conditions over 30 years between 2019-2049, enabling a broad, population-level overview of the potential for reducing the future multimorbidity burden. Like all simulation models, we have made assumptions and simplifications in developing our microsimulation. A brief summary of key points is included here; further details are in Supplementary Materials S1.13. We have assumed that our source data from primary care health records is representative of the general population. Due to lack of longitudinal information on deprivation or region within CRPD, we assumed that individuals in our model do not change regional area or relative quintile of deprivation. Additionally, increasing the model complexity to account for mobility would not change the comparison of the theoretical scenarios. We assumed all conditions are lifelong once diagnosed and recorded, and did not allow recovery from conditions such as asthma, anxiety or depression, or obesity. The quality of the underlying data impacts our results and the conclusions that can be drawn. For example, health-seeking behaviour, frequency of general practice visits, length of registration, propensity to seek care, and pre-existing conditions all influence the likelihood of initial and subsequent disease diagnosis, and these factors may differ by socioeconomic deprivation.

For resource reasons, we did not have linkage for our primary care data to secondary care, although the majority of chronic health conditions are managed within primary care and are, therefore, likely to be recorded. In recent work examining the setting of diagnosis recording in linked primary and secondary care records, an estimated 85% of all diagnoses were present in primary care, and this underestimation may be gently increasing by deprivation.^26^ We may, therefore, slightly underestimate multimorbidity prevalence and baseline inequality in our current study. Similarly, we were unable to link to ONS mortality records; however, the presence/absence of death is generally well recorded within CPRD.^27^ Due to issues with data quality in recording of ethnicity, we were unable to investigate ethnic inequalities in scenario outcomes.

Our modelling scenarios have not looked at pathways through which socioeconomic inequalities in multimorbidity develop, and further work to understand these is important for developing successful, equitable policies to act on these pathways.^28^ Instead, we modelled scenarios based on commonly used theoretical frameworks for understanding and explaining socioeconomic inequalities in health. We have assumed that the transition times derived by age and birth cohort from CPRD data between 2004-2019 remain constant over time, and do not take into account advancements in health systems or improvements in risk factor trends. These advancements may have differential effects across socioeconomic groups, for example reductions in tobacco use will have bigger impacts on the most deprived groups, whilst projected improvements in systolic blood pressure has greater impacts among the least deprived.^24^ We focused on socioeconomic inequalities within this work and assumed that the modelled improvements were applied evenly across the life course. However, given that there are stark inequalities by sex, ethnicity and between regions, it is likely that policies to target these other sociodemographic inequalities would also have the potential to reduce the multimorbidity burden at the population level.

Given the theoretical nature of our scenarios, we cannot draw concrete conclusions about actual policies or recommend specific policy action. The size of the health improvements and inequalities reduction are, to a certain extent, driven by our modelling choices, and not all conditions will be equally amenable to prevention strategies. Empirical evidence on the effectiveness and impact on inequalities of specific policies at reducing multimorbidity incidence is scarce. There are, however, existing examples of implemented health policies that can be mapped to each theoretical scenario (see Supplementary Materials S1.13). Although our fifth scenario of removing socioeconomic inequalities (removing socioeconomic inequalities in transition times) is idealistic, it demonstrates the potential scope for health improvement if there was no social stratification between groups in society.^29^ An inherent assumption within this scenario is that disadvantaged groups will be able to reach the same level of health progression as those from the least deprived group and that past histories of deprivation will have no future impact. This likely overestimates the impacts of removing social stratification.^30^ Beyond theory, empirical research on policy effectiveness with multimorbidity outcomes will be required to select the most feasible, evidence-based, effective, cost-effective and equitable policies. It is clear from existing evidence that a focus on individual-level behaviour change will be insufficient to address current socioeconomic inequalities adequately.^31^ In future, our model could be adapted to incorporate effect sizes from real-world evaluations of specific inequalities reduction interventions.

### Policy implications

The large burden of multimorbidity on individuals and the health and social care systems means that understanding whether and to what extent prevention efforts may impact the future burden of multimorbidity is essential for supporting health and social care system planning. Whilst the theoretical scenarios we have modelled are not able to support decision-making as to specific policies, they illustrate that prevention strategies can impact the progression of multimorbidity. However, the scale of the problem, and the chronic nature of long-term conditions, means that millions of people will still live with multiple chronic conditions, and complex care needs are likely to remain high in the coming decades. Policies and strategies for managing multimorbidity will therefore be required alongside prevention efforts.

Our scenarios also illustrate the potential trade-offs between different approaches to health improvement and health inequalities reduction. By using a fixed target of reductions in mortality, our scenarios demonstrate how the benefit of the same overall improvement in health could be distributed across the population (see Supporting Information S2 Table C). Scenarios that target the whole population, such as proportionate universalism (scenario 4) or universal policies with a focus on the gap (scenario 2), produce greater overall improvements in cases prevented or postponed and in expected years lived without multimorbidity. However, targeted approaches (scenario 1) and the removal of inequalities (scenario 5) are more effective at reducing absolute socioeconomic inequalities in multimorbidity prevalence, particularly among those aged under 60. This trade-off is well recognised in the health inequalities literature,^13,15^ and the choice between these approaches is ultimately a normative policy decision. Our findings suggest that any of these theoretical prevention strategies could prevent or postpone a substantial number of multimorbidity cases before age 65, but that no single approach simultaneously optimises all outcomes. Beyond theory, empirical research on policy effectiveness with multimorbidity outcomes will be required to identify the most feasible, cost-effective, and equitable policies.

These findings also highlight questions of value judgements: are all gains valued equally? Do we prioritise effectiveness over reducing health inequalities? How should resources be distributed across the population? While our scenarios do not answer these value questions, they explicitly illustrate some of these tensions inherent within policy decisions. For example, even if prevention strategies do not decrease the total number of people with multimorbidity substantially, delaying onset into later life has benefits at both individual and societal levels. These gains are likely to be greatest among the younger population as the primary prevention scenarios we have modelled focus on reducing the incidence of disease. Stronger associations between multimorbidity and mortality have been reported among those aged under 65 as compared to older adults.^32^ Poor health can affect the ability to work or the type of work to be undertaken,^33^ and the financial consequences can worsen health further.^29^ Those from more deprived areas are more likely to have more complex healthcare needs earlier but are also more likely to be in occupations that are more physically demanding, have fewer adaptations, and have greater insecurity.^34^ At a local level, an area’s economic activity forms part of the income stream for providing local services such as social care and public health.^35^ Finally, we reported results for two age categories split at age 65 for succinct presentation. However, this simple dichotomy is more nuanced in reality. At least 1 in 9 adults over 65 are employed, which has increased over the past decades.^36^ Slightly over 10% of this age group provide unpaid care, almost half of whom provide at least 50 hours of unpaid care per week; rates of unpaid care are also higher in more deprived areas.^37,38^

In summary, although the price to pay for prevention success may be greater absolute numbers of people living with multimorbidity in the long term, increasing the number of years spent without multimorbidity is an important health policy goal. The identification and implementation of effective, equitable prevention policies will, therefore, become essential to address the future burden of multimorbidity equitably. Multimorbidity prevention needs to be one part of policies and strategies to address multimorbidity, alongside strategies to improve self-management, management by the health and social care system, and wider societal adaptations to support longer lives.

## Supplemental material

Supplemental material - Exploring theoretical policy options for reducing socioeconomic inequalities in multimorbidity: a microsimulation study in England from 2019–2049 Supplemental material for Exploring theoretical policy options for reducing socioeconomic inequalities in multimorbidity: a microsimulation study in England from 2019–2049 by Anna Head, Max Birkett, Kate Fleming, Chris Kypridemos, Martin O’Flaherty in Journal of Multimorbidity and Comorbidity.

Supplemental material - Exploring theoretical policy options for reducing socioeconomic inequalities in multimorbidity: a microsimulation study in England from 2019–2049 Supplemental material for Exploring theoretical policy options for reducing socioeconomic inequalities in multimorbidity: a microsimulation study in England from 2019–2049 by Anna Head, Max Birkett, Kate Fleming, Chris Kypridemos, Martin O’Flaherty in Journal of Multimorbidity and Comorbidity.

Supplemental material - Exploring theoretical policy options for reducing socioeconomic inequalities in multimorbidity: a microsimulation study in England from 2019–2049 Supplemental material for Exploring theoretical policy options for reducing socioeconomic inequalities in multimorbidity: a microsimulation study in England from 2019–2049 by Anna Head, Max Birkett, Kate Fleming, Chris Kypridemos, Martin O’Flaherty in Journal of Multimorbidity and Comorbidity.

## Data Availability

Data are available from the CPRD and ONS directly. A detailed protocol can be provided on request to AH. Code lists, analysis code, and model code are available on GitHub at https://github.com/annalhead. This work uses data provided by patients and collected by the UK National Health Service as part of their care and support.
