# Supplementary material for: Exploring theoretical policy options for reducing socioeconomic inequalities in multimorbidity: A microsimulation study in England from 2019–2049
Source: J Multimorb Comorb. 2026 Jun 23;16:26335565261441403. doi: 10.1177/26335565261441403 (PMC13305289; doi:10.1177/26335565261441403)
Supplement: Supplemental material - Exploring theoretical policy options for reducing socioeconomic inequalities in multimorbidity: a microsimulation study in England from 2019–2049 [file sj-pdf-1-cob-10.1177_26335565261441403.pdf]

S1 Supporting Information - Technical Appendix

This technical appendix summarises the development and implementation of a dynamic stochastic continuous-time microsimulation model for the accumulation of multimorbidity in adulthood. This Supplement acts as general, non-technical description of the model. Code for implementation is publicly available on github: https://github.com/annalhead/mm_accum_microsim.
This work builds on a previous publication[1], and much of this technical appendix information remains unchanged. We represent much of it in full here for ease of reference.

1.2. Multimorbidity definitions and implementation

As discussed extensively in past literature,[2,3] there is no 'gold standard' definition or implementation of multimorbidity: many definitions are necessarily pragmatic or developed for investigating specific outcomes. Based on our previous work,[4] we included 211 chronic conditions of interest, and implemented two definitions of multimorbidity: basic and complex. We present a summary here, full details can be found in our previous publication;[4] all code lists can be found here: https://github.com/annalhead/CPRD_multimorbidity_codelists and code for phenotyping can be found here: https://github.com/annalhead/CPRD_multimorbidity_trends.
Multimorbidity definitions

We implemented the following two definitions of multimorbidity:

- *Basic multimorbidity:* two or more chronic diseases from a list of 211 conditions (see below).
- *Complex multimorbidity:* three or more chronic conditions affecting three or more different body systems. This approach was proposed by Harrison et al.,[11] based on evidence that occurrence of diseases in multiple body systems may have a larger impact on health and healthcare use.

Chronic conditions

Given the focus of our project on socioeconomic inequalities in multimorbidity among adults, we chose to take a wide view of multimorbidity. As such, we aimed to incorporate conditions that present across adulthood (not just in older ages) in order to capture better multimorbidity treated in primary care.
We developed our list of 211 chronic conditions from the disease phenotypes derived by the CALIBER team and published by Kuan et al,[14] to which we applied the Academy of Medical Sciences definition of chronic conditions: "A physical non-communicable disease of long duration, such as a cardiovascular disease or cancer; A mental health condition of long duration, such as a mood disorder or dementia; or an infectious disease of long duration, such as HIV or hepatitis C".[2] We used long-term and active as the two criteria in assessing which conditions were of long duration. Active was defined as either i) using resources/impacting health, ii) conditions (e.g. cancer) that affect the subsequent risk of other conditions.
Our definition of multimorbidity included sequalae of certain conditions, e.g. diabetes and diabetic neuropathy, and did not allow 'recovery' from a condition. We chose to include these because of the differing impact on health outcomes associated with coexistence of

sequalae, and the likely impact of these conditions on future health, healthcare usage, or future risk of ill health.

The final list of 211 conditions was agreed by all members of the study team. Code lists for disease phenotypes developed by Kuan et al. were based on READ code terminology, as used in the CPRD GOLD database; we mapped these existing code lists to the code terminology used in the CPRD Aurum database (Appendix Text S1 of our previous publication for details)[4].

Discussion

We have included approximately four times more chronic conditions than other key UK multimorbidity studies[8,19], as we have not restricted conditions based on criteria such as intensity of resource use, prevalence in older ages, or strength of association with mortality. The value of this approach is that it encompasses conditions that are more likely to affect certain sub-populations, as well as including both preventable and non-preventable conditions. This is important for understanding the potential future demand for primary care, given that all the conditions recorded in primary care are a result of contact with the healthcare system. As with other studies[3], we have included conditions that are also risk factors for other conditions, such as hypertension and obesity, as both result in healthcare use for management.

Our definition of multimorbidity and list of included conditions means that presence of sequalae of certain conditions is counted as multimorbidity, e.g. diabetes and diabetic neuropathy; alcohol misuse and alcoholic liver disease. We chose to include these because of the differing impact on health outcomes associated with coexistence of sequalae, and the likely impact of these conditions on future health, healthcare usage, or future risk of ill health. Complications of diabetes, for example, can include damage to the kidneys, eyes, feet and teeth – affecting different body systems in different ways[5]. In addition, with the large number of conditions we have included, the marginal impact of each condition is likely to be relatively small,[6] and it is therefore unlikely that distinctly different patterns would emerge by changing slightly the specific included conditions. Similarly, how we implemented the code lists does not allow 'recovery' from a condition. The rationale being that once the diagnosis criteria are met, the included conditions are likely to impact on future health, healthcare usage, or future risk of ill health. Our analyses with complex multimorbidity as the outcome limit inclusion of sequalae to those that affect different body systems, allowing a more stringent comparison to other studies with shorter condition lists.

For resource reasons, we did not have linkage for our primary care data to secondary care, although the majority of chronic health conditions are managed within primary care and are therefore likely to be recorded. Similarly, we were unable to link to ONS mortality records, however the presence/absence of death is generally well recorded within CPRD.[7] If there are socioeconomic differences, however, in where conditions are recorded, this may have introduced bias into transition times by IMD quintile.

Differing disease severity and health care resource burden is inherent in this list of 211 conditions, and we have not included other measures of health status such as functional limitation or frailty. Despite this, given that this study is based on records from primary care, all occurrences of included conditions are a result of contact with health services – either directly via the GP, or through transfer of records from other settings such as secondary care discharge notes. As such, this is a signal of symptoms for which individuals are demanding healthcare, as well as instances of use of healthcare services. Given the aim of this study to investigate and project inequalities in the accumulation of multimorbidity throughout adulthood, we concluded that a broad approach to the inclusion of chronic conditions was appropriate.

1.3. Conceptualising the accumulation of multimorbidity into a multi-state model

A common way of describing pathways through multiple events in longitudinal data is through using a multi-state structure. Multi-state models are defined as having multiple discrete states, of which an individual can occupy one at any point in time[8]. A simple example of this is a three-state model where individuals can either be 1) healthy, 2) sick, or 3) dead. The structure of the multi-state model determines the possible health states and specifies how individuals can move between states. When an individual changes states, this is known as a transition, and the probability of these transitions based on explanatory variables of interest can be estimated from statistical models such as survival analysis models. This same multi-state structure, and the resulting transition probabilities, can then be used for building simulation models.
As the aim of this study was to explore the accumulation of multimorbidity, we conceptualised our model by splitting the accumulation of chronic conditions into the following 5 states:

- **Healthy**: Individuals with no pre-existing conditions from the 211 conditions of interest upon entry into the study were considered as healthy.
- **Initial chronic condition (1 condition)**: presence of only one chronic condition from the list of interest.
- **Basic multimorbidity (BMM)**: presence of any two chronic conditions phenotyped.
- **Complex multimorbidity (CMM)**: at least three conditions affecting at least three body systems.
- **Death**: the sole absorbing state.

We structured the model as unidirectional or progressive, and did not allow recovery or remission from any of the conditions as we assumed all were chronic. Transition to death was possible from any of the four living states. As a result, we conceptualised the accumulation of multimorbidity into a multi-state structure of five states (four living states, and an absorbing death state), with seven possible transitions (summarised in Figure A and Table A).

T# - Transition number

Figure A - Diagrammatic representation of the multi-state model

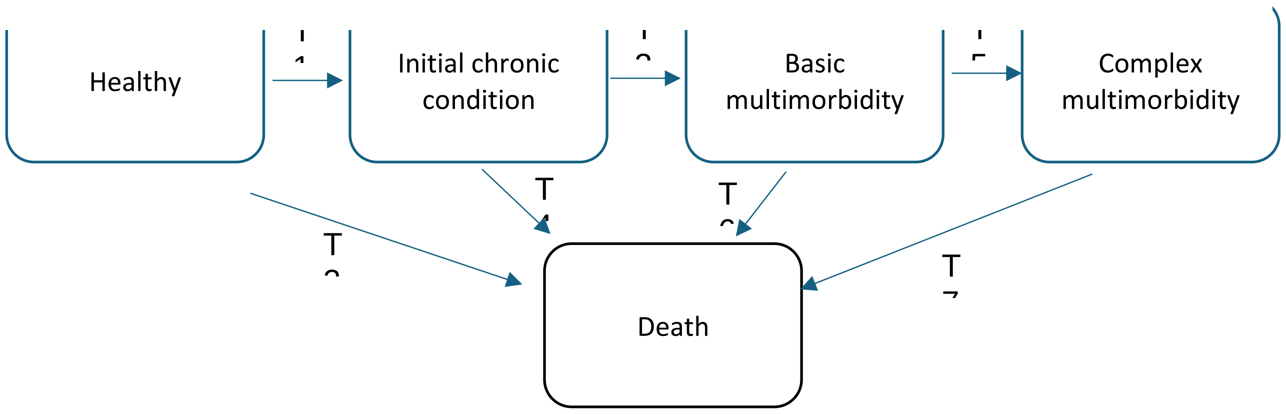

Table B - Summary of states and transitions used for conceptualising the accumulation of multimorbidity

**State Name**	Description	**Possible transitions to subsequent states**
**State 1: Healthy**	No chronic conditions	Transition 1 (T1): healthy -> incident condition

		Transition 2 (T2): healthy -> death
**State 2: Initial chronic condition**	One chronic (long term) condition	Transition 3 (T3): incident condition -> basic multimorbidity
		Transition 4 (T4): incident condition -> death
**State 3: Basic multimorbidity**	Two or more chronic conditions, but not meeting the complex multimorbidity definition	Transition 5 (T5): basic multimorbidity -> complex multimorbidity
		Transition 6 (T6): basic multimorbidity -> death
**State 4: Complex multimorbidity**	Three or more chronic conditions across three or more body systems	Transition 7 (T7): complex multimorbidity -> death
**State 5: Death**		None – absorbing state

1.4. Microsimulation model overview

The microsimulation model has three main inputs: the population to be simulated; a look-up table of the distribution of time to event for each transition; and a linear correlation matrix. A schematic diagram of the model and its inputs is illustrated in Figure B.

Population to be simulated: This can be specified. It needs to contain individual characteristics of: sex, IMD quintile, geographic region, 5-year birth cohort, age for the initial simulation year, and health state for the initial simulation year. Details of the synthetic population use in this paper are described in Section 1.4.3. Details of the population used for validating the model are described in Section 1.11

Look-up table of the distribution of time to event for each transition: We fitted parametric survival models for each of the seven transition models (described in Section 1.4.2.1). From this, we created a look-up table of the distribution of predicted time to each transition for all combinations of covariates (sex, IMD quintile, geographic region, and 5-year birth cohort) at quantile intervals of 0.1 (deciles), and conditional on survival to each year of age at entry (ages 30-90) (Section 1.4.2.2)[9]. The maximum predicted time was capped so that transitions did not occur after age 110; this is the maximum age used by the Office for National Statistics (ONS) life expectancy calculations[10]. The predicted transition times can be altered in alternative scenarios (see Section 1.5).

Linear correlation matrix: As people do not live indefinitely, time in each state is not independent of time spent in preceding states. For example, someone who lives for 70 years without any chronic conditions, is unlikely to spend 50 years living with one or more chronic conditions. To account for this, a linear correlation matrix of time in each state was calculated from the CPRD Aurum data sample based on past state history (Section 1.4.4).

For each simulant, the model runs sequentially through the states and transitions as per the multi-state structure in Figure A. Initial start state can be any of the living health states (healthy, initial condition, basic multimorbidity, complex multimorbidity). A random number is then selected for the quantile of the distribution (quantile x) for each transition. The correlation matrix is used to produce correlated random numbers for each individual (Section 1.4.4). If a calibration factor is used (Section 1.11), this also bounds the quantile number by the specified direction and amount. The quantile number selected determines the quantile of the distribution to be found in the look-up table for the simulant's sex, IMD quintile, region, 5-year birth cohort, and age at the start of the state. Linear interpolation is then used to calculate a time to event for the transition between quantile x and quantile x+1. The healthy, one condition, and basic multimorbidity states have two potential

transitions: to the next state, or to death. The random number process is conducted for both of these transitions, and the transition which occurs first is selected. Age is recalculated at entry into each new state. The model assumes independence of individuals.
The use of predicted times to event from the survival analysis models makes this a continuous timeframe microsimulation This approach of creating a look-up table of the distribution of time to transition has the advantage of conducting the computationally heavy analysis and prediction tasks only once. This is then stored and referred to throughout the simulation without having to rerun the predictions, resulting in fast and efficient simulation for both baseline and alternative scenarios. The alternative, commonly-used discrete approach, of calculating and applying yearly transition probabilities to individuals or cohorts is much more computationally intensive.

1.5. Model inputs

Study population

To derive the microsimulation inputs, we used primary care records from a 1m random sample of adults from the Clinical Practice Research Datalink (CPRD) Aurum database, linked to quintiles of the 2015 English Index of Multiple Deprivation (IMD) based on residential postcode for a measure of relative socioeconomic deprivation.

Figure C – Architecture of the microsimulation model

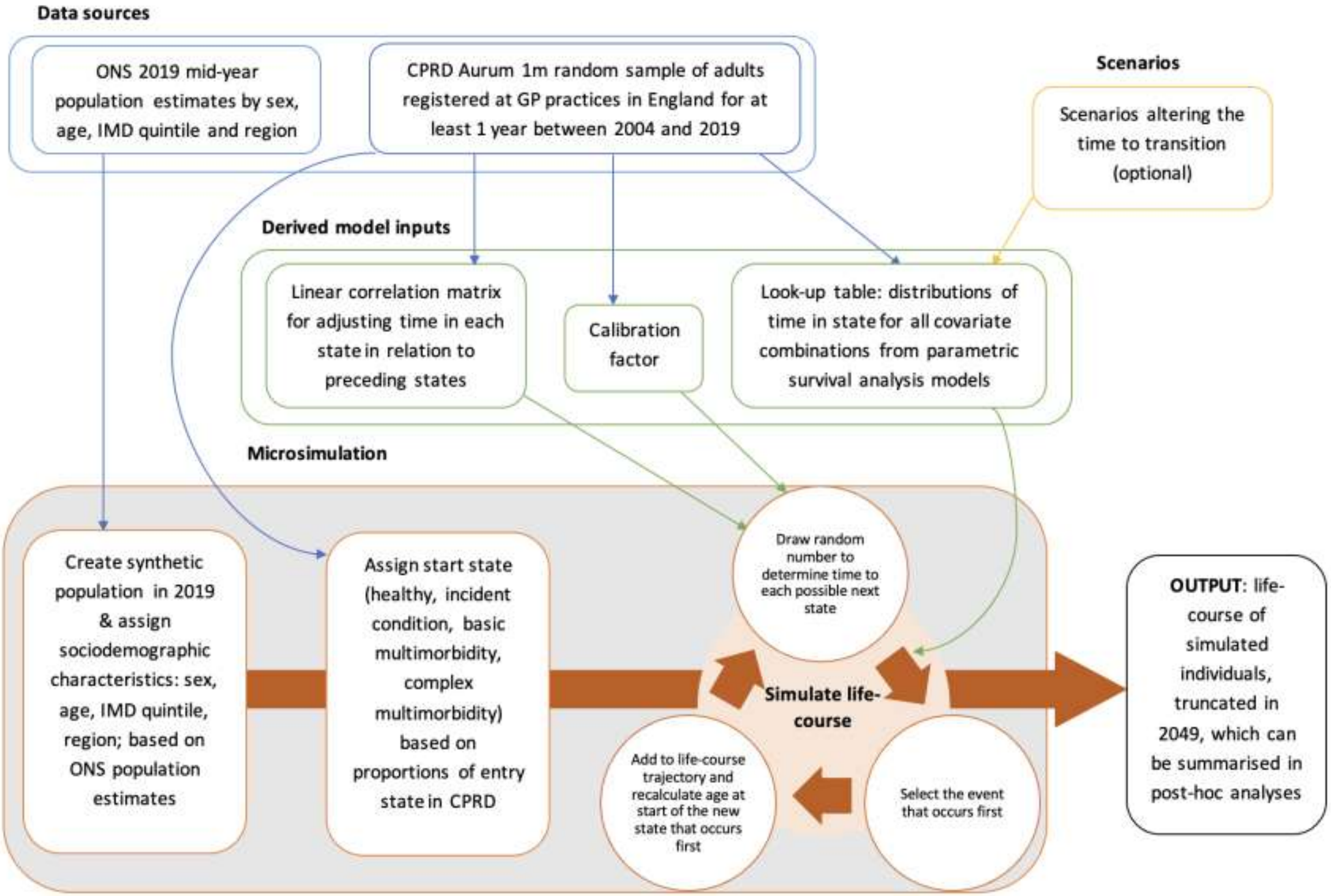

CPRD = Clinical Practice Research Datalink IMD = Index of Multiple Deprivation ONS = Office for National Statistics

The Clinical Research Practice Datalink (CPRD) Aurum database is an electronic health record repository of de-identified primary care records from participating GP practices in the UK[11,12]. In June 2020 (when the data for this work was extracted), CPRD Aurum covered 18% of the UK population (based on Office for National Statistics population estimates), and 14% of UK GP practices[13]. The English Index of Multiple Deprivation (IMD) 2015 is a composite measure of relative area-level deprivation, compiled of 37 indicators spanning seven domains of deprivation[14]. Data is combined at small geographical areas linked to postcode areas, which are then used to calculate fifths of relative deprivation (IMD quintiles). Quintiles of IMD were matched to individuals in CPRD by participants' residential postcode; linkage was done by NHS Digital.

The eligible study population was permanently-registered adults at contributing GP practices in England for any period between 1 January 2004 to 31 December 2019. Our study used an open cohort design with eligible individuals entering the cohort at the latest of: 1) when they are aged 18 or over; 2) when they have at least a one-year history of CPRD; and 3) 1 January 2004. Censor date was earliest of: 1) registration end date; 2) CPRD-derived date of death; 3) 31 December 2019. Records without a patient acceptability flag for research use were excluded. The sample selection methods are described in more detail in our previous publication.[4]

For the survival analysis models, included individuals (n = 762,803) were aged 18 or over, were born between 1919 and 1986, and had at least one year of registration at a GP practice contributing to CPRD between 2004 – 2019. Individuals without recorded IMD or geographical region, with the gender variable recorded as indeterminate, or who had less than 1 day of follow-up, were excluded (see Figure C). The descriptive statistics for the study sample used for this study are displayed in Table B; a total of 762,803 individuals were included.

We used this sample of primary care records to identify cases of 211 chronic conditions (mentioned above and described in previous work),[15] from which we classified individuals into four states of multimorbidity accumulation: 1) healthy, 2) one chronic condition, 3) basic multimorbidity (2 or more chronic conditions), and 4) complex multimorbidity (3 or more conditions across 3 or more body systems). For each state assumed date of first recording to be date of onset. We assumed that all conditions were life-long (no recovery).

Figure D - Flowchart of inclusion and exclusion criteria for each part of analysis

37

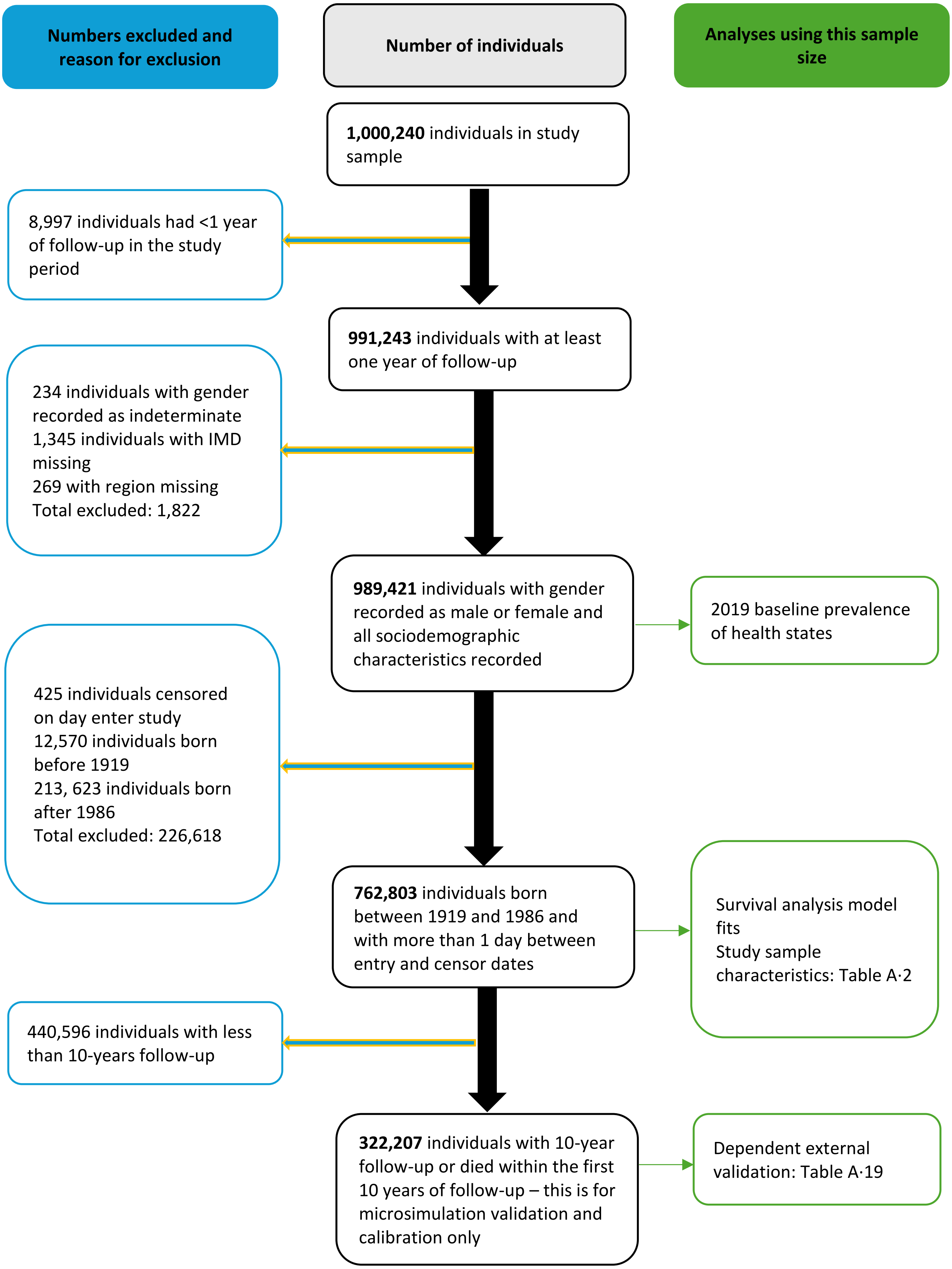

Table A - Baseline sociodemographic characteristics overall for individuals included in the survival analysis models

		N	%
**Overall**		762,803	
**Sex**	Male	378,000	49.6
	Female	384,803	50.4
**Age in years**[×]**†**		41	31,56
**IMD quintile**	1 (least deprived)	164,655	21.6
	2	155,325	20.4
	3	150,557	19.7
	4	153,590	20.1
	5 (most deprived)	138,676	18.2
**Birth cohort**	<1930	42,024	5.5
	1930-1934	26,331	3.5
	1935-1939	30,286	4
	1940-1944	35,818	4.7
	1945-1949	46,984	6.2
	1950-1954	47,147	6.2
	1955-1959	54,325	7.1
	1960-1964	68,239	8.9
	1965-1969	78,357	10.3
	1970-1974	85,348	11.2
	1975-1979	93,648	12.3
	1980-1984	110,009	14.4
	1985-1989	44,287	5.8
**Region**	London	150,411	19.7
	South West	102,825	13.5
	South Central	92,440	12.1
	South East Coast	58,601	7.7
	West Midlands	122,938	16.1
	East Midlands	20,224	2.7
	East of England	41,473	5.4
	North West	118,045	15.5
	Yorkshire and the Humber	28,411	3.7
	North East	27,435	3.6

† Median; interquartile range (IQR) [×] Age is age at entry into the study

IMD = Index of multiple deprivation

Table B Conditions identified by one diagnostic code ever recorded (onset is the date of the first record)

Body system	Conditions		
**Cancer**	Hodgkin Lymphoma	Primary Malignancy - Liver	Primary Malignancy - Thyroid
	Leukaemia	Primary Malignancy - Lung	Primary Malignancy - Uterus
	Myelodysplastic Syndrome	Primary Malignancy - Melanoma	Secondary Malignancy - Adrenal Gland
	Non-Hodgkin Lymphoma	Primary Malignancy - Mesothelioma	Secondary Malignancy - Bone
	Plasma Cell Malignancy	Primary Malignancy - Multiple Sites	Secondary Malignancy - Bowel
	Polycythaemia vera	Primary Malignancy - Oesophageal	Secondary Malignancy - Brain
	Primary Malignancy - Biliary Tract	Primary Malignancy - Oropharyngeal	Secondary Malignancy - Liver
	Primary Malignancy - Bladder	Primary Malignancy - other	Secondary Malignancy - Lung
	Primary Malignancy - Bone	Primary Malignancy - Ovary	Secondary Malignancy - Lymph Nodes
	Primary Malignancy - Bowel	Primary Malignancy - Pancreas	Secondary Malignancy - other
	Primary Malignancy - Brain	Primary Malignancy - Prostate	Secondary Malignancy - Peritoneum
	Primary Malignancy - Breast	Primary Malignancy - Skin	Secondary Malignancy - Pleura
	Primary Malignancy - Cervix	Primary Malignancy - Stomach	
	Primary Malignancy - Kidney	Primary Malignancy - Testis	
**Diseases of the Circulatory System**	Abdominal Aortic Aneurysm	Left bundle branch block	Secondary pulmonary hypertension
	Atrial Fibrillation	Multiple valve disorder	Sick sinus syndrome
	Atrioventricular blocks	Myocardial Infarction	Stable Angina
	Cardiomyopathy – other	Nonrheumatic aortic valve disorders	Stroke - not otherwise specified
	Coronary Heart Disease (not otherwise specified)	Nonrheumatic mitral valve disorders	Subarachnoid haemorrhage
	Dilated cardiomyopathy	Peripheral Arterial Disease	Supraventricular tachycardia
	Heart failure	Primary pulmonary hypertension	Transient ischaemic attack
	Hypertrophic Cardiomyopathy	Raynaud's syndrome	Unstable Angina
	Intracerebral haemorrhage	Rheumatic Valve Disorder	Venous thromboembolic disease (Excl. Pulmonary Embolism)
	Ischaemic stroke	Right bundle branch block combinations	Ventricular tachycardia
**Diseases of the Digestive System**	Alcoholic liver disease	Coeliac disease	Hepatic failure
	Angiodysplasia of colon	Crohn's disease	Irritable bowel syndrome
	Autoimmune liver disease	Diverticular Disease	Oesophageal varices
	Barrett's oesophagus	Fatty Liver	Portal hypertension

	Cirrhosis	Gastro-oesophageal reflux disease	Ulcerative colitis
**Diseases of the Ear**	Hearing loss	Meniere's Disease	
**Diseases of the Endocrine System**	Cystic Fibrosis	Thyroid Disease	Diabetes Mellitus - other or not specified
	Hyperparathyroidism	Type 1 Diabetes Mellitus	
	Polycystic ovarian syndrome	Type 2 Diabetes Mellitus	
**Diseases of the Eye**	Cataract	Macular degeneration	Visual impairment and blindness
	Diabetic Eye Disease	Retinal vascular occlusions	
	Glaucoma	Scleritis and episcleritis	
**Diseases of the Genitourinary System**	Benign Prostatic Hyperplasia	Endometrial hyperplasia and hypertrophy	Obstructive and reflux uropathy
	Chronic Cystitis	Endometriosis	Tubulo-interstitial nephritis
	Chronic Kidney Disease*	Glomerulonephritis	Urinary Incontinence
	Dysmenorrhoea	Neuropathic Bladder	
**Diseases of the Respiratory System**	Asbestosis	Chronic Obstructive Pulmonary Disorder	Pulmonary Fibrosis
	Bronchiectasis	Hypertrophic Nasal Turbinates	Sleep apnoea
	Chronic sinusitis+	Pleural plaque	
**Haematological/ Immunological conditions**	Anaemia -other	Immunodeficiencies	Secondary Thrombocytopaenia
	Aplastic anaemias	Other haemolytic anaemias	Sickle Cell Disease
	Folate deficiency anaemia	Primary Thrombocytopaenia	Thalassaemia
	Hypersplenism	Sarcoidosis	Thrombophilia
	Hyposplenism	Secondary polycythaemia	
**Infectious Diseases**	Chronic viral hepatitis	Human immunodeficiency virus	
**Mental Health Disorders**	Alcohol Misuse	Hyperkinetic disorders	Schizophrenia
	Autism and Asperger's syndrome	Intellectual disability	Substance Misuse
	Dementia	Obsessive-compulsive disorder	
	Eating Disorders	Personality disorders	
**Musculoskeletal conditions**	Ankylosing spondylitis	Intervertebral disc disorders	Rheumatoid Arthritis
	Collapsed vertebra	Lupus Erythematosus	Scleroderma
	Enteropathic arthropathy	Osteoarthritis (excl. spine)	Sjogren's Syndrome
	Fibromatosis	Osteoporosis	Spinal stenosis
	Giant Cell arteritis	Polymyalgia Rheumatica	Spondylolisthesis
	Gout	Psoriatic Arthritis	Spondylosis
**Neurological conditions**	Autonomic Neuropathy	Epilepsy	Parkinson's disease
	Benign essential tremor	Idiopathic Intracranial Hypertension	Peripheral Neuropathy
	Cerebral Palsy	Motor neuron disease	Trigeminal neuralgia

	Chronic Fatigue Syndrome<br>Diabetic Neuropathy	Multiple sclerosis<br>Myasthenia gravis	
**Perinatal conditions**	Congenital Septal Defect	Down's syndrome	Spina bifida
**Skin conditions**	Alopecia areata		

* estimated Glomerular Filtration Rate /creatinine test values also used, as per CKD-EPI formula[16]
+ Chronic sinusitis diagnosis or 2 sinusitis codes at least 84 days apart

Table C - Conditions identified by three or more diagnostic codes within a 1-year period (onset is the date of the third record within 12 months).

Body system	Conditions		
**Diseases of the Circulatory System**	Hypertension	Pericardial Effusion*	
**Diseases of the Digestive System**	Abdominal Hernia*<br>Diaphragmatic hernia*	Gastritis and duodenitis	Pancreatitis
**Diseases of the Ear**	Tinnitus		
**Diseases of the Endocrine System**	Obesity[#]		
**Diseases of the Eye**	Anterior and Intermediate Uveitis	Posterior Uveitis	
**Diseases of the Respiratory System**	Allergic and chronic rhinitis*	Asthma	Pleural effusion
**Mental Health Disorders**	Bipolar affective disorder and mania	Depression	Anxiety disorders
**Musculoskeletal conditions**	Enthesopathy and synovial disorder		
**Neurological conditions**	Migraine		
**Skin conditions**	Acne<br>Dermatitis<br>Hidradenitis suppurativa	Psoriasis<br>Rosacea<br>Seborrheic dermatitis	Vitiligo

*Chronic exceptions: only 1 record of ‘chronic xxx’ is required.
[#] Body mass index/weight/height values also used (see below)

Table D- Conditions identified by two (or more) ‘abnormal’ test results ever recorded (onset is the date of the second ‘abnormal’ result)

Body system	Conditions		
**Diseases of the Endocrine System**	Obesity	Raised low-density lipoprotein cholesterol	Raised Triglycerides
	Low high-density lipoprotein cholesterol	Raised Total Cholesterol	

Distribution of transition times

The transition time inputs into the model were derived from analysis of the CPRD Aurum data as described in Section 1.3. This section describes the survival analysis methods, the fitted models, and the creation of the distribution of transition times.

Parametric survival analysis - methods

In order to get estimates of transition times between states based on individuals' sociodemographic characteristics (age, 5-year birth cohort, sex, IMD quintile, and region), we fitted parametric survival analysis models to all seven transitions defined in the multi-state model structure (Figure A).

For each individual, data was organised into long format with one row per possible transition, with age at entry into the state, age at exit from the state, and a binary status variable for whether the transition in question occurred. This 'counting process' format can account for left-truncation, and the fact that individuals do not all start each state at the same time or age. Figure D shows an example trajectory and data formatted for the counting process.

Recorded dates of onset of each state were used as exact dates of transition between states. Given that a recording of diagnosis of a condition is a proxy marker of a condition's onset, simultaneous diagnosis of multiple conditions may not denote simultaneous onset. For model simplicity, it was assumed that all living states were passed through sequentially, and therefore interim states could not be skipped. In cases where onset of two states were recorded simultaneously, the date of onset of the second state (as defined in Figure A) was delayed by one day. For example, a healthy individual who had diagnosis of two conditions recorded on 1 April 2015 would transition from healthy to incident condition (T1) on 1 April 2015, and then from incident condition to basic multimorbidity (T3) on 2 April 2015 (Figure D).

Parametric models were fitted using the *flexsurvreg* function from the *flexsurv* R package[9]. The distribution for the parametric model makes assumptions as to the shape of the underlying hazard function. For each transition, we selected the distribution for the parametric model was chosen by plotting a non-parametric estimate of the baseline hazard function alongside fitted curves from intercept-only parametric models with each of the possible distributions (Exponential, Weibull (AFT), Gompertz, Gamma, Lognormal, Log-logistic, Generalised gamma)[17,18]. The distribution was then chosen based on a combination of the lowest Akaike's Information Criteria (AIC) value and a visual check of the fitted curve plots.

Figure E - Illustrative example of two hypothetical individuals

**Patient Id**	**1**
**Year of birth**	1970
**Date of entry into study**	1 January 2004
**Age at entry into the study**	33.5
**Health state at entry into study population**	Healthy (no conditions of interest previously recorded)
**Death date**	Not recorded
**Registration end date**	1 January 2016

Conditions recorded during study period:

Date	Condition
**20/02/2010**	Type 2 Diabetes Mellitus
**20/02/2010**	Alcohol misuse

Trajectory:
Calendar time: 01/01/2004 → 20/02/2010 → 21/02/2010 → 01/01/2016
Age: 35.5 → 39.642 → 39.645 → 45.5
State: Healthy 1 condition BMM (Censored)

Trajectory in counting process format*

ID#	Transition	Start time*	Stop time*	Status
**1**	1: Healthy -> 1 condition	17.5	21.642	1
**1**	2: Healthy -> death	17.5	21.642	0
**1**	3: 1 condition -> BMM	21.642	21.645	1
**1**	4: 1 condition -> death	21.642	21.645	0
**1**	5: BMM -> CMM	21.645	27.5	0
**1**	6: BMM-> death	21.645	27.5	0

**Patient Id**	**2**
**Year of birth**	1940
**Date of entry into study**	3 March 2010
**Age at entry into the study**	69.7
**Health state at entry into study population**	1 condition (epilepsy recorded before 2010)
**Death date**	6 October 2018
**Registration end date**	6 October 2018

Conditions recorded during study period:

Date	Condition
**15/07/2014**	Hypertension
**28/07/2015**	Atrial Fibrillation
**03/05/2016**	Chronic Obstructive Pulmonary Disease

Trajectory:
Calendar time: 03/30/2010 → 15/07/2014 → 03/05/2016 → 06/10/2018
Age: 69.7 → 74.0 → 75.8 → 78.3
State: 1 condition BMM CMM Death

Trajectory in counting process format*

ID#	Transition	Start time*	Stop time*	Status
**2**	3: 1 condition -> BMM[+]	51.7	56.0	1
**2**	4: 1 condition -> death[+]	51.7	56.0	0
**2**	5: BMM -> CMM	56.0	57.8	1
**2**	6: BMM-> death	56.0	57.8	0
**2**	7: CMM -> death	57.8	60.3	1

*The time frame is age in years, but with age 18 set as time 0. Status = 1 indicates that this transition occurred
BMM: basic multimorbidity CMM: complex multimorbidity

Parametric models were fitted using the *flexsurvreg* function from the *flexsurv* R package[9]. The distribution for the parametric model makes assumptions as to the shape of the underlying hazard function. For each transition, we selected the distribution for the parametric model was chosen by plotting a non-parametric estimate of the baseline hazard function alongside fitted curves from intercept-only parametric models with each of the possible distributions (Exponential, Weibull (AFT), Gompertz, Gamma, Lognormal, Log-logistic, Generalised gamma)[17,18]. The distribution was then chosen based on a combination of the lowest Akaike's Information Criteria (AIC) value and a visual check of the fitted curve plots. We also assessed Bayesian Information Criteria as a sensitivity analysis.

Age in years was used as the time frame. In order to estimate exact age, needed for the age as a time frame, all individuals were assumed to be born on 1 July of the recorded year of birth. As the focus of this study is accumulation in adults, the age time frame was re-calibrated with age 18 as time 0: 18 was subtracted from all ages at transition. This was to enable fitting the survival analysis models. We adjusted all models for birth cohort effects (represented by 5-year birth-cohort groups) to allow for a more flexible relationship between age and the outcome, whilst also taking into account age as well as left-truncation, and calendar time and cohort effects[19].

In addition to birth cohort and the primary exposure of interest – IMD quintile as a proxy of socioeconomic status, models were also adjusted for sex and geographical region – the other two sociodemographic variables available in the data. Covariates were also considered for inclusion as ancillary parameters (e.g. shape for the Gompertz distribution; Sigma and Q for the generalised gamma), in addition to the location parameter. Models were compared using Akaike's Information Criteria (AIC), and the model with the lowest AIC was chosen for each transition. Model fits were also compared visually to Kaplan-Meier curves.

In brief, a generalised gamma distribution was used to model the time between living states (healthy → 1 condition; 1 condition → basic multimorbidity; basic multimorbidity → complex multimorbidity), and a Gompertz distribution for all transitions to death. Table E summarises the formulae and covariate parameters of the parametric survival analysis models for each of the seven transitions, along with the total numbers of events and individuals at risk. Simultaneous dates of onset for two or more states occurred 49,084 times: 9.1% of all transitions. Further details on model selection and final model coefficients are provided below.

Table E - Summary of parametric distributions and parameters for each transition between states

Transition	Parametric Distribution	Main parameters	Ancillary parameters	Number of events	Number at risk (persons)
**T1 Healthy -> incident condition**	Generalised gamma	IMD quintile, sex, region, 5-year birth cohort	Sigma: sex, region, IMD quintile<br>Q: sex, region, IMD quintile	142,438	401,706
**T2 Healthy -> death**	Gompertz	IMD quintile, sex, region, 5-year birth cohort	Shape: sex, region, IMD	2,460	401,706
**T3 Incident condition -> basic multimorbidity**	Generalised gamma	IMD quintile, sex, region, 5-year birth cohort	Sigma: sex, region, IMD<br>Q: sex, region, IMD quintile	176,459	292,011
**T4 Incident condition -> death**	Gompertz	IMD quintile, sex, region, 5-year birth cohort	Shape: sex, region, IMD quintile	2,970	292,011
**T5 Basic multimorbidity -> complex multimorbidity**	Generalised gamma	IMD quintile, sex, region, 5-year birth cohort	(None)	155,621	287,967
**T6 Basic multimorbidity -> death**	Gompertz	IMD quintile, sex, region, 5-year birth cohort	Shape: sex, IMD quintile	7,272	287,967
**T7 Complex multimorbidity -> death**	Gompertz	IMD quintile, sex, region, 5-year birth cohort	Shape: sex, IMD quintile	48,839	255,637

IMD = Index of Multiple Deprivation T# = Transition number

Parametric survival analysis – choice of parametric model distributions

Non-parametric hazard estimates for each transition were plotted alongside intercept-only models for selected probability distributions commonly used in parametric survival analysis: Exponential, Weibull, Gompertz, Gamma, Log-normal, Log-logistic and Generalised Gamma. These plots are presented below, alongside the Akaike Information Criteria values for each of the parametric models. We used the *bshazards* package in R to estimate the hazard function[18]. This package, which uses b-splines and generalised linear mixed models, allows data to be left-truncated and right-censored, and is therefore preferable to other methods such as plotting the kernel density estimates. **Please note that in all plots, year 0 represents age 18.**

Transition 1: Healthy → initial condition

Figure F – Non-parametric and parametric estimates of the hazard function for transition 1: healthy -> 1 condition

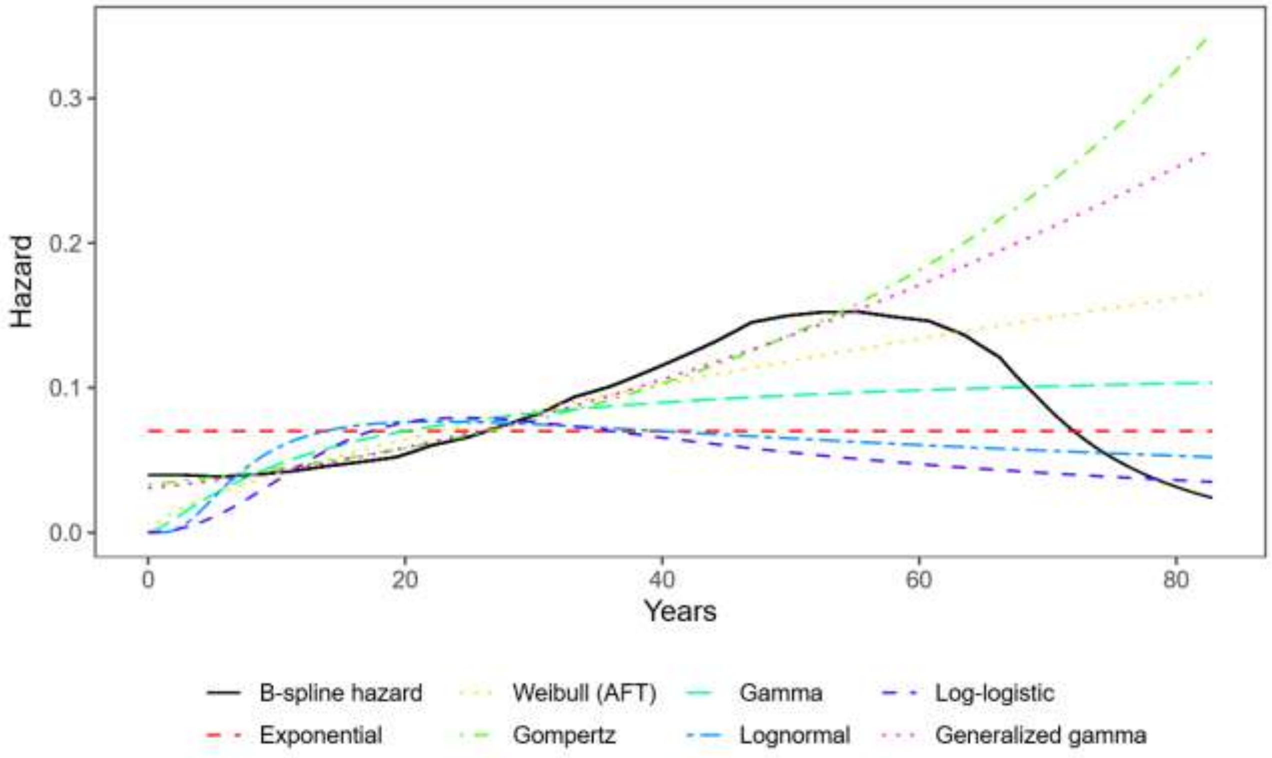

* Time is years since age 18. Time 0 = age 18; AFT = Accelerated failure time

Table F - Akaike's Information Criteria (AIC) and Bayesian Information Criteria (BIC) values for intercept-only parametric models for transition 1: healthy -> 1 condition

**Distribution**	**Generalised Gamma**	**Gompertz**	**Weibull**	**Gamma**	**Exponential**	**Log-normal**	**Log-logistic**
**AIC**	1,018,580	101,9278	1,021,418	1,027,755	1,041,816	1,051,404	1,055,209
**BIC**	1,018,612	1,019,300	1,021,440	1,027,777	1,041,827	1,051,425	1,055,231

AIC = Akaike's Information Criteria; BIC = Bayesian Information Criteria

Transition 2: Healthy → Death

Figure G - Non-parametric and parametric estimates of the hazard function for transition 2: healthy -> death

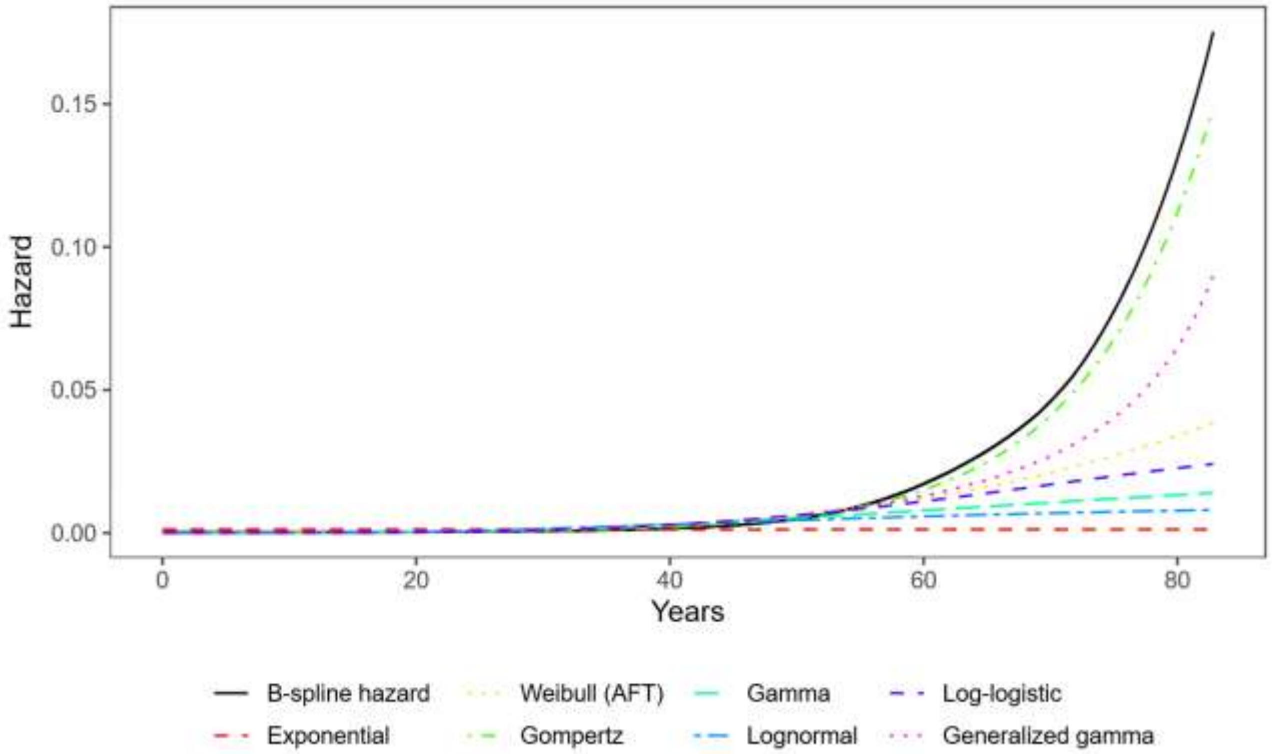

* Time is years since age 18. Time 0 = age 18; AFT = Accelerated failure time

Table G - Akaike's Information Criteria (AIC) and Bayesian Information Criteria (BIC) values for intercept-only parametric models for transition 2: healthy -> death

Distribution	Generalised Gamma	Gompertz	Weibull	Log-logistic	Gamma	Log-normal	Exponential
**AIC**	31,259	31,591	32,703	32,895	33,535	34,434	37,964
**BIC**	31,291	31,613	32,725	32,917	33,557	34,456	37,975

AIC = Akaike’s Information Criteria; BIC = Bayesian Information Criteria

Transition 3: Initial condition → Basic multimorbidity

Figure H - Non-parametric and parametric estimates of the hazard function for transition 2: 1 condition -> basic multimorbidity

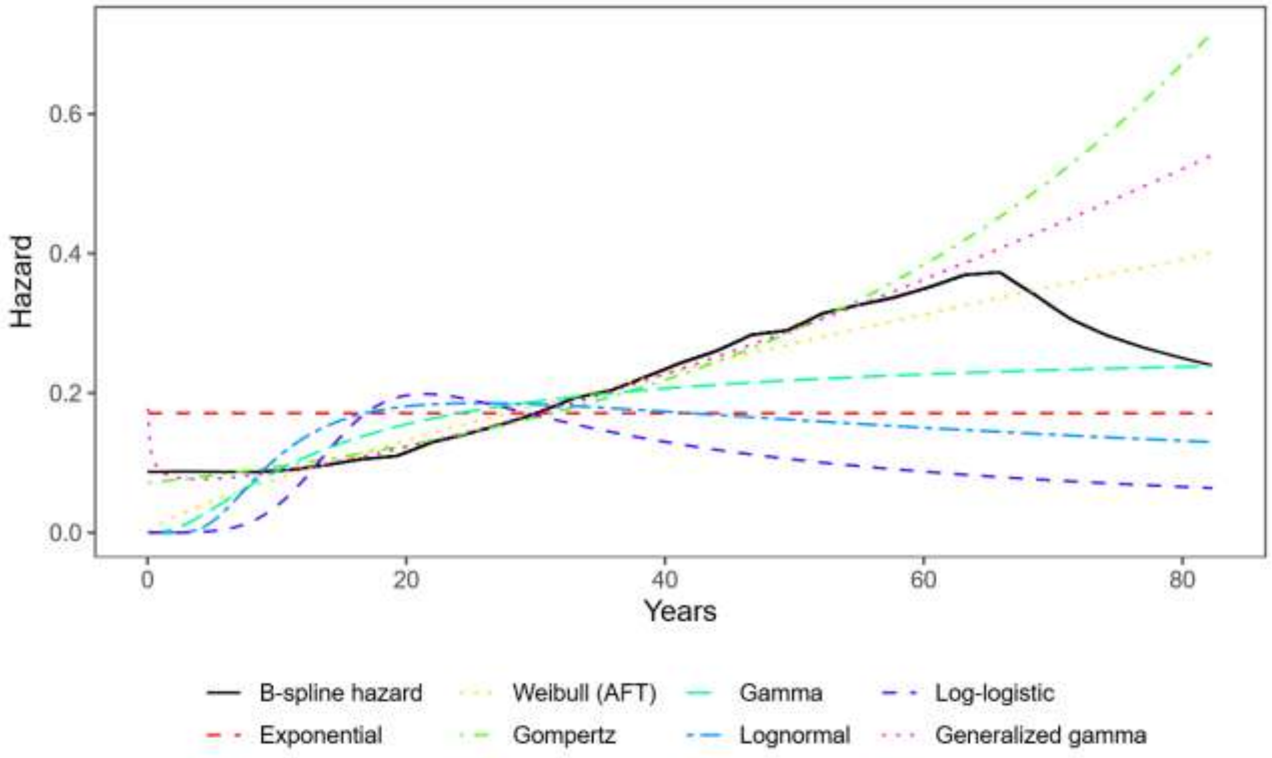

* Time is years since age 18. Time 0 = age 18; AFT = Accelerated failure time

Table H - Akaike's Information Criteria (AIC) and Bayesian Information Criteria (BIC) values for intercept-only parametric models for transition 3: 1 condition -> basic multimorbidity

Distribution	Generalised Gamma	Gompertz	Weibull	Gamma	Exponential	Log-normal	Log-logistic
**AIC**	947,128	947,777	949,511	959,151	975,900	988,054	1,029,179
**BIC**	947159	947798	949532	959172	975910	988075	1029200

AIC = Akaike’s Information Criteria; BIC = Bayesian Information Criteria

Transition 4: Initial condition → Death

Figure I - Non-parametric and parametric estimates of the hazard function for transition 4: healthy -> death

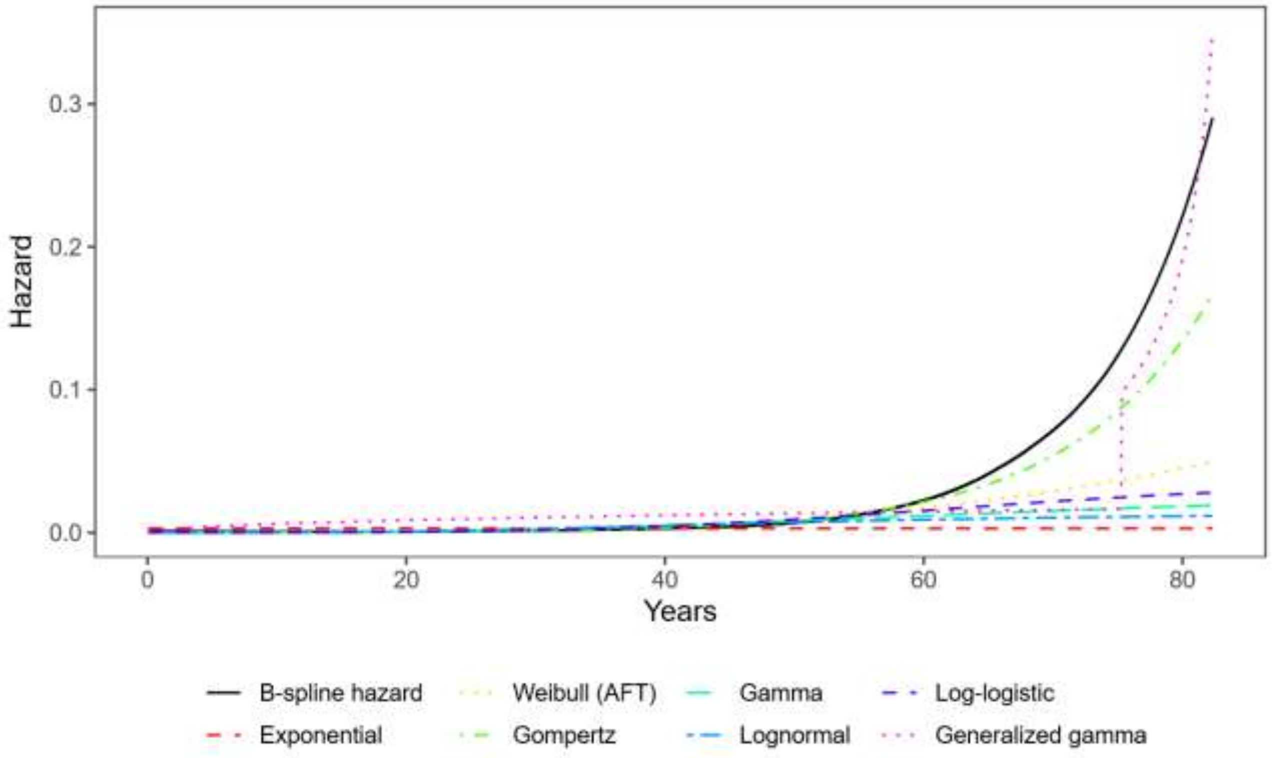

* Time is years since age 18. Time 0 = age 18; AFT = Accelerated failure time

Table I - Akaike's Information Criteria (AIC) and Bayesian Information Criteria (BIC) values for intercept-only parametric models for transition 4: 1 condition -> death

Distribution	Generalised Gamma	Gompertz	Weibull	Log-logistic	Gamma	Log-normal	Exponential
**AIC**	26,652	35,242	36,204	36,478	37,016	37,867	40,690
**BIC**	26,683	35,263	36,226	36,499	37,037	37,888	40,700

AIC = Akaike's Information Criteria; BIC = Bayesian Information Criteria

Transition 5: Basic multimorbidity → Complex multimorbidity

Figure J - Non-parametric and parametric estimates of the hazard function for transition 5: basic multimorbidity -> complex multimorbidity

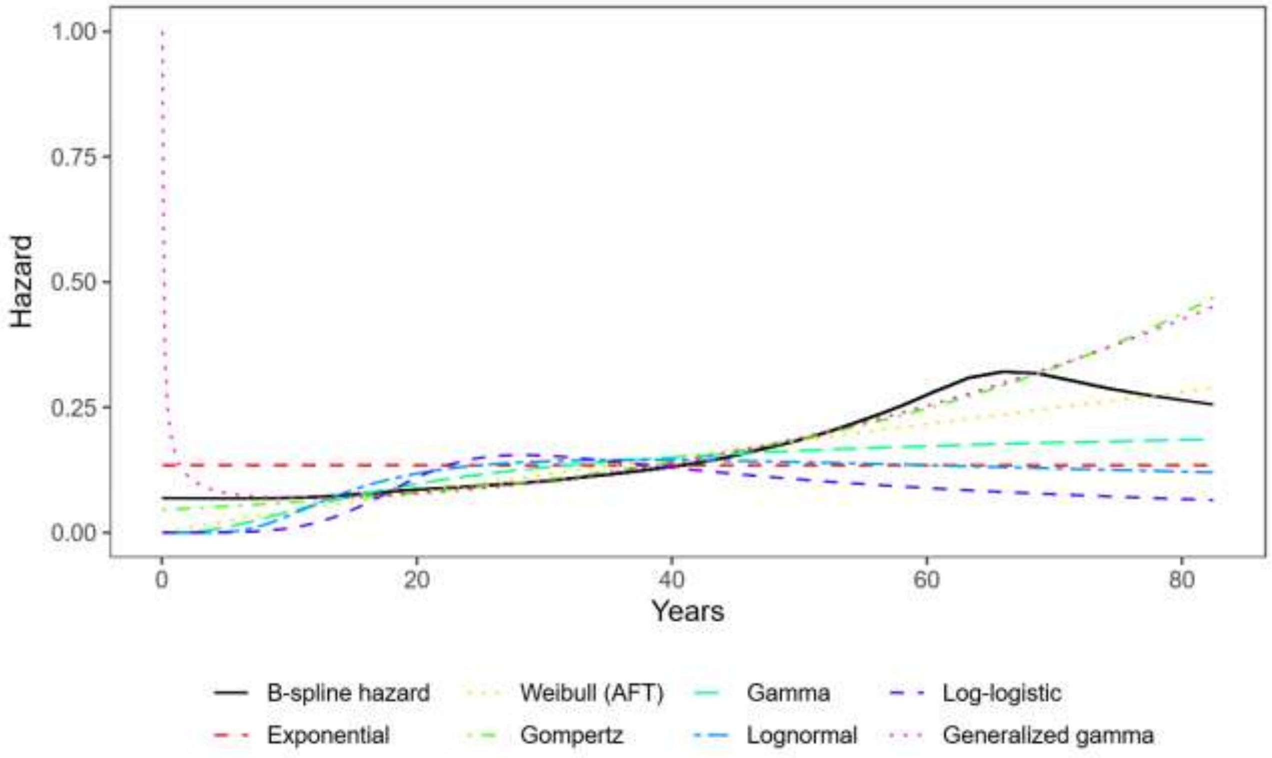

* Time is years since age 18. Time 0 = age 18; AFT = Accelerated failure time

Table J - Akaike's Information Criteria (AIC) and Bayesian Information Criteria (BIC) values for intercept-only parametric models for transition 5: basic multimorbidity -> complex multimorbidity

Distribution	Generalised Gamma	Gompertz	Weibull	Gamma	Exponential	Log-normal	Log-logistic
**AIC**	911,475	911,598	915,024	922,484	935,823	939,529	968,466
**BIC**	911,506	911,619	915,045	922,506	935,834	939,550	968,487

AIC = Akaike's Information Criteria; BIC = Bayesian Information Criteria

Transition 6: Basic multimorbidity → Death

Figure K - Non-parametric and parametric estimates of the hazard function for transition 6: basic multimorbidity -> death

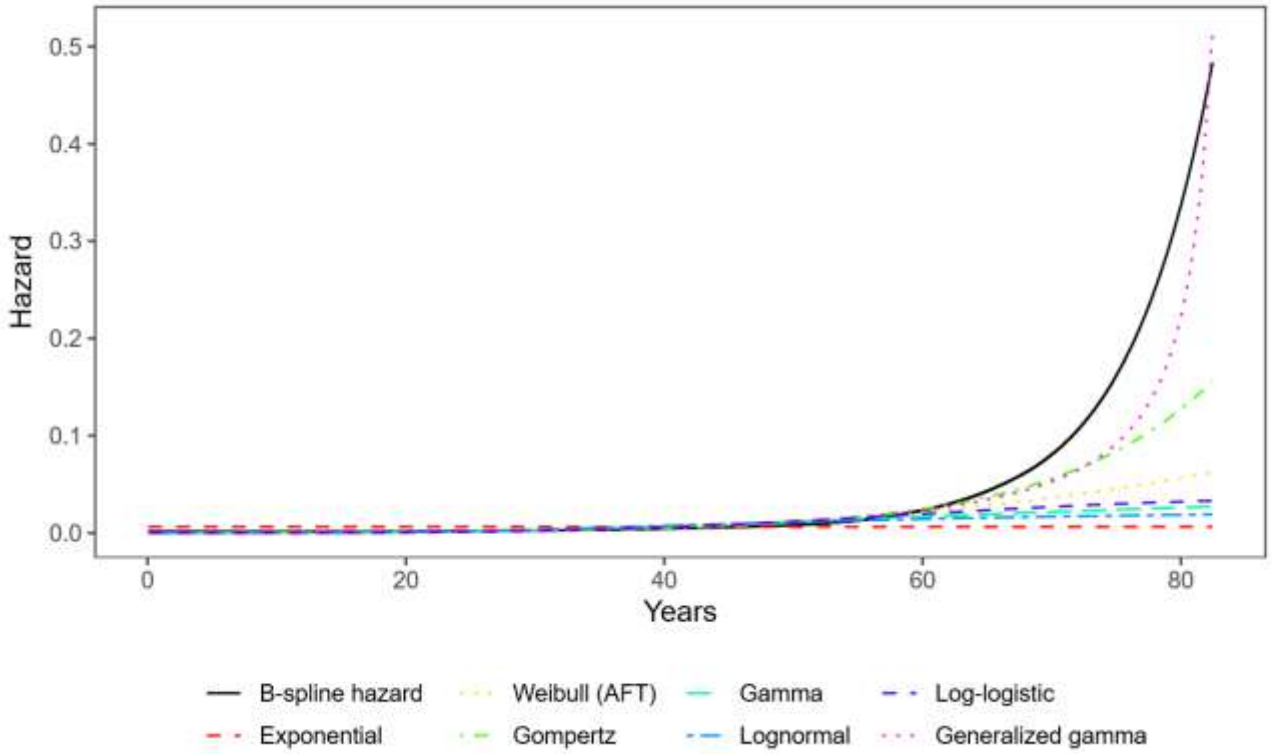

* Time is years since age 18. Time 0 = age 18; AFT = Accelerated failure time

Table K - Akaike's Information Criteria (AIC) and Bayesian Information Criteria (BIC) values for intercept-only parametric models for transition 6: basic multimorbidity -> death

Distribution	Gompertz	Generalised Gamma	Weibull	Log-logistic	Gamma	Log-normal	Exponential
**AIC**	79,487	80,046	80,815	81,521	82,221	83,375	88,286
**BIC**	79,508	80,077	80,836	81,542	82,242	83,396	88,296

AIC = Akaike’s Information Criteria; BIC = Bayesian Information Criteria

Transition 7: Complex multimorbidity → Death

Figure L - Non-parametric and parametric estimates of the hazard function for transition 7: complex multimorbidity -> death

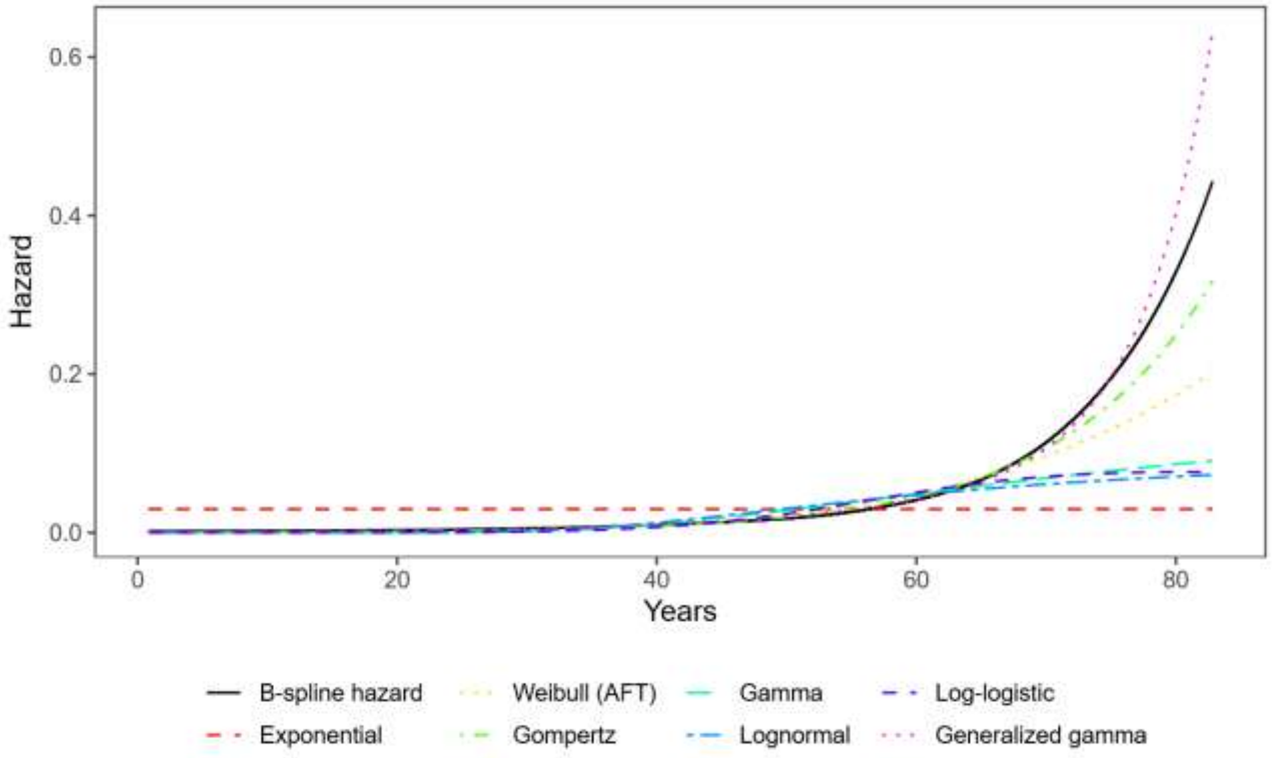

* Time is years since age 18. Time 0 = age 18; AFT = Accelerated failure time

Table L - Akaike's Information Criteria (AIC) and Bayesian Information Criteria (BIC) values for intercept-only parametric models for transition 7: complex multimorbidity -> death

**Distribution**	**Gompertz**	**Generalised Gamma**	**Weibull**	**Log-logistic**	**Gamma**	**Log-normal**	**Exponential**
**AIC**	79,487	80,046	80,815	81,521	82,221	83,375	88,286
**BIC**	79,508	80,077	80,836	81,542	82,242	83,396	88,296

AIC = Akaike’s Information Criteria; BIC = Bayesian Information Criteria

Parametric survival analysis – model fits and interpretation of coefficients

As the interpretations of the proportional hazard (PH) and acceleration failure time (AFT) parametric models differ, we have presented model results from the generalised gamma (AFT) and Gompertz (PH) distributions separately.

The use of ancillary parameters for modelling the survival distributions for each transition for both Gompertz and generalised gamma parametric models can make the model outputs difficult to interpret without application to specific covariate values. For the transitions modelled as accelerated failure time with the generalised gamma distribution, the exponent of the coefficient gives the acceleration factor, which describes the effect of the predictor on survival time. Acceleration factors of less than one indicate shorter time to event, whilst exposure with acceleration factors greater than one can be interpreted as increasing time to event. The

acceleration factors for the generalised gamma parametric models for transitions between living states are presented in Table M. Where covariates are included as ancillary parameters sigma and Q, these ancillary parameters change the effect of the covariate over time.

For the transitions modelled with the Gompertz distribution, the exponent of the coefficient gives the hazard ratio (HR): the effect of the covariate on the instantaneous hazard in comparison to the reference groups. A hazard ratio greater than 1 describes increased risk of an event at time *t*, whilst a hazard ratio less than 1 represents decreased risk of an event. Where a covariate is included as an ancillary shape parameter, the effect of the covariate changes over time: if the hazard ratio at age 18 (time 0) is > 1 and the shape is > 1 then the effect of the covariate increases over time. The hazard ratio for the Gompertz parametric models for transitions from living states to death are presented in Table N.

Parametric survival analysis – distribution of transition times

We used the fitted survival analysis models to generate the look-up table of the distribution of time to event for each transition for all combinations of covariates (sex, IMD quintile, geographic region, and 5-year birth cohort) and for each year of age at entry (ages 30-90). The *predict*() function from the *flexsurv* survival package was used to predict the time to each transition at quantile intervals of 0.1 (deciles), and conditional on survival to each year of age[9]. The maximum predicted time was capped so that transitions did not occur after age 110; this is the maximum age used by the Office for National Statistics (ONS) life expectancy calculations[10].

Section 1.6 describes how the distribution of transition times were altered under each of the five scenarios modelled in this paper.

Table M- Acceleration factor (AF) for transitions between alive states (parametric models with generalised gamma distribution)

Acceleration factor (AF) values <1 mean shorter transition times (i.e. faster transition/higher risk). Estimates are presented alongside 95% confidence intervals.

Parameter	Variable	Level	Transition 1: Healthy -> Initial condition			Transition 3: Initial condition -> BMM			Transition 5: BMM -> CMM		
			**AF**	LCI	UCI	**AF**	LCI	UCI	**AF**	LCI	UCI
	Sex	Male	**1**	1	1	**1**	1	1	**1**	1	1
		Female	**0·73**	0·72	0·74	**0·81**	0·78	0·83	**0·98**	0·97	0·98
	IMD quintile	1 (least deprived)	**1**	1	1						
		2	**0·98**	0·96	0·99	**0·94**	0·91	0·97	**0·98**	0·97	0·99
		3	**0·96**	0·94	0·98	**0·92**	0·89	0·95	**0·96**	0·95	0·97
		4	**0·94**	0·92	0·96	**0·88**	0·85	0·91	**0·94**	0·93	0·95
		5 (most deprived)	**0·89**	0·87	0·91	**0·81**	0·78	0·84	**0·90**	0·89	0·91
	Region	London	**1**	1	1	**1**	1	1	**1**	1	1
		South West	**0·96**	0·94	0·98	**1·05**	1·01	1·09	**0·96**	0·95	0·97
		South Central	**0·96**	0·94	0·98	**1·03**	0·99	1·07	**0·98**	0·97	0·99
		South East Coast	**0·93**	0·90	0·95	**1·00**	0·95	1·04	**0·99**	0·98	1·01
		West Midlands	**0·87**	0·86	0·89	**0·94**	0·91	0·98	**0·96**	0·95	0·97
		East Midlands	**0·93**	0·89	0·96	**1·03**	0·96	1·09	**0·94**	0·92	0·96
		East of England	**0·89**	0·87	0·92	**0·97**	0·92	1·02	**0·95**	0·93	0·96
		North West	**0·86**	0·84	0·87	**0·94**	0·91	0·98	**0·95**	0·94	0·96
		Yorkshire and the Humber	**0·86**	0·83	0·89	**1·00**	0·95	1·05	**0·93**	0·91	0·94
		North East	**0·84**	0·81	0·86	**0·99**	0·94	1·04	**0·93**	0·91	0·94
	Birth cohort	<1930	**1**	1	1	**1**	1	1	**1**	1	1
		1930-1934	**0·90**	0·88	0·93	**0·98**	0·96	1·00	**1·04**	1·02	1·05
		1935-1939	**0·86**	0·84	0·88	**0·98**	0·96	0·99	**1·11**	1·09	1·13
		1940-1944	**0·84**	0·82	0·85	**0·99**	0·98	1·01	**1·19**	1·17	1·21
		1945-1949	**0·83**	0·82	0·85	**1·01**	0·99	1·03	**1·26**	1·23	1·29
		1950-1954	**0·84**	0·83	0·86	**1·02**	1·00	1·04	**1·31**	1·28	1·35
		1955-1959	**0·86**	0·84	0·88	**1·05**	1·03	1·08	**1·36**	1·32	1·40
		1960-1964	**0·87**	0·85	0·89	**1·09**	1·06	1·11	**1·37**	1·33	1·42

		1965-1969	**0·88**	0·85	0·90	**1·11**	1·08	1·14	**1·36**	1·31	1·41
		1970-1974	**0·87**	0·85	0·90	**1·14**	1·10	1·17	**1·34**	1·29	1·40
		1975-1979	**0·88**	0·85	0·91	**1·16**	1·12	1·20	**1·20**	1·24	1·35
		1980-1984	**0·86**	0·84	0·89	**1·17**	1·13	1·22	**1·20**	1·15	1·26
		1985-1989	**0·81**	0·79	0·85	**1·14**	1·09	1·18	**1·10**	1·05	1·16
**Sigma**	Sex	Male	**1**	1	1	**1**	1	1			
		Female	**1·40**	1·38	1·42	**1·44**	1·38	1·49			
	IMD quintile	1 (least deprived)	**1**	1	1	**1**	1	1			
		2	**1·01**	0·99	1·03	**1·03**	1·00	1·07			
		3	**1·02**	1·00	1·04	**1·01**	0·97	1·05			
		4	**1·03**	1·01	1·06	**1·00**	0·96	1·03			
		5 (most deprived)	**1·08**	1·06	1·10	**0·99**	0·95	1·03			
	Region	London	**1**	1	1	**1**	1	1			
		South West	**1·10**	1·07	1·12	**1·08**	1·04	1·13			
		South Central	**1·04**	1·01	1·06	**1·10**	1·05	1·16			
		South East Coast	**1·09**	1·06	1·12	**1·06**	1·01	1·12			
		West Midlands	**1·09**	1·06	1·11	**1·11**	1·07	1·16			
		East Midlands	**1·02**	0·99	1·06	**1·05**	0·98	1·13			
		East of England	**1·09**	1·05	1·13	**1·10**	1·04	1·17			
		North West	**1·13**	1·11	1·16	**1·06**	1·02	1·11			
		Yorkshire and the Humber	**1·10**	1·06	1·14	**1·08**	1·02	1·15			
		North East	**1·08**	1·04	1·12	**1·09**	1·03	1·16			
**Q**	Sex	Male	**1**	1	1	**1**	1	1			
		Female	**1·42**	1·34	1·51	**2·10**	1·91	2·31			
	IMD quintile	1 (least deprived)	**1**	1	1	**1**	1	1			
		2	**1·05**	0·96	1·14	**1·01**	0·93	1·10			
		3	**1·00**	0·92	1·08	**0·90**	0·83	0·98			
		4	**0·97**	0·90	1·06	**0·85**	0·78	0·92			
		5 (most deprived)	**0·89**	0·82	0·97	**0·75**	0·69	0·82			
	Region	London	**1**	1	1	**1**	1	1			
		South West	**1·14**	1·03	1·26	**1·11**	1·01	1·23			
		South Central	**1·23**	1·11	1·36	**1·19**	1·06	1·32			
		South East Coast	**1·04**	0·93	1·16	**1·05**	0·94	1·18			

	West Midlands	**1·08**	0·99	1·18	**1·18**	1·07	1·285	
	East Midlands	**1·08**	0·93	1·26	**1·13**	0·94	1·352	
	East of England	**1·15**	1·00	1·32	**1·13**	0·99	1·292	
	North West	**0·86**	0·79	0·94	**1·04**	0·96	1·138	
	Yorkshire and the Humber	**1·24**	1·08	1·44	**1·15**	0·99	1·321	
	North East	**1·19**	1·03	1·37	**1·27**	1·10	1·459	
**AIC**		**1,011,906**			**944,480**			**909,031**
**BIC**		**1,040,507**			**946,064**			**887,303**

AIC = Akaike Information Criteria BIC = Bayesian Information Criteria AF = Acceleration factor IMD = Index of Multiple Deprivation
LCI = lower 95% confidence interval UCI = upper 95% confidence interval

Table N - Hazard ratios (HR) for transitions between alive states (parametric models with Gompertz distribution)

Hazard ratio (HR) values >1 mean shorter transition times (i.e. faster transition/higher risk). Estimates are presented alongside 95% confidence intervals.

**Parameter**	**Variable**	**Level**	**Transition 2: Healthy -> Death**			**Transition 4: First condition -> Death**			**Transition 6: BMM -> Death**			**Transition 7: CMM -> Death**		
			**HR**	LCI	UCI	**HR**	UCI	UCI	**HR**	LCI	UCI	**HR**	LCI	UCI
	Sex	Male	**1**	1	1	**1**	1	1	**1**	1	1	**1**	1	1
		Female	0·37	0·28	0·48	0·37	0·29	0·47	0·41	0·35	0·49	0·41	0·37	0·45
	IMD quintile	1 (least deprived)	**1**	1	1	**1**	1	1	**1**	1	1	**1**	1	1
		2	1·51	0·99	2·30	1·10	0·73	1·66	1·15	0·85	1·54	1·31	1·10	1·56
		3	1·76	1·16	2·67	1·16	0·77	1·75	1·34	1·00	1·79	2·15	1·81	2·55
		4	2·65	1·77	3·97	1·88	1·27	2·78	1·56	1·17	2·08	3·27	2·76	3·87
		5 (most deprived)	3·94	2·66	5·83	3·59	2·48	5·21	2·60	1·97	3·42	4·50	3·82	5·30
	Region	London	**1**	1	1	**1**	1	1	**1**	1	1	**1**	1	1
		South West	1·49	0·96	2·31	0·72	0·63	0·83	0·93	0·85	1·02	1·01	0·97	1·05
		South Central	1·70	1·06	2·71	0·92	0·79	1·06	1·12	1·02	1·24	1·15	1·11	1·20
		South East Coast	1·29	0·75	2·20	0·90	0·76	1·06	1·06	0·96	1·18	1·03	0·99	1·08
		West Midlands	1·48	1·00	2·19	1·00	0·88	1·13	1·03	0·94	1·12	1·05	1·02	1·09
		East Midlands	2·17	1·01	4·65	0·80	0·60	1·07	0·82	0·68	0·98	1·09	1·02	1·17
		East of England	0·59	0·29	1·20	0·93	0·78	1·12	0·95	0·84	1·07	1·04	0·99	1·09
		North West	1·52	1·05	2·20	1·05	0·92	1·19	1·08	0·99	1·17	1·17	1·13	1·21
		Yorkshire & the Humber	1·43	0·72	2·84	0·99	0·81	1·21	1·01	0·89	1·16	1·14	1·09	1·20
		North East	1·39	0·71	2·73	0·95	0·78	1·17	0·96	0·84	1·09	1·13	1·08	1·19
	Birth cohort	<1930	**1**	1	1	**1**	1	1	**1**	1	1	**1**	1	1
		1930-1934	0·81	0·69	0·95	0·64	0·56	0·74	0·57	0·52	0·62	0·80	0·78	0·83
		1935-1939	0·66	0·55	0·79	0·49	0·42	0·58	0·41	0·37	0·45	0·71	0·69	0·73
		1940-1944	0·59	0·48	0·72	0·35	0·29	0·42	0·33	0·29	0·37	0·66	0·63	0·69
		1945-1949	0·37	0·29	0·48	0·28	0·23	0·35	0·27	0·24	0·30	0·66	0·63	0·70
		1950-1954	0·38	0·29	0·50	0·23	0·18	0·29	0·24	0·21	0·28	0·69	0·65	0·74
		1955-1959	0·32	0·23	0·44	0·22	0·16	0·28	0·22	0·18	0·26	0·71	0·66	0·77
		1960-1964	0·25	0·18	0·35	0·18	0·13	0·24	0·19	0·16	0·24	0·74	0·67	0·82

		1965-1969	0·32	0·22	0·46	0·16	0·11	0·22	0·19	0·15	0·23	0·83	0·74	0·93
		1970-1974	0·26	0·17	0·40	0·12	0·08	0·17	0·21	0·16	0·27	0·88	0·76	1·03
		1975-1979	0·36	0·22	0·57	0·12	0·08	0·19	0·18	0·13	0·24	0·94	0·77	1·17
		1980-1984	0·35	0·21	0·59	0·17	0·11	0·28	0·18	0·13	0·26	0·98	0·75	1·29
		1985-1989	0·43	0·24	0·79	0·17	0·10	0·31	0·22	0·14	0·35	1·07	0·64	1·80
**Shape**	Sex	Male	**1**	1	1	1	1	1	1	1	1	1	1	1
		Female	1·01	1·01	1·02	1·01	1·01	1·02	1·01	1·01	1·01	1·01	1·01	1·01
	IMD quintile	1 (least deprived)	**1**	1	1	1	1	1	1	1	1	1	1	1
		2	0·99	0·99	1·00	1·00	0·99	1·01	1·00	1·00	1·01	1·00	1·00	1·00
		3	0·99	0·99	1·00	1·00	1·00	1·01	1·00	1·00	1·01	0·99	0·99	0·99
		4	0·99	0·98	1·00	1·00	0·99	1·01	1·00	0·99	1·00	0·99	0·98	0·99
		5 (most deprived)	0·99	0·98	0·99	0·99	0·98	1·00	0·99	0·99	1·00	0·98	0·98	0·99
	Region	London	**1**	1	1									
		South West	0·99	0·98	1·00									
		South Central	0·99	0·98	1·00									
		South East Coast	1·00	0·99	1·01									
		West Midlands	0·99	0·99	1·00									
		East Midlands	0·98	0·97	1·00									
		East of England	1·01	1·00	1·03									
		North West	1·00	0·99	1·01									
		Yorkshire & the Humber	1·00	0·98	1·01									
		North East	0·99	0·98	1·01									
**AIC**			**31,074**			**34,6612**			**78,245**			**389,523**		
**BIC**			**33,780**			**37,508**			**84,850**			**377,600**		

AIC = Akaike Information Criteria BIC = Bayesian Information Criteria HR = Hazard ratio IMD = Index of Multiple Deprivation
LCI = lower 95% confidence interval UCI = upper 95% confidence interval

Synthetic population

The synthetic population to be simulated in the baseline scenario was created to represent the English adult population structure (ages 30-90) and sociodemographic characteristics in 2019, the final year of the primary care data available in our sample. The mid-year population estimates for 2019 stratified by sex, IMD quintile, single year of age, and region were obtained from the Office for National Statistics (ONS)[20].
Sex, IMD quintile, and region characteristics were assigned at the start of the model, and not updated. We restricted the starting age of simulants to between ages 30-90. The lower limit of age 30 was chosen as the latest 5-year birth cohort included in our parametric survival analysis inputs was 1985-1989. Age 90 was the latest age of entry as this is the oldest age for which ONS produces population estimates[20]. For each year from 2020-2049, a new cohort of 30-year-olds was introduced into the model, based on ONS population projection estimates for 30-year-olds in that year[21]. As ONS population projections are available only by sex and single year of age, we randomly assigned region and IMD quintile to these new cohorts based on the distribution of sex, region, and IMD quintile in 30-year-olds from the 2019 ONS population estimates. To reduce computational requirements, a 1% stratified sample of the population was used, and results were scaled back up after simulation.
The starting health state of simulants was randomly assigned based on weights derived from the proportion of individuals by sex, 5-year age-group and IMD quintile in each state in 2019 in our CPRD Aurum sample.

Synthetic population baseline characteristics

A total of 357,159 adults aged 30-90 were included in the initial synthetic population in 2019 (1% of the ONS England mid-year population estimates for 2019). Table O presents the demographic characteristics of the baseline synthetic population in 2019, along with the proportion starting in each health state for the initial year: healthy, initial chronic condition, basic multimorbidity, and complex multimorbidity. Slightly over one third start in the healthy state, with approximately one fifth starting in each of the initial chronic condition, basic multimorbidity, and complex multimorbidity states.

Table O - Synthetic population characteristics in 2019

	Overall		Initial state (%)			
	N	%	Healthy	1 condition	BMM	CMM
**2019 simulated population**	357,159		29·7	16·6	20·8	33·0
**Sex**						
Male	172,894	48·4	34·5	15·9	19·9	29·6
Female	184,265	51·6	25·1	17·2	21·5	36·2
**Age in years†×**	54	41,67	43(36,53)	47(38,57)	55(45,65)	68(57,77)
**10-year age-groups×**						
30-39	75,418	21·1	56·1	22·8	14·5	6·6
40-49	71,291	20·0	41·7	22·6	21·5	14·2
50-59	75,787	21·2	26·5	18·6	27·2	27·6
60-69	59,092	16·5	14·0	12·4	26·8	46·8
70-79	47,198	13·2	7·4	6·8	18·3	67·5
80+	28,373	7·9	6·8	4·3	9·8	79·1
**IMD quintile**						
1 (least deprived)	73,453	20·6	28·4	16·7	21·1	33·8
2	74,382	20·8	28·4	16·6	21·0	34·0
3	73,647	20·6	29·4	16·6	20·8	33·2
4	70,509	19·7	31·2	16·7	20·7	31·3
5 (most deprived)	65,168	18·2	31·1	16·2	20·2	32·5
**Region**						
London	54,121	15·2	34·3	18·1	20·3	27·2
South West	37,230	10·4	27·5	16·1	20·8	35·5
South Central	28,130	7·9	30·0	17·1	20·8	32·1
South East Coast	31,140	8·7	28·4	16·7	20·9	33·9
West Midlands	36,943	10·3	29·2	16·2	20·9	33·8
East Midlands	30,875	8·6	28·8	16·2	21·3	33·7
East of England	40,554	11·4	29·2	16·4	20·6	33·7
North West	46,440	13	28·8	16·1	20·7	34·4
Yorkshire and the Humber	34,545	9·7	29·3	15·9	20·9	34·0
North East	17,181	4·8	27·8	16·0	20·7	35·5

BMM = Basic multimorbidity CMM = Complex multimorbidity IMD = Index of multiple deprivation
† Median; interquartile range (IQR) × Age is age in 2019

Linear correlation matrix

As people do not live indefinitely, time in each state is not independent of time spent in preceding states. For example, someone who lives for 70 years without any chronic conditions, is unlikely to spend 50 years living with one or more chronic conditions. To account for this, a linear correlation matrix of time in each state (Table P) was calculated from the CPRD Aurum data sample based on past state history.

Table P - Correlation matrix for dependence between transitions

	**T1: Healthy -> incident condition**	**T2: Healthy -> death**	**T3: Incident condition -> basic MM**	**T4: Incident condition -> death**	**T5: Basic MM-> complex MM**	**T6: Basic MM -> death**	**T7: Complex MM -> death**
**T1: Healthy -> incident condition**	1	0	-0.175	-0.150	-0.222	-0.172	-0.306
**T2: Healthy -> death**	0	1	0	0	0	0	0
**T3: Incident condition -> basic multimorbidity**	-0.174	0	1	0	-0.148	-0.095	-0.199
**T4: Incident condition -> death**	-0.150	0	0	1	0	0	0
**T5: Basic multimorbidity -> complex multimorbidity**	-0.222	0	-0.148	0	1	0	-0.222
**T6: Basic multimorbidity -> death**	-0.172	0	-0.095	0	0.000	1	0
**T7: Complex multimorbidity -> death**	-0.306	0	-0.199	0	-0.222	0	1

MM = multimorbidity T# = Transition number

1.6. Time horizon

The time horizon for the microsimulation model is 30 years: from 2019 to 2049. This mid-term timeframe was chosen as a compromise between the longer time frames generally required for public health prevention efforts to take effect, and the inherent limitations in projecting far into the future based on historical data.

1.7. Modelling alternative scenarios

As the microsimulation model uses a lookup table of the distributions for time to each transition (Section 1.3), alternative scenarios can be easily modelled by changing the distributions of the length of time spent in each state for each combination of covariate characteristics.
As the aim of this study is to quantify the impact of potential inequality reduction scenarios, we chose to alter the model inputs only based on the socioeconomic risk factor (quintiles of IMD), and have not made any adjustments based on sex, age, or geographic region. We created the alternative scenarios in two main ways: 1) applying a multiplicative/reduction factor to each of the transition times, either uniformly to all subgroups or dependent on subgroup characteristics; 2) assuming the same time to transition as another sub-group. The latter equates to assuming that someone from IMD quintile X has the same transition times as someone from IMD quintile Y with the same demographic characteristics. To illustrate scenarios 1-4, we set a target of preventing or postponing 3% of projected cumulative deaths in the baseline scenario between 2019-2049. We then iteratively seached for a level of improvement that would result in the target reduction in deaths. We used the 2019 baseline differences in age-sex standardised prevalence of basic multimorbidity

between the IMD quintiles to generate the ratio of improvements between quintiles. For each of the five scenarios, the method of adjustment for each IMD quintile is summarised Table Q. We applied all scenarios to each of the seven transitions between the five states in the model (see Figure A for a diagram of the transitions and Section 1.3 for a summary of the model methods).

Table Q - Summary of scenarios

**Scenario**		**Summary**	**IMD quintile (1 least deprived; 5 most deprived)**				
**#**	name		1	2	3	4	5
**1**	Targeted	Target solely the worst-off groups: Reduce the gap between IMD4&5 and IMD3	As baseline	As baseline	As baseline	Reduce the gap in transition times with IMD3 by 73.87025%	Reduce the gap in transition times with IMD3 by 74.44701%
**2**	Universal with a focus on the gap	Target the whole population, with an additional focus on the most deprived groups	Increase the length of time to transition by 2.79%	Increase the length of time to transition by 2.79%	Increase the length of time to transition by 2.79%	Increase the length of time to transition by 5.379%	Increase the length of time to transition by 9.412%
**3**	Redistributive	Targets the unequal distribution of causes of ill health, and therefore does not benefit the least deprived	As baseline	Increase the length of time to transition by 3.2465%	Increase the length of time to transition by 3.5019%	Increase the length of time to transition by 4.0383%	Increase the length of time to transition by 4.8739%
**4**	Proportionate universalism	Targets the whole population, with increasing benefits along the social gradient that are proportional to the level of disadvantage	Increase the length of time to transition by 2.23%	Increase the length of time to transition by 2.719%	Increase the length of time to transition by 2.974%	Increase the length of time to transition by 3.5067 %	Increase the length of time to transition by 4.3381%
**5**	Removal of inequalities	Socioeconomic inequalities in transition times between states are completely	As baseline	As IMD1	As IMD1	As IMD1	As IMD1

	removed

IMD = Index of Multiple Deprivation
NB: The improvement values for scenarios 1-4 were generated to achieve a 3% reduction in cumulative deaths prevented/postponed compared to the baseline scenario, taking into account the socioeconomic gradient in 2019 age-sex standardised prevalence of basic multimorbidity in the baseline scenario.

The implementation of the scenarios as summarised in Table Q is illustrative of the theoretical policy typologies – the actual values of the improvements modelled are essentially arbitrary. We chose these specific scenario with the aim of modelling examples that can demonstrate differences in how the approaches may impact the future incidence and prevalence of multimorbidity.

Sensitivity analyses

We conducted two sensitivity analyses of our scenario implementations:

A. No improvements in mortality: For all scenarios, we applied the improvement only to transitions between health states (transitions #1, #3, and #5 in Figure A) and not to death.
B. No improvements among the healthy population: For all scenarios, we applied the improvement only to those in either the initial condition, basic multimorbidity or complex multimorbidity health states (transitions #3-7 in Figure A).

1.8. Model outcomes

We calculated and compared the following outcome measures:

1. Numbers and proportions by state in 2049, overall, and by broad age-group
2. Cases prevented/postponed by state between 2019-2049, overall, by IMD quintile, and by broad age-group
   - For each year from 2019-2049, incident cases of 1 condition, basic multimorbidity, and complex multimorbidity under each scenario were compared with the baseline scenario to calculate annual cases prevented or postponed, then summed for the 2019-2049 to give total cases prevented/postponed for each health state.
1. Median years to be lived without multimorbidity, and years to be lived without complex multimorbidity at age 30, overall, and by IMD quintile.
   - Simulants aged 30 in 2019 were simulated until death under each scenario. We then calculated the median number of years without a) multimorbidity (basic or complex), and b) complex multimorbidity at age 30.

Results are presented for the overall simulant population, as well as by broad age-group (aged under 65; aged 65 and over) and IMD quintile. Baseline results are scaled to ONS population projection estimates, with the same weights used for scaling up the scenario results.

1.9. Model assumptions

As with all models, several assumptions had to be made in structuring the microsimulation model. Table S summarises our key assumptions. As mentioned

above, we have assumed that the trajectory of simulants is independent of the trajectory of other simulants. As we used a categorical variable for depicting birth cohort, we have assigned cohorts introduced into the model after 2019 initial start states based on the same proportions as the 1985-1989 birth cohort, and we have assumed they have the same transition probabilities as the 1985-1989 birth cohort. Apart from age, individual sociodemographic characteristics (e.g. IMD quintile and geographic region of residence) are assumed to be fixed once assigned. As two of the main inputs for this model (predicted time to event for each transition; proportion in each health state at the start of the simulation) are derived from analysis of a sample of CPRD Aurum data, we have assumed that these results are generalisable to the whole adult population in England, and that these do not change over time. Multi-state models make strong assumptions relating to competing risks, including independence of transitions and conditional exchangeability of censoring[22], which may not be strictly true. We have used a linear correlation matrix derived from the primary care data to account for some correlations between transitions. Censoring may not be independent of the outcome (conditional exchangeability of censoring)[22] for example if worsening health leads to a move to a residential care home and therefore censoring from the study. Additionally, the competing risk of death could lead to biased estimates of socioeconomic inequalities in accumulation of multimorbidity.

Table R Summary of key model assumptions

Assumption	Details
**Independence of individuals**	Although the majority of the included conditions are non-communicable diseases, health behaviours of individuals are influenced by familial and social networks, as well as neighbourhood factors and societal norms. Independence of individuals is therefore a simplification of reality.
**Partial Independence of transitions**	The multi-state survival analysis models we have used assume independence between transitions. Onset/diagnosis of one condition may influence onset/diagnosis of another, and therefore future transitions. Additionally, as humans do not live indefinitely, the length of time spent in one state is partly determined by the cumulative time spent in preceding states. We have partially accounted for the second of these through use of a correlation matrix when simulating individual life courses.
**All conditions are life-long**	We have assumed that all conditions are life-long and that once diagnosed there is no recovery. This makes the model uni-directional (Supplementary Materials A Figure A).
**Accumulation of chronic condition states is sequential**	We have assumed that individuals pass sequentially through each of the living states defined in our model (Figure A) – e.g. healthy -> 1 condition -> basic multimorbidity. Interim health states cannot be skipped. This was to reduce complexity of the model.
**Source data from primary care health records is representative**	By applying transition times from primary care data to estimates of the general population by age, sex, birth cohort, QIMD, and geographical region, we assume that the health status of each sub-group in our primary care data sample is representative of the corresponding sub-group of the general population, and that this will not change over time.
**Transition times between health states remain constant over time**	We have assumed that the transition times derived by age and birth cohort from CPRD data between 2004-2019 remain constant over time. Cohorts introduced into the model after 2019 were assigned initial start states based on the same proportions as the 1985-1989 birth cohort, and the same transition probabilities as the 1985-1989 birth cohort.
**Individuals do not change regional area or relative quintile of deprivation**	The region and IMD quintile of simulants on entry to the model that informs the duration of time in each health state, regardless of previous exposure and irrespective of any subsequent change in risk factors.
**Scenario assumptions**	We have assumed that improvements apply equally across the life course, and that prevention reduces mortality as well as slowing accumulation (except for in sensitivity analysis B).<br>For the inequalities removal scenario (#5), we have assumed that the more deprived quintile can have the transition rates of the least deprived, i.e. no residual effect of life course exposures

Several of the assumptions within our model relate to the underlying structure and how we have implemented chronic conditions and multimorbidity within our work. Firstly, we have assumed all conditions are chronic with no recovery, so chronic conditions and modelled health states are life-long. This means that accumulation of multimorbidity can only be progressive, with no remission. Secondly, we have assumed that each health state is passed through sequentially, from healthy -> an

initial chronic condition -> basic multimorbidity -> complex multimorbidity, and that interim states cannot be skipped.
Our implementation of prevention scenarios has several additional assumptions. Firstly, we have assumed that improvements apply equally across the life course. Secondly, we modelled improvements between all states, including transitions to death; i.e. we assumed that prevention reduces mortality as well as slowing accumulation. We relaxed this assumption in sensitivity analysis A (Section 1.6.1).

1.10. Example illustration of one simulant

To illustrate how the microsimulation works, let us look at an example of an individual with the following characteristics: sex = male, IMD quintile = 1 (least deprived), region = London, birth cohort = 1985-89, start age = 30, and start state = healthy. From the healthy state, there are two possible subsequent states: 1 condition (T1) or death (T2). Table T shows the predicted age at transition from the healthy state to either the initial chronic condition state (T1), or death (T2) for selected quantiles of the distribution for this set of covariates. A random number is picked for each transition and amended by the correlation matrix (Section 1.4.4) and, if calibration applied, the calibration factor for the transition (see Section 1.11) The resulting random number is then used to select the quantile of the distribution to identify from the lookup table for that transition.

Table S - Look-up table for an example individual: male, IMD quintile 1 (least deprived), London, birth cohort 1985-89, start age 30, start state healthy

**Quantile**	**T1 Healthy → 1 condition**<br>**Age at transition (standard error)**	**T2 Healthy → Death**<br>**Age at transition (standard error)**
**0**	30 (<0.001)	30 (<0.001)
**0.1**	33.4 (0.083)	79.8 (3.201)
**0.2**	36.5 (0.144)	88.7 (3.575)
**0.3**	39.5 (0.196)	94.3 (3.843)
**0.4**	42.6 (0.246)	98.6 (4.063)
**0.5**	45.7 (0.297)	102.2 (4.258)
**0.6**	49.0 (0.352)	105.6 (4.444)
**0.7**	52.8 (0.413)	108.8 (4.630)
**0.8**	57.3 (0.489)	110.0 (4.833)
**0.9**	63.7 (0.603)	110.0 (5.090)
**1**	110.0 (<0.001)	110.0 (<0.001)

T# = Transition number

The random number for transition 1 (healthy to initial chronic condition) is 0.472242, so the model interpolates between quantile 4 (transition age: 43) and quantile 5 (transition age: 46). The resulting transition age is 45, and therefore time from entry to transition is 15 years.
The random number for transition 2 (healthy to death) is 0.744857, so the model interpolates between quantile 7 (transition age: 109) and quantile 8 (transition time: 110 years). The resulting transition age is 110, and therefore time from entry to transition is 80 years.

As transition 1 (healthy to initial condition) occurs earlier than transition 2 (healthy to death), the example simulant transitions from healthy to 1 condition after 15 years at age 45. The same process is then repeated for all subsequent transitions until the simulant reaches the death state.

1.11. Microsimulation model uncertainty

Uncertainty analysis is a way of quantifying variation in the model outcome[23]. We have used a first-order Monte Carlo approach to quantify uncertainty as this captures stochastic uncertainty and heterogeneity. The use of a microsimulation model at an individual-level allows the modelling of heterogeneity of individuals such as by age, sex, area-level socioeconomic status, and geographical region.
For these theoretical policy scenarios, the interest is in the relative differences in impact between the baseline and theoretical scenarios, rather than the accurate prediction of the future number of cases. Given that relative impact is the focus, we have not modelled parameter uncertainty (second-order Monte Carlo approach) for the theoretical scenarios as the uncertainty in these shared input parameters is likely to cancel out between scenarios. The scenario results in this paper are reported with first-order Monte Carlo uncertainty: the median and 95% uncertainty intervals from 100 model iterations with the parameter inputs fixed at the mean for each variable. Sensitivity analyses are run with 10 model iterations, and compared to the first 10 iterations of the baseline scenario.

1.12. Microsimulation model validation and calibration

Validation is a set of methods for assessing the accuracy of a model's ability to make predictions[24]. There is not a definitive threshold for validity vs invalidity, and validity of one application of the model does not translate to a blanket validity of all potential applications. There are five main types of model validity: face validity, internal validity (verification), cross validity, external validity, and predictive validity[24]. The International Society for Pharmacoeconomics and Outcomes Research (ISPOR) – Society for Medical Decision Making (SMDM) Task Force on Good Research Practices in Modeling Studies recognises that not all types of validation are possible or appropriate for all simulation models[24]. For this study, we conducted face and internal validation. Model calibration is the process of comparing model outputs with existing empirical data in order to determine parameter values that produce model outputs similar to observed data[24].
Face validity of the model was discussed among co-authors, as well as in presentations of the model to our research team and a research group at another university. We also calculated age-sex standardised rates (prevalence, incidence and case-fatality), and the slope index of inequality as a measure of absolute inequalities in crude prevalence to consider face validity of the model results in comparison with the results from the direct analysis of CPRD Aurum data (previously published).[4]
AH, MB, and CK conducted interim code checks to ensure internal validation. Validation of the survival analysis fit inputs into the microsimulation comprised goodness of fit metrics, and visual comparison of fitted survival curves against Kaplan Meier curves.
As the CPRD Aurum sample we used for model inputs contains right-censored data, we used a subset of the sample for dependent external validation. In order to

compare the proportion of simulants in each state at a certain time with the observed proportion in the CPRD sample, we selected all individuals in our primary care dataset who had at least 10 years of follow-up within the study period, or who died within 10 years of entering the study (summary of demograpic characteristics in Table T). We used this subset of the CPRD sample as our validation dataset, and compared simulated outputs with observed outputs after 10 years. The main measure for comparison was the proportion of individuals in each state at 10 years by sex and IMD quintile.

Table T - Demographic characteristics of validation subset

		**Validation sub-sample** %*	**Survival analysis sample** %*
**Overall sample size**		N = 322,207	N = 762,803
**Sex**	Male	49.4	49.6
	Female	50.6	50.4
**Age in years**[×]**†**		48 (36,63)	41 (31,56)
**IMD quintile**	1 (least deprived)	24.4	21.6
	2	21.6	20.4
	3	19.5	19.7
	4	17.9	20.1
	5 (most deprived)	16.6	18.2
**Birth cohort**	<1930	9.4	5.5
	1930-1934	5.9	3.5
	1935-1939	6.5	4
	1940-1944	7.2	4.7
	1945-1949	8.8	6.2
	1950-1954	8.2	6.2
	1955-1959	9.0	7.1
	1960-1964	10.4	8.9
	1965-1969	10.5	10.3
	1970-1974	8.9	11.2
	1975-1979	6.9	12.3
	1980-1984	6.0	14.4
	1985-1989	2.2	5.8
**Region**	London	12.3	19.7
	South West	14.1	13.5
	South Central	12.5	12.1
	South East Coast	7.8	7.7
	West Midlands	18.2	16.1
	East Midlands	2.3	2.7
	East of England	5.9	5.4
	North West	18.2	15.5
	Yorkshire and the Humber	4.0	3.7
	North East	4.7	3.6

**Initial state**	Healthy	46.2	52.7
	1 condition	20.7	29.6
	Basic multimorbidity	16.8	14.6
	Complex multimorbidity	16.3	13.1

*Proportions are presented in place of absolute numbers given the difference in size.
IMD = Index of Multiple Deprivation

For calibration, we used the same subset of the CPRD data as for the dependent external validation. The proportion of simulated individuals in each state after 10 years was compared to the observed proportion after 10 years. The level of acceptable bias (difference between simulated and observed proportion in each state) was set to 0.001. Depending on the direction of the bias, the calibration factor will either bound the random number at the top end (e.g. maximum quantile that can be drawn is 0.9) or at the bottom end (e.g. minimum quantile that can be drawn is 0.1). For each starting state (apart from complex multimorbidity which has only one subsequent transition to death), the same calibration factor was applied to the two possible subsequent transitions, this was repeated in increments until the simulated results were within 0.001 point from the observed proportions.

Table V presents the proportion of the observed and simulated (before and after calibration) validation dataset in each state 10 years after entry into the study by sex, and shows that the model validates well in dependent external validation. Before calibration, there is less than a 1 percentage point difference between the simulated and observed proportion in all states. Calibration reduces these differences to less than a 0.01 percentage point difference for all states apart from death, which remains slightly underestimated. The final calibration factors applied to each transition are presented in Table W.

Table U – Summary of external dependent validation

**Sex**	**State after 10 years**	**Observed (%)**	**No calibration**		**With calibration**	
			Simulated (%)	Absolute difference	Simulated (%)	Absolute difference
**Male**	Healthy	12.4	12.6	0.23	12.4	<0.01
	1 condition	6.5	7.1	0.62	6.5	<0.01
	Basic multimorbidity	9.3	9.2	-0.11	9.3	<0.01
	Complex multimorbidity	14.3	14.3	0.08	14.3	<0.01
	Death	6.9	6.1	-0.81	6.9	-0.4
**Female**	Healthy	8.2	8.6	0.35	8.2	<0.01
	1 condition	7.1	7.4	0.31	7.1	<0.01
	Basic multimorbidity	10.2	10.3	0.02	10.2	<0.01
	Complex multimorbidity	18.4	18.2	-0.20	18.4	<0.01
	Death	6.6	6.1	-0.49	6.6	-0.04

Table V -Calibration factors applied to the transitions from each starting state

	**Sex**	
**Start state (Transition numbers)**	Male	Female
**Healthy (T1 & T2)**	0.9820	0.9720
**Incident condition (T3 & T4)**	0.9554	0.9750
**Basic multimorbidity (T5 & T6)**	0.9491	0.9506
**Complex multimorbidity (T7**	0.8169	0.8629

T# = Transition number

1.13. Microsimulation model limitations

Like all simulation models, we have made assumptions and simplifications in developing our microsimulation. A brief summary of key points is included in the main text, and further discussed here.

We have assumed that all conditions are lifelong once diagnosed and recorded, that individuals in our model do not change regional area or relative quintile of deprivation and that our source data from primary care health records is representative of the general population. The quality of the underlying data impacts our results and the conclusions that can be drawn. The validity of diagnostic codes within CPRD varies by condition, and updates in treatment guidelines and Quality Outcomes Framework pay for performance rules might have influenced the propensity to code certain conditions over time. Health-seeking behaviour, frequency of general practice visits, length of registration, propensity to seek care, and pre-existing conditions all influence the likelihood of initial and subsequent disease diagnosis, and these factors may differ by socioeconomic deprivation.

For resource reasons, we did not have a linkage for our primary care data to secondary care, although the majority of chronic health conditions are managed within primary care and are, therefore, likely to be recorded. In recent work examining the setting of diagnosis recording in linked primary and secondary care records, an estimated 85% of all diagnoses were present in primary care, and this underestimation may be gently increasing by deprivation[25]. We may, therefore, slightly underestimate multimorbidity prevalence and baseline inequality in our

current study. Similarly, we were unable to link to ONS mortality records; however, the presence/absence of death is generally well recorded within CPRD[7].

We excluded individuals with missing data on sociodemographic variables (N=1,614); this may have introduced bias into our data if those with missing data have different health than those without; however, given the small amount of missingness, any bias is likely to be minimal. We calibrated and validated our microsimulation model against observed data for people in our CPRD sample with at least 10 years of follow-up, which was slightly older and less deprived than our analysis sample. This was necessary as, during the calibration, we needed to minimise the bias from censoring. The underlying assumption is that the transition times between states for those lost to follow-up within 10 years by age, sex, region, birth cohort and QIMD are similar to those observed for at least 10 years. Those lost to follow-up within 10 years are most likely due to spatial mobility, and there is sparse evidence to suggest that spatial mobility was not affecting the likelihood of limiting long-term illness later in life when socioeconomic factors were controlled for[26]. Therefore, we do not expect our approach to have introduce any major bias given we have stratified by region and IMD quintiles.

Although parametric models can extrapolate beyond values provided in the original input data, for time trends, this makes the assumption that parameter estimates are stable over time, i.e. our baseline transition times derived from CPRD data between 2004-2019 will remain constant, regardless of secular trends in risk factor exposure levels. This is a strong assumption. Advancements in health systems, such as new pharmaceuticals and technologies, or improvements in risk factor trends, such as systolic blood pressure, could lead to slower transition times between states than we projected, and these advancements may have differential effects across socioeconomic groups.

Our modelling scenarios have not looked at pathways through which socioeconomic inequalities in multimorbidity develop, and further work to understand these is important for developing successful, equitable policies to act on these pathways[27]. Instead, we modelled scenarios based on commonly used theoretical frameworks for understanding and explaining socioeconomic inequalities in health. We focused on

socioeconomic inequalities within this work and assumed that the modelled improvements were applied evenly across the life course. However, given that there are stark inequalities by sex and between regions, it is likely that policies to target these other sociodemographic inequalities would also have the potential to reduce the multimorbidity burden at the population level.

Given the theoretical nature of our scenarios, we cannot draw concrete conclusions about actual policies or recommend specific policy action. The size of the health improvements and inequalities reduction are, to a certain extent, driven by our modelling choices, and not all conditions will be equally amenable to prevention strategies. Empirical evidence on the effectiveness and impact on inequalities of specific policies at reducing multimorbidity incidence is scarce, and evaluation of such policies would be time- and resource-intensive. There are, however, existing examples of implemented health policies that can be mapped to each theoretical scenario. Targeted policies (scenario 1) include programmes such as income-based vouchers for healthy food[28] or early education centres (e.g. Sure Start centres in the UK[29]) in deprived areas. NHS health checks for all over-40s in the UK with targeted interventions to reach more deprived communities is one example of a universal policy focusing on reducing the health outcome gap between the most and least deprived (scenario 2)[30]. A universal basic income policy, currently being piloted in several places, including Wales[31], could be considered redistributive (scenario 3) as the least deprived would have minimal benefit as they are not in special need, similarly to other progressive taxation systems[32]. Two types of policies can be considered for proportionate universalism (scenario 4). Firstly, policies could address an existing social gradient in exposure, such as widespread low-emission zones, where a universal policy would have a greater effect on more deprived groups as they have higher exposure[32]. Alternatively, proportionate universalism policies can be those that explicitly increase resource allocation relative to increased need, for example, with the NHS Scotland Resource Allocation formula for the geographical distribution of NHS resources based on need[33].

In comparing a fixed target of mortality reductions for scenarios 1-4, we have also implicitly assumed that each scenario implementation would require the same resources for the prevention/postponement of one death, regardless of the health

state or sociodemographic characteristics of the individual. In reality, however, the cost of interventions targeted at specific high-risk populations likely differs from those rolled out across a population[34]. Similarly, the cost, cost-effectiveness, and time frame for benefits likely vary between interventions[34]. We have also assumed that prevention policies would delay disease onset and improve survival from all health states, as with smoking cessation[35]. Beyond theory, empirical research on policy effectiveness with multimorbidity outcomes will be required to select the most feasible, evidence-based, effective, cost-effective and equitable policies. It is clear from existing evidence that a focus on individual-level behaviour change will be insufficient to address current socioeconomic inequalities adequately[36].

Although the fifth scenario of removing socioeconomic inequalities (removing socioeconomic inequalities in transition times) is ideal, it demonstrates the potential scope for health improvement if there was no social stratification between groups in society[37]. An inherent assumption within this scenario is that disadvantaged groups will be able to reach the same level of health progression as those from the least deprived group and that past histories of deprivation will have no future impact. This likely overestimates the impacts of removing social stratification[38].

Our model was developed using primary care data for adults registered at GP practices in England, a high-income country with a universal healthcare system, where healthcare visits are free at the point of use. Our results may be cautiously generalised to other high-income settings with similar levels of multimorbidity and similar healthcare systems. However, our results are unlikely to apply to countries with a high burden of communicable diseases or those with substantially lower or higher life expectancy. Additionally, our model is based on underlying socioeconomic inequalities in multimorbidity trajectories, which are likely to be linked to the level of equality/inequality within a country. Under these strong assumptions that multimorbidity trajectories are the same across populations, our model could be applied in other settings where age and sex structure are available for population projections.

References

1. Head, A., Birkett, M., Fleming, K., Kypridemos, C. & O'Flaherty, M. Socioeconomic inequalities in accumulation of multimorbidity in England from 2019 to 2049: a microsimulation projection study. *Lancet Public Health* **9**, e231–e239 (2024).
2. The Academy of Medical Sciences. *Multimorbidity: A Priority for Global Health Research*. 1–127 https://acmedsci.ac.uk/file-download/82222577 (2018).
3. Ho, I. S. S. *et al.* Examining variation in the measurement of multimorbidity in research: a systematic review of 566 studies. *Lancet Public Health* **6**, e587–e597 (2021).
4. Head, A. *et al.* Inequalities in incident and prevalent multimorbidity in England, 2004–19: a population-based, descriptive study. *Lancet Healthy Longev.* **2**, e489–e497 (2021).
5. Deshpande, A. D., Harris-Hayes, M. & Schootman, M. Epidemiology of Diabetes and Diabetes-Related Complications. *Phys. Ther.* **88**, 1254–1264 (2008).
6. MacRae, C. *et al.* The impact of varying the number and selection of conditions on estimated multimorbidity prevalence: A cross-sectional study using a large, primary care population dataset. *PLOS Med.* **20**, e1004208 (2023).
7. Gallagher, A. M., Dedman, D., Padmanabhan, S., Leufkens, H. G. M. & de Vries, F. The accuracy of date of death recording in the Clinical Practice Research Datalink GOLD database in England compared with the Office for National Statistics death registrations. *Pharmacoepidemiol. Drug Saf.* **28**, 563–569 (2019).
8. Hougaard, P. Multi-state Models: A Review. *Lifetime Data Anal.* **5**, 239–264 (1999).
9. Jackson, C. H. Flexsurv: A platform for parametric survival modeling in R. *J. Stat. Softw.* **70**, (2016).
10. Office for National Statistics. Guide to calculating national life tables. https://www.ons.gov.uk/peoplepopulationandcommunity/healthandsocialcare/healthandlifeexpectancies/methodologies/guidetocalculatingnationallifetables (2019).
11. Wolf, A. *et al.* Data resource profile: Clinical Practice Research Datalink (CPRD) Aurum. *Int. J. Epidemiol.* **48**, 1740–1740g (2019).
12. NHS Digital. Quality and Outcomes Framework, 2019-20. https://digital.nhs.uk/data-and-information/publications/statistical/quality-and-outcomes-framework-achievement-prevalence-and-exceptions-data/2019-20 (2020).
13. CPRD. 2020-06 CPRD Aurum Release Notes. (2020).
14. Smith, T. *et al. The English Indices of Deprivation 2015 - Technical Report. Department for Communities and Local Government. London, UK.* pp 126 (2015).
15. Head, A. The Lancet Healthy Longevity Trends in inequalities in incident and prevalent multimorbidity : a population-based descriptive study in England , 2004 to 2019 Address for Correspondence : *Lancet Healthy Longev.* (2019).
16. CALIBER. https://www.caliberresearch.org/portal/phenotypes/chronological-map.
17. Parametric survival modeling. https://devinincerti.com/2019/06/18/parametric_survival.html.
18. Rebora, P., Salim, A. & Reilly, M. bshazard: A Flexible Tool for Nonparametric Smoothing of the Hazard Function.
19. Canchola, A. J. *et al.* Cox regression using different time-scales.
20. Office for National Statistics. Lower layer Super Output Area population estimates (supporting information) - Mid-2019 Edition. https://www.ons.gov.uk/peoplepopulationandcommunity/populationandmigration/

populationestimates/datasets/lowersuperoutputareamidyearpopulationestimates (2021).
21. Office for National Statistics. *National Population Projections 2020-Based Interim*. https://www.ons.gov.uk/peoplepopulationandcommunity/populationandmigration/populationprojections/bulletins/nationalpopulationprojections/2020basedinterim (2022).
22. Mansournia, M. A., Nazemipour, M. & Etminan, M. A practical guide to handling competing events in etiologic time-to-event studies. *Glob. Epidemiol.* **4**, 100080 (2022).
23. Briggs, A. H. *et al.* Model Parameter Estimation and Uncertainty Analysis: A Report of the ISPOR-SMDM Modeling Good Research Practices Task Force Working Group–6. *Med. Decis. Making* **32**, 722–732 (2012).
24. Task, P. *et al.* Model Transparency and Validation : A Report of the ISPOR-SMDM Modeling. 733–743 (2012) doi:10.1177/0272989X12454579.
25. Wang, S. *et al.* Inequalities in the prevalence recording of 205 chronic conditions recorded in primary and secondary care for 12 million patients in the English National Health Service. *BMC Med.* **22**, 570 (2024).
26. Forrest, L., Dibben, C., Feng, Z., Deary, I. & Popham, F. Social and spatial mobility and self-reported heath in older-age: linkage of the Scottish Longitudinal Study to the Scottish Mental Survey 1947. *Int. J. Popul. Data Sci.* **3**, (2018).
27. Alfonzo, L. F. *et al.* Theoretical explanations for socioeconomic inequalities in multimorbidity: a scoping review. *BMJ Open* **12**, e055264 (2022).
28. Parnham, J. *et al.* Is the healthy start scheme associated with increased food expenditure in low-income families with young children in the United Kingdom? *BMC Public Health* **21**, 2220 (2021).
29. Cattan, S., Conti, G., Ginja, R., Farquharson, C. & Pecher, M. *The Health Impacts of Sure Start*. http://default/publications/health-impacts-sure-start (2021) doi:10.1920/BN.IFS.2021.BN0332.
30. Kypridemos, C. *et al.* Cardiovascular screening to reduce the burden from cardiovascular disease: microsimulation study to quantify policy options. *BMJ* **353**, i2793 (2016).
31. Welsh Government. Basic income pilot for care leavers: overview of the scheme. *GOV.WALES* https://www.gov.wales/basic-income-pilot-care-leavers-overview-scheme (2023).
32. Benach, J., Malmusi, D., Yasui, Y. & Martínez, J. M. A new typology of policies to tackle health inequalities and scenarios of impact based on Rose’s population approach. *J Epidemiol Community Health* **67**, 286–291 (2013).
33. Macdonald, W., Beeston, C. & McCullough, S. *Proportionate Universalism and Health Inequalities*. https://www.healthscotland.com/uploads/documents/24296-ProportionateUniversalismBriefing.pdf (2014).
34. Masters, R., Anwar, E., Collins, B., Cookson, R. & Capewell, S. Return on investment of public health interventions: A systematic review. *J. Epidemiol. Community Health* **71**, 827–834 (2017).
35. Cho, E. R., Brill, I. K., Gram, I. T., Brown, P. E. & Jha, P. Smoking Cessation and Short- and Longer-Term Mortality. *NEJM Evid.* **3**, EVIDoa2300272 (2024).
36. Katikireddi, S. V., Higgins, M., Elizabeth Smith, K. & Williams, G. Health inequalities: the need to move beyond bad behaviours. *J Epidemiol Community Health* **67**, 715–716 (2013).
37. Diderichsen, F., Evans, T. & Whitehead, M. The Social Basis of Disparities in Health. in *Challenging Inequities in Health: From Ethics to Action* (eds Evans, T.,

Whitehead, M., Diderichsen, F., Bhuiya, A. & Wirth, M.) 0 (Oxford University Press, 2001). doi:10.1093/acprof:oso/9780195137408.003.0002.
38. Bell, R. & Marmot, M. Life course approach to understanding inequalities in health in later life. in *Oxford Textbook of Geriatric Medicine* (eds Michel, J.-P., Beattie, B. L., Martin, F. C. & Walston, J.) 0 (Oxford University Press, 2017). doi:10.1093/med/9780198701590.003.0010.
