# Supplementary material for: Exploring theoretical policy options for reducing socioeconomic inequalities in multimorbidity: A microsimulation study in England from 2019–2049
Source: J Multimorb Comorb. 2026 Jun 23;16:26335565261441403. doi: 10.1177/26335565261441403 (PMC13305289; doi:10.1177/26335565261441403)
Supplement: Supplemental material - Exploring theoretical policy options for reducing socioeconomic inequalities in multimorbidity: a microsimulation study in England from 2019–2049 [file sj-pdf-2-cob-10.1177_26335565261441403.pdf]

S2 Supporting Information– Supplementary Results

2.1. Difference in proportions living without multimorbidity in 2049

Table A - Difference in proportions living without multimorbidity in 2049 by age-group and scenario (percentage points)

**Scenario**	**Index of multiple deprivation (1 = least deprived)**				
	1	2	3	4	5
**Age < 65**					
**1 Targeted**	-	-	-	0.28	0.60
**2 Universal + focus on gap**	0.38	0.36	0.36	0.40	0.45
**3 Redistributive**	-	0.43	0.44	0.49	0.53
**4 Proportionate universalism**	0.31	0.35	0.38	0.43	0.47
**5 Inequalities removal**	-	0.49	0.60	0.98	1.41
**Age 65 and over**					
**1 Targeted**	-	-	-	-0.13	-0.55
**2 Universal + focus on gap**	0.05	0.05	0.04	0.04	0.02
**3 Redistributive**	-	0.05	0.05	0.05	0.02
**4 Proportionate universalism**	0.03	0.04	0.05	0.04	0.02
**5 Inequalities removal**	-	-0.19	-0.42	-0.54	-1.09

2.2. Difference in numbers living without multimorbidity in 2049

Table B - Difference in numbers living without multimorbidity in 2049 by age-group and scenario

**Scenario**	**Index of multiple deprivation (1 = least deprived)**				
	1	2	3	4	5
**Age < 65**					
**1 Targeted**	-	-	-	66,000	160,000
**2 Universal + focus on gap**	37,000	44,000	52,000	74,000	87,000
**3 Redistributive**	-	52,000	65,000	90,000	100,000
**4 Proportionate universalism**	29,000	43,000	56,000	78,000	92,000
**5 Inequalities removal**	-	65,000	110,000	220,000	340,000
**Age 65 and over**					
**1 Targeted**	-	-	-	8,800	14,000
**2 Universal + focus on gap**	20,000	18,000	18,000	20,000	21,000
**3 Redistributive**	-	21,000	23,000	24,000	25,000
**4 Proportionate universalism**	15,000	18,000	19,000	21,000	22,000
**5 Inequalities removal**	-	16,000	17,000	30,000	33,000

2.1. Total cases prevented or postponed by broad age-group

Figure A- Total incident cases prevented/postponed 2019-2049 among those aged under 65 compared to the baseline scenario

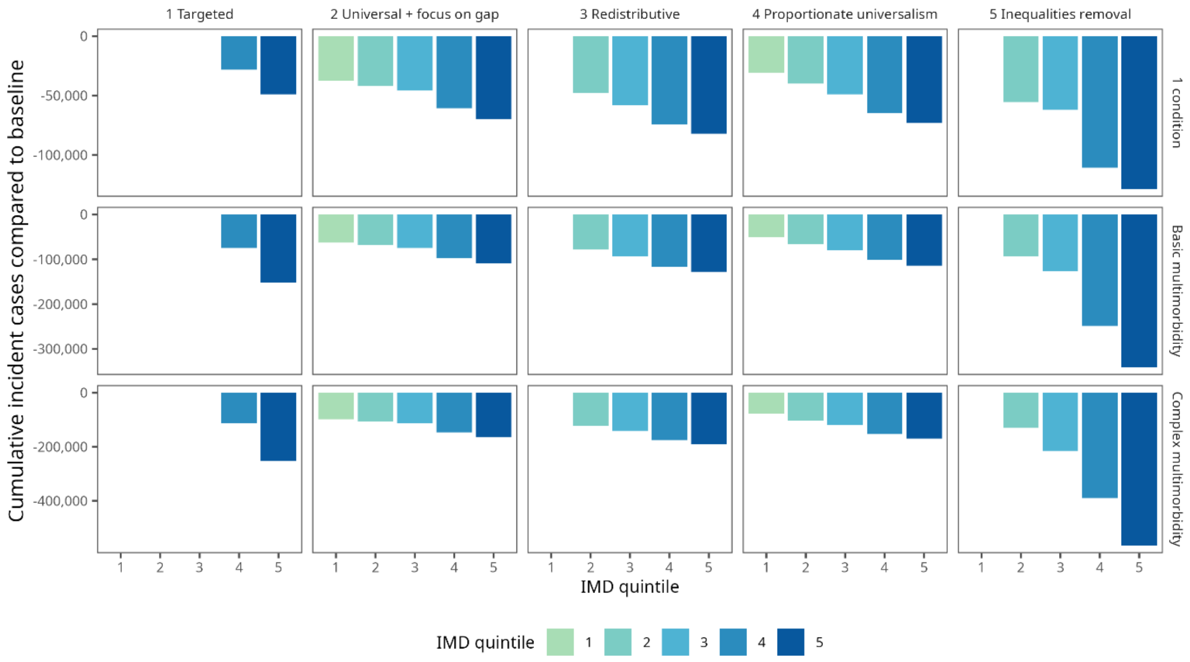

IMD = Index of Multiple Deprivation (1 = least deprived quintile); negative numbers are cases prevented/postponed compared to the baseline scenario

Figure B- Total incident cases prevented/postponed 2019-2049 among those aged 65 and over compared to the baseline scenario

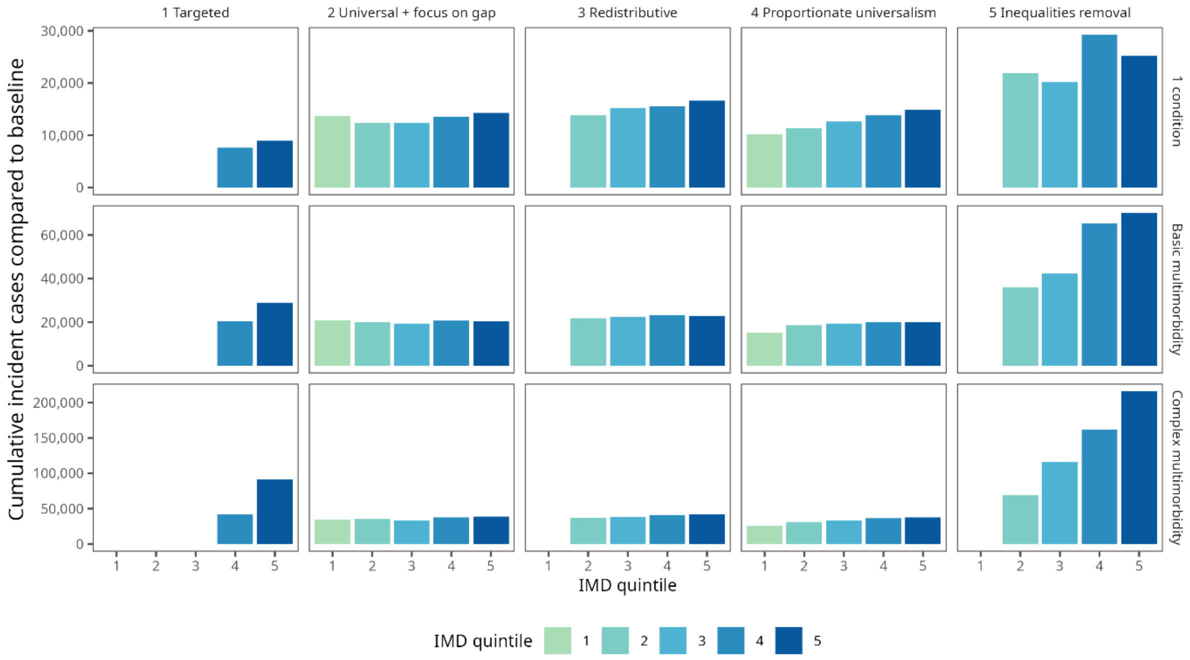

IMD = Index of Multiple Deprivation (1 = least deprived quintile); negative numbers are cases prevented/postponed compared to the baseline scenario

2.2. Sensitivity analyses: total cases prevented or postponed by IMD quintile

Figure C - Total incident cases prevented/postponed 2019-2049 compared to the baseline scenario – sensitivity analysis A: no improvement in mortality transitions

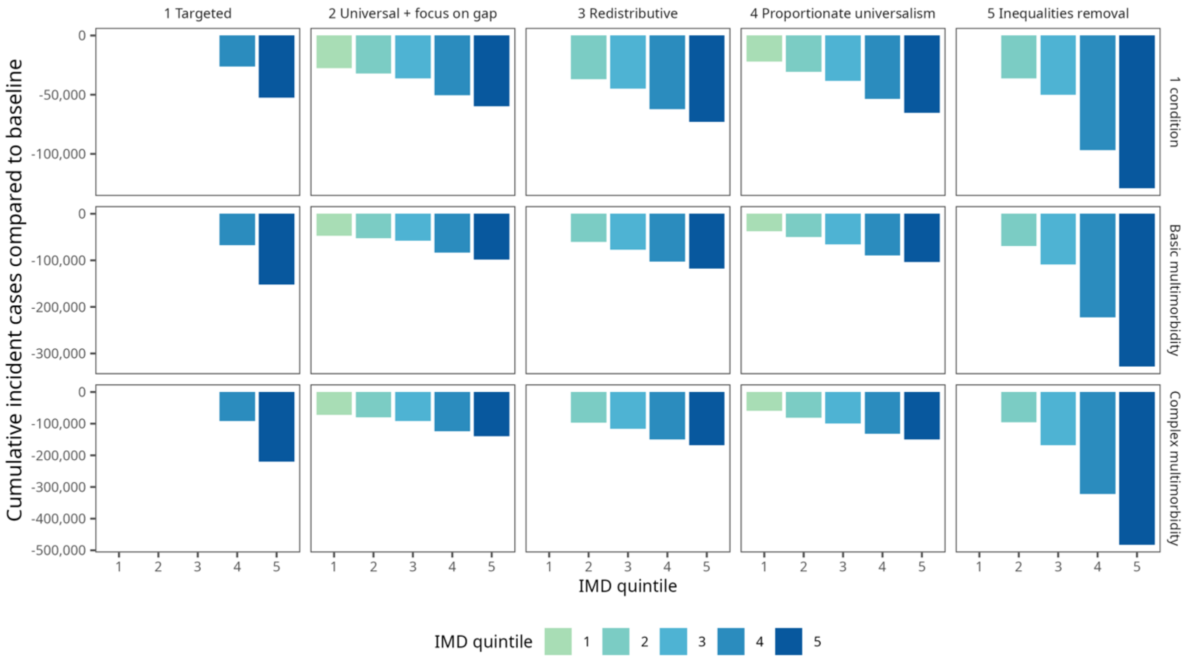

IMD = Index of Multiple Deprivation (1 = least deprived)

Figure D Total incident cases prevented/postponed 2019-2049 compared to the baseline scenario – sensitivity analysis B: no improvement in transitions from the healthy state

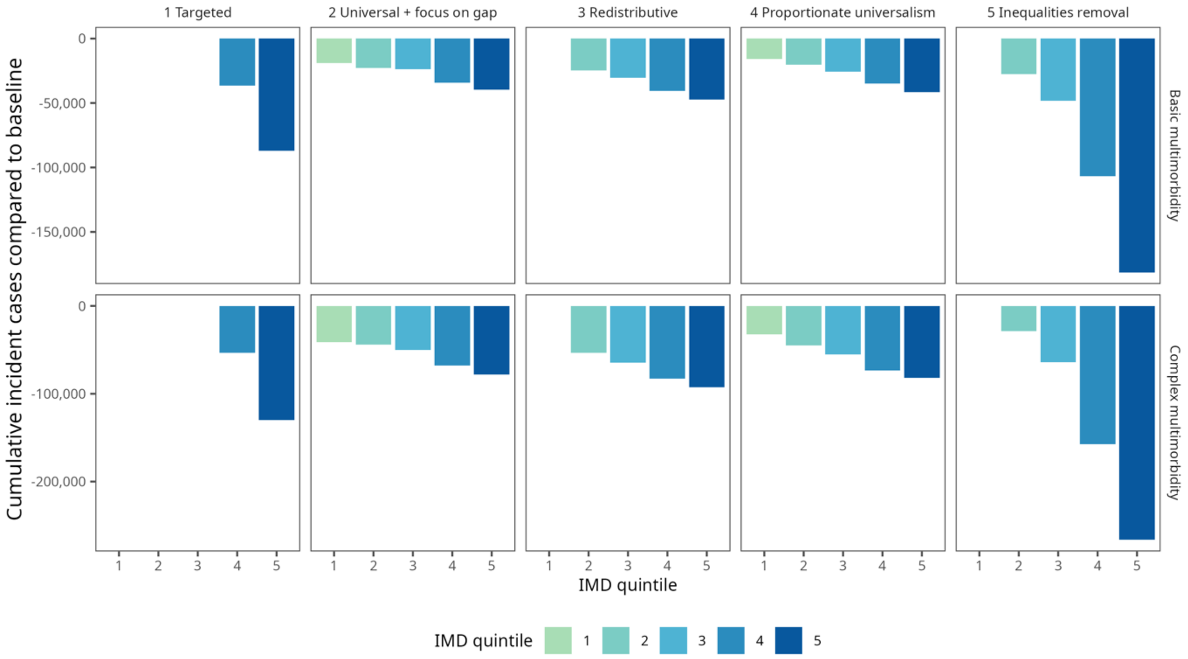

IMD = Index of Multiple Deprivation (1 = least deprived)

2.3. Sensitivity analyses: Difference in median expected years lived without basic and complex multimorbidity at age 30

Figure E – Difference in median expected years lived without basic and complex multimorbidity at age 30: a) by IMD quintile and scenario, compared to baseline scenario; b) by IMD quintile and scenario, compared to the least deprived IMD quintile (IMD1) Sensitivity analysis A: no improvement in mortality transitions

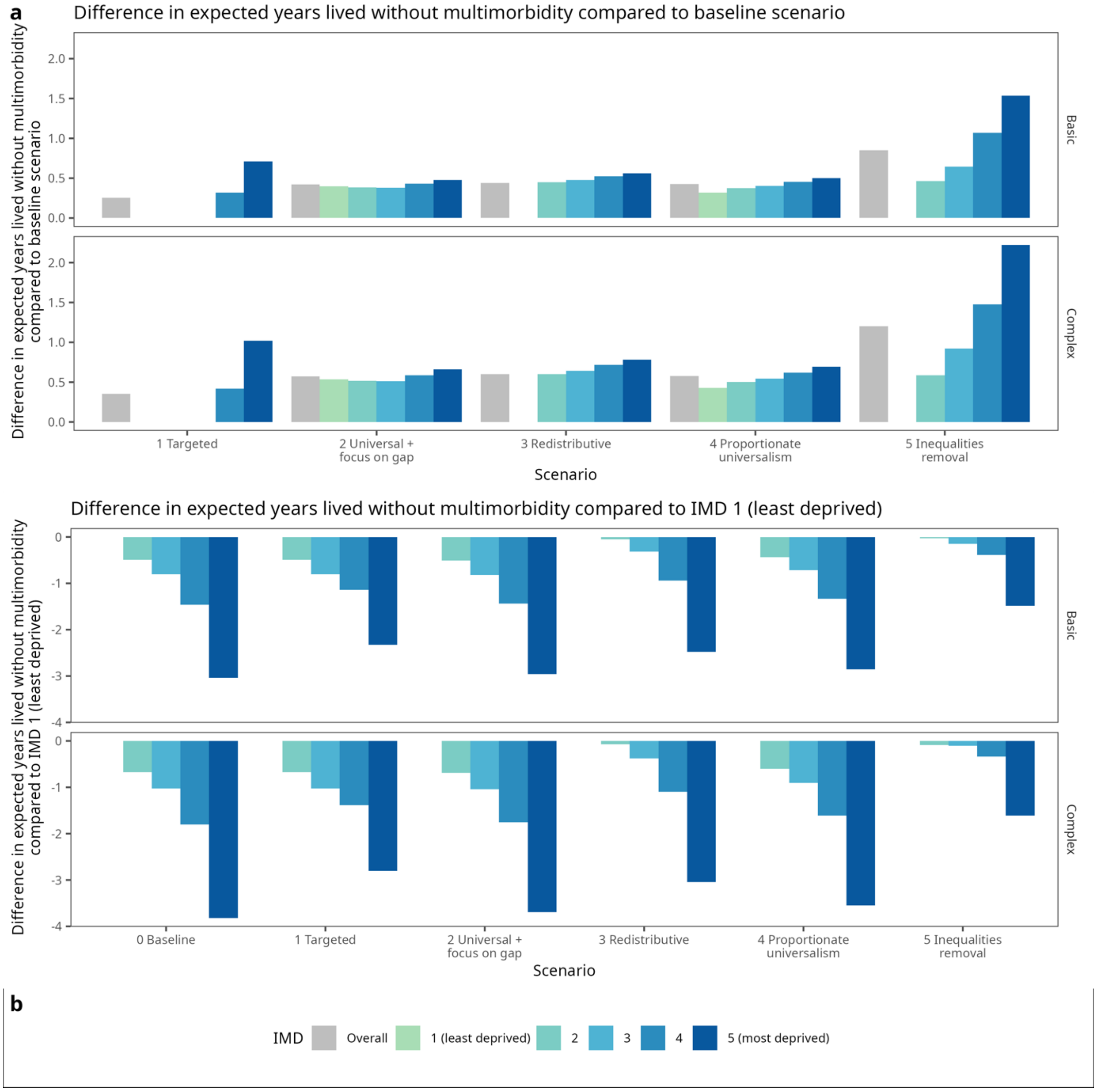

IMD = Index of Multiple Deprivation (1 = least deprived quintile)

Figure F – Difference in median expected years lived without basic and complex multimorbidity at age 30: a) by IMD quintile and scenario, compared to baseline scenario; b) by IMD quintile and scenario, compared to the least deprived IMD quintile (IMD1) Sensitivity analysis B: no improvement in transitions from the healthy state

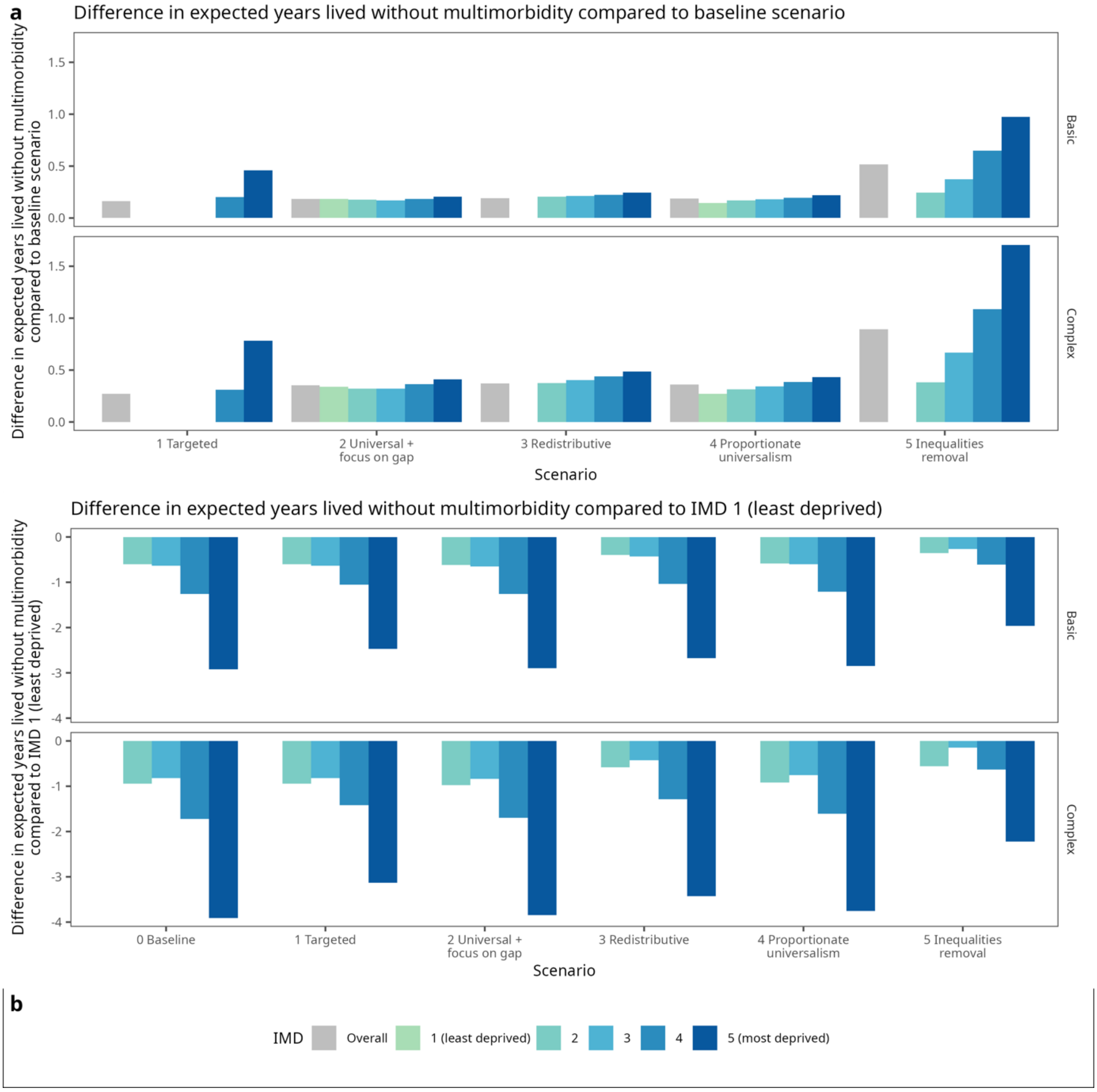

IMD = Index of Multiple Deprivation (1 = least deprived quintile)

2.4. Scenario summary by key outcomes

Table C - Summary of impact of scenarios for key outcomes

Outcome	Ranking (greatest impact first)
Overall cases prevented/postponed	5 > 3 ≈ 2 > 4 > 1
Median years without MM (overall)	5 > 4 ≈ 2 ≈ 3 > 1
Reduction in inequalities gap (under 60s)	5 > 1 > 3 > 4 > 2

Scenario 1 Targeted; 2 Universal with a focus on the gap; 3 Redistributive; 4 Proportionate universalism; 5 Removal of inequalities

We would emphasise, however, that a central finding of our study is that there is no single "optimal" scenario — the ranking of scenarios varies depending on which outcome is prioritised, and this trade-off is itself an important message for policy.
