# Supplementary material for: Exploring theoretical policy options for reducing socioeconomic inequalities in multimorbidity: A microsimulation study in England from 2019–2049
Source: J Multimorb Comorb. 2026 Jun 23;16:26335565261441403. doi: 10.1177/26335565261441403 (PMC13305289; doi:10.1177/26335565261441403)
Supplement: Supplemental material - Exploring theoretical policy options for reducing socioeconomic inequalities in multimorbidity: a microsimulation study in England from 2019–2049 [file sj-pdf-3-cob-10.1177_26335565261441403.pdf]

S3 Supporting Information– Study protocol

INDEPENDENT SCIENTIFIC ADVISORY COMMITTEE (ISAC) PROTOCOL APPLICATION FORM

PART 1: APPLICATION FORM

IMPORTANT

Both parts of this application must be completed in accordance with the guidance note 'Completion of the ISAC Protocol Application Form', which can be found on the CPRD website (https://cprd.com/research-applications).

**FOR ISAC USE ONLY**	
**Protocol No. -**	**Submission date -**

**GENERAL INFORMATION ABOUT THE PROPOSED RESEARCH STUDY**
**Study Title (Max. 255 characters including spaces)**<br><br>Equity dynamics of multimorbidity in England over time: a descriptive study on trends of incident and prevalent multimorbidity by age, sex, and socioeconomic status
**Research Area** (place '**X**' in all boxes that apply)

Drug Safety		Economics	
Drug Utilisation		Pharmacoeconomics	
Drug Effectiveness		Pharmacoepidemiology	
Disease Epidemiology	X	Methodological	
Health Services Delivery			

Chief Investigator

Title:	Dr
Full name:	Kate Fleming
Job title:	Senior Lecturer in Social Epidemiology
Affiliation/organisation:	Department of Public Health and Policy, University of Liverpool

Email address:	Kate.Fleming@liverpool.ac.uk
CV Number (if applicable):	481_15
Will this person be analysing the data? (Y/N)	N

Corresponding Applicant

Title:	Ms
Full name:	Anna Head
Job title:	PhD Candidate
Affiliation/organisation:	Department of Public Health and Policy, University of Liverpool
Email address:	Anna.head@liverpool.ac.uk
CV Number (if applicable):	

List of all investigators/collaborators

Title:	Ms
Full name:	Anna Head
Job title:	PhD Candidate
Affiliation/organisation:	Department of Public Health and Policy, University of Liverpool
Email address:	Anna.head@liverpool.ac.uk
CV Number (if applicable):	
Will this person be analysing the data? (Y/N)	Y

Title:	Professor
Full name:	Martin O'Flaherty
Job title:	Professor of Epidemiology
Affiliation/organisation:	Department of Public Health and Policy, University of Liverpool
Email address:	moflaher@liverpool.ac.uk
CV Number (if applicable):	
Will this person be analysing the data? (Y/N)	N

Title:	Dr

Full name:	Christodoulos Kypridemos
Job title:	Senior Lecturer
Affiliation/organisation:	Department of Public Health and Policy, University of Liverpool
Email address:	ckyprid@liverpool.ac.uk
CV Number (if applicable):	
Will this person be analysing the data? (Y/N)	Y

Title:	Dr
Full name:	Pietà Schofield
Job title:	Research Associate
Affiliation/organisation:	Department of Public Health and Policy, University of Liverpool
Email address:	ckyprid@liverpool.ac.uk
CV Number (if applicable):	
Will this person be analysing the data? (Y/N)	Y

Experience/expertise available

List below the member(s) of the research team who have experience with CPRD data.

Dr Kate Fleming
Dr Pietà Schofield

List below the member(s) of the research team who have statistical expertise.

**Name(s):**
Dr Christodoulos Kypridemos
Dr Kate Fleming
Prof Martin O'Flaherty
Dr Pietà Schofield

List below the member(s) of the research team who have experience of handling large datasets (greater than 1 million records).

**Name(s):**
Dr Kate Fleming

Dr Christodoulos Kypridemos
Dr Pietà Schofield

List below the member(s) of the research team, or supporting the research team, who have experience of practicing in UK primary care.

**Name(s):**

ACCESS TO THE DATA

Sponsor of the study

Institution/Organisation:	University of Liverpool
Address:	Department of Public Health and Policy, 3rd Floor, Whelan Building, University of Liverpool, Brownlow Hill, Liverpool, L69 3GB

Funding source for the study

Same as Sponsor?	Yes	X	No	
Institution/Organisation:	University of Liverpool			
Address:	Department of Public Health and Policy, 3rd Floor, Whelan Building, University of Liverpool, Brownlow Hill, Liverpool, L69 3GB			

Institution conducting the research

Same as Sponsor?	Yes	X	No	
Institution/Organisation:	University of Liverpool			
Address:	Department of Public Health and Policy, 3rd Floor, Whelan Building, University of Liverpool, Brownlow Hill, Liverpool, L69 3GB			

Data Access Arrangements

Indicate with an ‘**X**’ the method that will be used to access the data for this study:

Study-specific Dataset Agreement	

Institutional Multi-study Licence	X
Institution Name	University of Liverpool
Institution Address	Brownlow Hill, Liverpool, L693BX

Will the dataset be extracted by CPRD?

Yes		No	X

If yes, provide the reference number:

INFORMATION ON DATA

Primary care data (place '**X**' in all boxes that apply)

CPRD GOLD		CPRD Aurum	X

X

Reference number (if applicable):

Please select any linked data or data products being requested

Patient Level Data (place '**X**' in all boxes that apply)

ONS Death Registration Data			
HES Admitted Patient Care			
HES Outpatient			
HES Accident and Emergency		NCRAS Cancer Registration Data	
HES Diagnostic Imaging Dataset		NCRAS Cancer Patient Experience Survey (CPES) data	
HES PROMS (Patient Reported Outcomes Measure)		NCRAS Systemic Anti-Cancer Treatment (SACT) data	
CPRD Mother Baby Link		NCRAS National Radiotherapy Dataset (RTDS) data	
Pregnancy Register		NCRAS Quality of Life Cancer Survivors Pilot (QOLP)	

Mental Health Data Set (MHDS)		NCRAS Quality of Life Colorectal Cancer Survivors (QOLC)	

Area Level Data (place '**X**' in one Practice / Patient level box that may apply)

**Practice level (UK)**		**Patient level (England only)**	
Practice Level Index of Multiple Deprivation		Patient Level Index of Multiple Deprivation	X
Practice Level Index of Multiple Deprivation<br>(index other than the most recent)		Patient Level Index of Multiple Deprivation Domains	
Practice Level Index of Multiple Deprivation Domains		Patient Level Carstairs Index for 2011 Census	
Practice Level Carstairs Index for 2011 Census (Excluding Northern Ireland)		Patient Level Townsend Score	
2011 Rural-Urban Classification at LSOA level		2011 Rural-Urban Classification at LSOA level	

Reference / Protocol number (where applicable):

CPRD reference 00040357

Are you requesting linkage to a dataset not listed above?

Yes		No	**X**

If yes, provide the Non-Standard Linkage reference number:

Does any person named in this application already have access to any of these data in a patient identifiable form, or associated with an identifiable patient index?

Yes		No	**X**

If yes, provide further details:

VALIDATION/VERIFICATION

Does this protocol describe an observational study using purely CPRD data?

Yes	**X**	No	

Does this protocol involve requesting any additional information from GPs, or contact with patients?

Yes		No	**X**

If yes, provide the reference number:

PART 2: PROTOCOL INFORMATION

Applicants must complete all sections listed below<br><br>Applications with sections marked ‘Not applicable’ without justification will be returned as invalid
Study Title (Max. 255 characters, including spaces)<br><br>Equity dynamics of multimorbidity in England over time: a descriptive study on trends of incident and prevalent multimorbidity by age, sex, and socioeconomic status
Lay Summary (Max. 250 words)<br><br>In the UK, an increasing number of people are living with multiple chronic conditions (multimorbidity), such as with diabetes, heart disease and dementia. Those from socioeconomically deprived backgrounds are more affected, and more likely to develop multiple conditions at earlier ages. Individuals with multiple conditions tend to have worse health, multiple medications, and often experience discontinuity of care. Multimorbidity is also a problem for health systems, which are focused around the care of single conditions. Although associated with ageing, many people of working age also live with multimorbidity. Current studies have not looked at how multimorbidity in the English adult population has changed in recent years.<br><br>This study will look at the number of patients with multiple long-term conditions between 2004-2018 to see how trends have changed over time, as well as by age, gender, and socioeconomic status. We are particularly interested in multimorbidity with three or more chronic conditions that affect three or more body systems, as this is considered an indicator of poorer health and greater healthcare usage. We will also look for patterns in the age of onset of multimorbidity and the time between developing a first disease and then subsequent conditions.<br><br>Describing these patterns will help us see how multimorbidity accumulates over the life-course and how this has evolved over time, and therefore can help to identify areas for focusing prevention efforts.

Technical Summary (Max. 300 words)

Background: Multimorbidity is becoming increasingly prevalent in the UK, with higher burden of disease and earlier onset among the more socioeconomically deprived. Multimorbidity poses unique challenges to the provision of primary care and the structure of healthcare systems currently focused around single conditions. Few studies have looked at multimorbidity incidence, trajectories of accumulation, or equity trends over time in England, and those that have tend to focus on older subsets of the population. Furthermore, the majority of studies have used a definition of coexistence of 2 or more diseases from varying predefined lists of chronic conditions.

Objective: to describe the current patterns of coexistence of multiple conditions treated in primary care over the life-course and over time, and how these patterns vary by socioeconomic status

Methods: Two measures of multimorbidity will be used throughout: a) basic multimorbidity: two or more chronic conditions; b) complex multimorbidity: at least three chronic conditions affecting at least three body systems. CPRD data will be linked to the English Index of Multiple Deprivation 2010 to allow analyses by socioeconomic status.

The first stage of this observational descriptive cohort study will calculate the observed incidence and prevalence of multimorbidity by sex, age group, and socioeconomic status over the time period of 2004-2018. We will calculate the median age of onset of initial condition and multimorbidity, along with the median accumulation time from first condition diagnosis to onset of multimorbidity.

In the second stage of the study, we will use survival analysis (parametric or semi-parametric frailty models) to calculate the modelled incidence and prevalence of multimorbidity by sex, age group, and socioeconomic status. Parametric or semi-parametric frailty models will also be used to map trends in age of onset of first incident condition and time of subsequent progression to multimorbidity by sex, socioeconomic status, and initial condition.

Primary outcomes: incidence and prevalence of multimorbidity. Secondary outcomes will include: a) age of onset of 1st condition, b) age of onset of multimorbidity, c) time between onset of 1st condition and onset of multimorbidity.

Outcomes to be Measured<br><br>Primary outcomes: incidence and prevalence of basic multimorbidity (two or more chronic conditions); incidence and prevalence of complex multimorbidity (three or more chronic conditions in three or more body systems).<br><br>Secondary outcomes include: a) age of onset of 1st condition; b) age of onset of basic multimorbidity; c) age of onset of complex multimorbidity; d) time from onset of 1st disease to onset of basic multimorbidity; e) time from onset of 1st disease to onset of complex multimorbidity; f) number of chronic conditions<br><br>All analyses will be stratified by sex, age group, and quintile of the English Index of Multiple Deprivation (IMD) 2010 (as a measure of socioeconomic status).
Objectives, Specific Aims and Rationale<br><br>The overall objective is to describe the current patterns of coexistence of multiple conditions treated in primary care over the life-course and over time, and how these patterns vary by socioeconomic status.<br><br>The research aims are to:<br><br>Describe the time trends in basic and complex multimorbidity incidence and prevalence by age, sex, and socioeconomic status.<br><br>Describe the patterns of accumulation of multimorbidity by sex, age group, socioeconomic status, and initial condition.<br><br>Estimate how much the increased level of basic and complex multimorbidity in more deprived populations is driven by 1) earlier age of onset of initial condition, and 2) faster accumulation of disease.<br><br>Rationale: An understanding of recent trends in incident and prevalent multimorbidity trends by socioeconomic status can help us see how multimorbidity has evolved over time. Looking at life-course trajectories and accumulation of multimorbidity may be useful for identifying opportunities to prevent and manage multimorbidity, helping to generate hypotheses as to how multimorbidity progresses and inform future research into the potential drivers of multimorbidity.

Study Background

Multimorbidity, defined by the WHO as “the coexistence of two or more chronic conditions in the same individual”1, is becoming increasingly prevalent in the UK and a continuing increase is projected2,3. Multimorbidity poses unique challenges to the provision of primary care and the structure of healthcare systems in which care is currently focused around single conditions4,5.

In 2008, an estimated 5m people in England had multimorbid conditions6. Despite the association between older age with an increasing number of chronic conditions, in absolute terms more people under age 65 live with multiple conditions than those over 657. Analysis of cross-sectional data has also shown that multimorbidity is presenting earlier8, and that this trend is predicted to continue3. A socioeconomic gradient has been found with multimorbidity: a study of patient records in Scotland found that patients from the most deprived areas were more likely to have multiple chronic conditions, to have developed multimorbidity 10-15 years earlier, and to have multimorbidity containing at least one mental health condition than those from the least deprived areas7.

A typical approach in operationalising multimorbidity for research is to count the conditions an individual has from a predefined list of diseases, however this simplifies the heterogeneity of disease clusters and implies that co-existing diseases are unrelated9. Based on evidence that occurrence of diseases in multiple body systems may have a large impact on health and healthcare use, Singer et al. propose a ‘complex multimorbidity’ measure, with the definition of at least three diseases in three or more body systems10.

Existing studies of multimorbidity using CPRD have focussed on pragmatic subsets of chronic conditions based on the Quality and Outcomes Framework and clinical relevance, resulting in lists ranging from 11 to 56 conditions. Recent work by Kuan et al [protocol 16_022] mapping 308 physical and mental health conditions using CPRD has made available the phenotyping algorithms and coding lists11, which will facilitate a more comprehensive list of chronic conditions in studies of multimorbidity.

Whilst there are existing studies that have looked at multimorbidity incidence, prevalence, and socioeconomic inequalities in England, these have either focused on subsets of the population (e.g. over-50s, patients with cardio-vascular disease12) or have not had an equity focus. The CPRD dataset provides an opportunity to describe the current picture of multimorbidity across the adult life-span and by socioeconomic status, in a representative sample of the primary care population in

England. Using the phenotyping algorithms provided by Kuan et al [protocol 16_022], this study will look at both basic and complex multimorbidity between 2004-2018 to describe equity and time trends in multimorbidity. An understanding of these trends can then be used to help inform prevention interventions to reduce the burden of multimorbidity on the health system.
Study Type<br><br>Descriptive epidemiology study.
Study Design<br><br>An observational descriptive cohort study design has been chosen as the primary objective is to describe trends in incidence and prevalence over time.
Feasibility counts<br><br>The study period will cover 2004-2018, with data from practices in England only to allow linkage to the English Index of Multiple Deprivation 2010.<br><br>To calculate incidence and prevalence of multimorbidity, we will include all patients with a patient acceptability flag from practices eligible for data linkage.<br><br>Expected number of patients with multimorbidity: approximately 400,000 patients in England (based on previous research from protocols 07-054R and 16-057R).<br><br>We will include all non-communicable diseases (NCDs) included in the work of Kuan et al, in addition to HIV and Hepatitis C as per the Academy of Medical Science definition of multimorbidity11. Read Code lists made available by Kuan et al (https://github.com/spiros/chronological-map-phenotypes) will be used for definitions of all included conditions.<br><br>The final list of conditions will be agreed by all members of the study team, which includes one epidemiologist and two clinicians.
Sample size considerations<br><br>This study is not testing a specific hypothesis. Our aim is to describe the knowledge of current trends in multimorbidity as defined above. Using all available linked data from Aurum will allow us the largest primary care population from which to extract this information.

Planned use of linked data (if applicable):

Linkage to the patient-level English Index of Multiple Deprivation 2015 to allow stratification of results by area-level socioeconomic status. IMD is an exposure of interest and so the entire study will be undertaken among practices which have consented to linkages.

Definition of the Study population

The study period will be from 1 Jan 2004 to 31 Dec 2018

The population will include permanently-registered adults (>=18 years of age) at practices in England for whom anonymised primary care records and data linkage (for socioeconomic deprivation information) are available

The study will use an open cohort design with patients entering the cohort when 1) they are aged 18 or over, 2) they have at least a one-year history of CPRD, and 3) the associated practice data is up-to-standard

Inclusion:

Permanently-registered adults (>=18 years of age)

Patients for which linked IMD data is available

Patients registered at a practice with up-to-standard data

Patients with at least 1-year of permanent registration

Patient records have an acceptable patient flag

Exclusion:

Patients <18 years of age

Patients without linked IMD data

Patients without a continuous 1-year registration prior to study period

Patients without a patient acceptability flag

NB: additional exclusion criteria apply to certain analyses, described below.

Selection of comparison group(s) or controls

Not applicable to study aims.

Exposures, Outcomes and Covariates

Exposures:

The key exposure of interest is quintiles of the English Index of Multiple Deprivation 2010 as a measure of socioeconomic status.

Case Definitions:

Basic multimorbidity: presence of two or more chronic conditions in an individual.

Complex multimorbidity: presence of three or more chronic conditions that affect three or more body systems.

We will use the Academy of Medical Sciences definition of chronic conditions: A physical non-communicable disease of long duration, such as a cardiovascular disease or cancer; A mental health condition of long duration, such as a mood disorder or dementia; or an infectious disease of long duration, such as HIV or hepatitis C".

We will include all long-term non-communicable diseases included in the work of Kuan et al [protocol 16_022], as well as chronic infections e.g. HIV and Hepatitis C. This work included the diseases in the quality and outcomes framework (QOF) of the UK GP contract 2018/19, in addition to those with high resource use. The list of conditions will be agreed by all members of the study team, which includes one epidemiologist and two clinicians. Read Code lists made available by Kuan et al (https://github.com/spiros/chronological-map-phenotypes) will be used for definitions of all included conditions.

Outcomes:

Primary outcomes: incidence and prevalence of basic multimorbidity (two or more chronic conditions) and complex multimorbidity (three or more chronic conditions in three or more body systems).

Secondary outcomes include: a) age of onset of 1st condition; b) age of onset of basic multimorbidity; c) age of onset of complex multimorbidity; d) time from onset of 1st disease to onset of basic multimorbidity; e) time from onset of 1st disease to onset of complex multimorbidity; f) number of chronic conditions

Covariates:

Data/ Statistical Analysis

Descriptive statistics:

All outcome variables, exposure variables and covariates will be tabulated to identify coding errors and missing data, and to view frequencies and distributions. Distributions of continuous variables will be considered by plotting histograms and comparing mean and median values. Mean and standard deviation will be presented for normally distributed variables, while median and interquartile range will be used for variables with skewed distributions.

Frequencies and percentages of all variables will be tabulated and compared visually between the complete dataset and the dataset restricted to those with IMD information.

*For all analyses date of multimorbidity onset will be operationalised as a) basic multimorbidity: date of diagnosis of a second chronic condition; b) complex multimorbidity: date of diagnosis of a third (or greater) chronic condition in a third body system.

Stage one: analyses directly observed from the data

Incidence:

Additional exclusion criteria: individuals with multimorbidity (as defined in the above case definitions of basic and complex multimorbidity) at entry into the study for each year.

Time at risk for each person will start at the latest of a) 1st day of the year, b) 1 year after date of permanent registration at the practice, c) practice up-to-standard date; and will end at the earliest of: a) last day of the year, b) date of death, c) date of transfer out of practice, d) date of multimorbidity onset*, e) last date of data collection at the practice.

Annual observed incidence rates will be tabulated directly from the data for each year from 2004-2018. To calculate annual incidence rates for basic and complex multimorbidity, the number of incident cases will be divided by the person-time at risk in the cohort for that year. Incidence rates will be calculated for the whole study population, and stratified by sex, 10-year age group, and IMD quintile. Results will be age- and sex- standardised using the European Standard Population 2013.

Prevalence:

Person-time for each patient will start at the latest of a) 1st day of the year, b) 1 year after date of permanent registration at the practice, c) practice up-to-standard date; and will end at the earliest of: a) last day of the year, b) date of death, c) date of transfer out of practice, d) last date of data collection at the practice.

Annual observed prevalence rates will be tabulated directly from the data for each year from 2004-2018. To calculate annual prevalence for basic and complex multimorbidity the number of prevalent cases will be divided by the total person-time in the cohort for that year. Prevalence will be calculated for the whole study population, and stratified by sex, 10-year age group, and IMD quintile. Results will be age- and sex- standardised using the European Standard Population 2013.

Median age of onset of initial condition:

Additional exclusion criteria: individuals who have one or more condition of interest at time of entry to the study.

Time of entry to the study will be defined as the latest of when: a) 1 January 2004, b) the individual is aged 18 or over, c) the individual has at least a one-year history of CPRD, and d) the associated practice data is up-to-standard.

Median age (and interquartile range) at which an initial condition was diagnosed will be calculated for the whole population, and stratified by IMD quintiles and sex.

Median age of onset of multimorbidity*:

Additional exclusion criteria: individuals with multimorbidity (as defined in the above case definitions of basic and complex multimorbidity) at entry into the study for each year.

Time of entry to the study will be defined as the latest of when: a) 1 January 2004, b) the individual is aged 18 or over, c) the individual has at least a one-year history of CPRD, and d) the associated practice data is up-to-standard.

Median age (and interquartile range) of multimorbidity onset* will be calculated for the whole population, and stratified by IMD quintiles, sex, and initial condition.

Median time of accumulation from initial disease onset to multimorbidity onset*

Additional exclusion criteria: individuals who have one or more condition of interest at time of entry to the study.

Time of entry to the study will be defined as the latest of when: a) 1 January 2004, b) the individual is aged 18 or over, c) the individual has at least a one-year history of CPRD, and d) the associated practice data is up-to-standard.

Median number of days between diagnosis of initial condition and onset of multimorbidity* will be calculated, and presented with the interquartile range, for the whole study population and stratified by sex, age group (age at onset of initial condition), IMD quintile, and initial condition.

Stage two: analyses from statistical models

Incidence and prevalence:

Annual incidence and prevalence will be modelled using semi-parametric or parametric cluster/nested frailty models. The approximate likelihood cross-validation criterion (LCVa) will be used in informing choice of the model that best fits the data13. The hierarchy of clustering will be GP practices within regions. The nested and cluster models will be compared and the simpler cluster model will be chosen if there is little difference in model fit.

Prevalence and incidence rates will be calculated for the whole study population, and stratified by sex, 10-year age group, and IMD quintile. Results will be age- and sex- standardised using the European Standard Population 2013.

i) Incidence:

Additional exclusion criteria: individuals with multimorbidity (as defined in the above case definitions of basic and complex multimorbidity) at entry into the study for each year.

Time at risk for each person will start at the latest of a) 1st day of the year, b) 1 year after date of permanent registration at the practice, c) practice up-to-standard date; and will end at the earliest of: a) last day of the year, b) date of death, c) date of transfer out of practice, d) date of multimorbidity onset*, e) last date of data collection at the practice.

ii) Prevalence:

Time at risk for each person will start at the latest of a) 1st day of the year, b) 1 year after date of permanent registration at the practice, c) practice up-to-standard date; and will end at the earliest of: a) last day of the year, b) date of death, c) date of transfer out of practice, d) last date of data collection at the practice. Prevalence will be calculated for the whole study population, and stratified by sex, 10-year age group, and IMD quintile. Results will be age- and sex- standardised using the European Standard Population 2013.

Survival analyses

In the below analyses, parametric or semi-parametric frailty models will be used to account for unobserved heterogeneity within practices and regions. The gamma distribution will be used for the frailty model. The hierarchy of clustering will be GP practices within regions. For each analysis, the nested and cluster models will be compared and the simpler cluster model will be chosen if there is little difference in model fit. Likelihood ratio tests will be used to test for time-dependent covariates. Death will be included as the terminal event in the joint models. The joint frailty model will be compared to the simpler shared frailty model (not including a terminal event) for each analysis, and the simpler cluster model will be chosen if there is little difference in model fit. Choice of the final model structure for each analysis will be informed by the approximate likelihood cross-validation criterion (LCVa)13.

i) Age of 1st disease onset:

Additional exclusion criteria: individuals who have one or more condition of interest at time of entry to the study.

Time of entry to the study will be defined as the latest of when: a) 1 January 2004, b) the individual is aged 18 or over, c) the individual has at least a one-year history of CPRD, and d) the associated practice data is up-to-standard.

Time of exit will be the earliest of age at: date of onset of initial condition, or censoring at: a) date of death, b) date of transfer out of practice, c) last date of data collection at the practice, d) 31 December 2018.

A semi-parametric or parametric joint cluster/nested frailty model will be used to model age at onset of 1st disease. Age will be used as the time scale for the model. Covariates will include sex,

IMD quintile, GP practice, and region. Analyses will be stratified by sex and IMD quintiles. Model outputs will then be used to estimate average age of onset by sex and IMD quintile.

ii) Age of multimorbidity onset and time to accumulation of multimorbidity:

Additional exclusion criteria: individuals who have one or more condition of interest at time of entry to the study.

Time of entry to the study will be defined as the latest of: a) 1 January 2004, b) when the individual is aged 18 or over, c) the individual has at least a one-year history of CPRD, and d) when the associated practice data is up-to-standard.

Time of exit will be the earliest of: date of onset of multimorbidity*, or censoring at: a) date of death, b) date of transfer out of practice, c) last date of data collection at the practice, d) 31 December 2018.

A semi-parametric or parametric joint cluster/nested frailty model will be used to model i) age at onset of multimorbidity* and ii) time from onset of initial condition to accumulation of multimorbidity*. Diagnosis of each new disease (for complex multimorbidity: each new disease in a new body system) will be the recurrent event in the joint model. Age will be used as the time scale for the model. Sex, IMD quintile, GP practice, region, age of initial disease onset, and initial condition will be considered for inclusion in the model. Model outputs will then be used to estimate average age of multimorbidity onset and time (in days) to accumulation of multimorbidity by sex and IMD quintile. Analyses will be stratified by sex, IMD quintile and initial condition.

For each IMD quintile, we will calculate the ratio of a:b for a) time (in age) to first condition : b) time (in age) to multimorbidity, for the whole study period, for the whole population and stratified by sex, age of initial onset, and select initial conditions.

Data management

Data will be stored in an encrypted partition of a University of Liverpool server that is only accessible through intranet and access will be restricted only to the co-applicants. Data will be encrypted, then decrypted and re-encrypted at each use. All data analysis will be conducted in R 3.6.1.

Plan for addressing confounding<br><br>This study is descriptive, and we are interested in describing overall socioeconomic inequalities rather than attempting to explain potential mechanisms.
Plans for addressing missing data<br><br>Only patients with a patient acceptability flag will be included so all patients will have age and sex information. Patient-level IMD is likely to be unavailable for some patients. Missingness patterns will be described and baseline characteristics of patients with and without IMD information will be compared visually. Patient records with missing IMD values will be excluded from all statistical analyses.
Patient or user group involvement<br><br>The new NIHR Applied Research Collaboration North West Coast has identified multiple morbidity and health across the life-course as two of its research priorities. Results of this study will be fed back through the ARC NWC Public Reference Panel. We will seek feedback from the panel on the lay summary of our research findings.
Plans for disseminating and communicating study results, including the presence or absence of any restrictions on the extent and timing of publication<br><br>We plan to present the findings: 1) in an appropriate peer reviewed scientific journal, 2) in Anna Head's PhD thesis, 3) at national/international academic meetings, 4) at appropriate internal meetings, and 5) in an article for a general public/non-academic audience. Stage 1 and stage 2 findings will be presented separately.<br><br>When disseminating and communicating the findings, we will ensure that we comply with CPRD policy regarding data presentation.<br><br>Conflict of interest statement: The investigators have no conflicts of interest to declare.

Limitations of the study design, data sources, and analytic methods

Coding – The quality of patient data in this study depends on the accuracy of coding by GPs.

Disease acquisition – The date of disease diagnosis will be used as the acquisition date of disease. However, we are aware that this is not necessarily the same as the actual disease onset date (i.e. patients may be asymptomatic with a long-term condition or may not present to healthcare for many years prior to diagnosis). In addition, disease diagnosis often leads to monitoring and further tests which increases the likelihood of acquisition of subsequent disease(s).

We will be unable to study accumulation patterns of multimorbidity for patients with one or more conditions at time of entry into the study population. This may introduce bias into our survival analyses as only a 'healthier' subset of patients can be studied, and follow-up time is relatively short for chronic conditions.

Data not transferred back to / recorded by GP – this depends on the recording practice of GPs.

Primary care data only – We are aware that under-reporting of conditions may occur through use of primary care data only, and that some secondary service diagnosis data for conditions such as asthma might be missed. However, we are focusing only on multimorbidity diagnosed and/or managed in primary care as the majority of multimorbidity management is undertaken by GPs, therefore this is likely to be a representative picture of the current burden within primary care.

Missing IMD data – IMD data is unlikely to be available for all patients – we anticipate approx. 1% of IMD values to be missing [protocol 15_120R]14. This may introduce bias into the study if those excluded from the analyses systematically differ from those included. Baseline characteristics of patients with and without IMD information will be compared, and missingness patterns will be described.

References

1. World Health Organization (WHO). Multimorbidity Technical Series on Safer Primary Care Multimorbidity: Technical Series on Safer Primary Care [Internet]. 2016 [cited 2019 May 13]. 28 p. Available from: http://apps.who.int/bookorders.

2. Dhalwani NN, O'Donovan G, Zaccardi F, Hamer M, Yates T, Davies M, et al. Long terms trends of multimorbidity and association with physical activity in older English population. Int J Behav Nutr Phys Act [Internet]. 2016 Dec 19 [cited 2019 May 13];13(1):8. Available from: http://www.ijbnpa.org/content/13/1/8

3. Kingston A, Comas-Herrera A, Jagger C, project M. Forecasting the care needs of the older population in England over the next 20 years: estimates from the Population Ageing and Care Simulation (PACSim) modelling study. 2018 [cited 2019 May 6]; Available from: www.thelancet.com/

4. Chris Salisbury ;, Bruce Guthrie, Peter Bower, Stewart W Mercer. How should health policy respond to the growing challenge of multimorbidity? [Internet]. 2018 [cited 2019 May 6]. Available from: http://www.bristol.ac.uk/media-library/sites/policybristol/PolicyBristol-Report-Oct18-health-challenge-multimorbidity.pdf

5. Van Weel C, Schellevis FG. Comorbidity and guidelines: Conflicting interests [Internet]. Vol. 367, Lancet. Elsevier; 2006 [cited 2019 May 14]. p. 550–1. Available from: https://www.thelancet.com/journals/lancet/article/PIIS0140-6736(06)68198-1/fulltext

6. Department of Health:Long term conditions. Long Term Conditions Compendium of Information: Third Edition [Internet]. Department of Health. 2012 [cited 2019 May 13]. Available from: http://www.dh.gov.uk/publications

7. Barnett K, Mercer SW, Norbury M, Watt G, Wyke S, Guthrie B. Epidemiology of multimorbidity and implications for health care, research, and medical education: a cross-sectional study. Lancet [Internet]. 2012 [cited 2019 May 10];380:37–43. Available from: www.thelancet.com

8. Katikireddi SV, Skivington K, Leyland AH, Hunt K, Mercer SW. The contribution of risk factors to socioeconomic inequalities in multimorbidity across the lifecourse: a longitudinal analysis of the Twenty-07 cohort. BMC Med [Internet]. 2017 Dec 24 [cited 2019 May 9];15(1):152. Available from: http://bmcmedicine.biomedcentral.com/articles/10.1186/s12916-017-0913-6

9. The Academy of Medical Sciences. Multimorbidity: a priority for global health research [Internet]. 2018 [cited 2019 May 6]. Available from: https://acmedsci.ac.uk/file-download/82222577

10. Singer L, Green M, Rowe F, Ben-Shlomo Y, Morrissey K. Social determinants of multimorbidity and multiple functional limitations among the ageing population of England, 2002–2015. SSM - Popul Heal [Internet]. 2019 May 30 [cited 2019 Jun 5];8:100413. Available from: https://www.sciencedirect.com/science/article/pii/S2352827319300278?via%3Dihub

11. Kuan V, Denaxas S, Gonzalez-Izquierdo A, Direk K, Bhatti O, Husain S, et al. A chronological map of 308 physical and mental health conditions from 4 million individuals in the English National Health Service. Lancet Digit Heal [Internet]. 2019 [cited 2019 May 23]; Available from: http://dx.doi.org/10.1016/S2589-7500

12. Tran J, Norton R, Conrad N, Rahimian F, Canoy D, Nazarzadeh M, et al. Patterns and temporal trends of comorbidity among adult patients with incident cardiovascular disease in the UK between 2000 and 2014: A population-based cohort study. Lam CSP, editor. PLoS Med [Internet]. 2018 Mar 6 [cited 2019 May 23];15(3):e1002513. Available from: https://dx.plos.org/10.1371/journal.pmed.1002513

13. Rondeau V, Mazroui Y, Gonzalez JR. frailtypack : An R Package for the Analysis of Correlated Survival Data with Frailty Models Using Penalized Likelihood Estimation or Parametrical Estimation . J Stat Softw. 2015;47(4).

14. Stevens S, Bankhead C, Mukhtar T, Perera-Salazar R, Holt TA, Salisbury C, et al. Patient-level and practice-level factors associated with consultation duration: A cross-sectional analysis of over one million consultations in English primary care. BMJ Open. 2017;7(11):1–7.

List of Appendices

It is impractical to provide each list of codes for each comorbidity under study. We will use the code lists produced and made freely available by Kuan et al, as detailed above.

Amendment Submitted 28 August 2020

Section J: We are only using data from the Aurum database; not both GOLD and Aurum as originally requested.

Section K: We are linking to IMD 2015; not IMD 2010 as originally requested.
